# Synthesis and evaluation of nitroheterocyclic aromatic adamantane amides with trypanocidal activity. Part II

**DOI:** 10.1039/d5md00527b

**Published:** 2025-11-06

**Authors:** Angeliki-Sofia Foscolos, Richard L. Atherton, Maria Billia, Markos-Orestis Georgiadis, Nuno Santarém, Anabela Cordeiro da Silva, Martin C. Taylor, John M. Kelly, Theodora Calogeropoulou, Andrew Tsotinis, Thomas Mavromoustakos, Ioannis P. Papanastasiou

**Affiliations:** a School of Health Sciences, Department of Pharmacy, Division of Pharmaceutical Chemistry, National and Kapodistrian University of Athens Panepistimiopoli-Zografou 15771 Athens Greece papanastasiou@pharm.uoa.gr; b Institute of Nanoscience & Nanotechnology, NCSR “Demokritos” 15341 Athens Greece; c Department of Infection Biology, London School of Hygiene and Tropical Medicine London WC1E7HT UK; d Faculty of Chemistry, Department of Organic Chemistry, National and Kapodistrian University of Athens Panepistimiopoli-Zografou 15771 Athens Greece; e Center for Drug Discovery and Department of Pharmaceutical Sciences, Northeastern University Boston MA 02115 USA; f Host-Parasite Interaction Group, i3S, Institute for Research and Innovation in Health, University of Porto Porto 4200-135 Portugal; g Laboratory of Microbiology, Department of Biological Sciences, Faculty of Pharmacy, University of Porto 4050-313 Porto Portugal; h Institute of Chemical Biology, National Hellenic Research Foundation 11635 Athens Greece

## Abstract

In this article, we report the design, synthesis, and biological evaluation of a new series of nitroheterocyclic aromatic adamantane amides targeting trypanosomes. These compounds feature diverse substituents on the adamantane scaffold, variations in side chain linker length, and a range of nitroheterocyclic moieties. This work represents a continuation of our previous efforts, with a particular focus on elucidating the structural and functional role of the linker connecting the phenyladamantane core to the nitroheterocyclic ring. The structure–activity relationship data underscore the importance of strategic modifications in enhancing the pharmacological profile of these compounds against trypanosome parasites. Further modifications are recommended to optimize the physicochemical properties of the current derivatives to improve intracellular targeting of trypanosomatids, an important clinical stage in their life cycle.

## Abstract 2

New nitroheterocyclic adamantane amides display promising trypanocidal activity. The spacer length between the adamantylphenyl backbone and the nitroheterocyclic ring plays a crucial role in potency.

## Graphical abstract

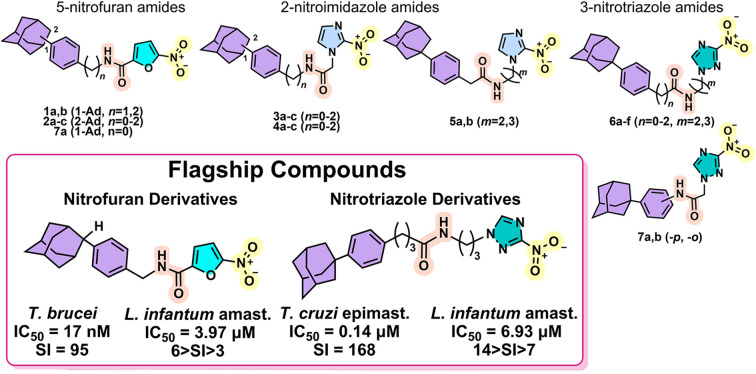

## Introduction

The protozoan parasites *Leishmania* spp., *Trypanosoma cruzi*, and *Trypanosoma brucei*, collectively known as the Tritryps, are the causative agents of the neglected tropical diseases (NTDs) leishmaniasis, Chagas disease (CD), and human African trypanosomiasis (HAT), respectively. Collectively, these diseases contribute to some of the highest mortality rates among all NTDs. Recognizing their impact, the World Health Organization (WHO) has designated them as high-priority diseases requiring “innovative and intensified disease management (IDM)”. This designation stems from several factors, including the complex and poorly understood disease burden, the high treatment costs, the lack of effective control strategies, and the insufficient investment in research and development.^1^ Common features of the current chemotherapeutic arsenal against these diseases include severe side effects, significant drug resistance, non-oral administration, and prolonged treatment regimens.^2^

Several of the currently available trypanocidal agents are nitroaromatic compounds: nifurtimox (Nfx) and benznidazole (Bzn) are the main treatments for CD, while nifurtimox is also used in combination therapy for the second stage of HAT.^3^ More recently, fexinidazole (Fxd) has replaced suramin and melarsoprol, as the first-line treatment, for individuals aged 6 years and older, who weigh 20 kg or more.^4^

Nifurtimox and benznidazole are prodrugs that undergo activation *via* a NADH-dependent, mitochondrially localized, bacterial-like type I nitroreductase (NTR).^5^ Moreover, NTR plays a crucial role in the activation of fexinidazole and its metabolites in *L. donovani*.^6^ Notably, the bacterial-like type I NTR, which bioactivates these nitroaromatic drugs, lacks homologs in mammals—a characteristic that contributes to their selective activity against trypanosomatids. The successful use of nitroaromatic drugs in treating trypanosomatid diseases has further encouraged the investigation of nitro derivatives for their chemotherapeutic potential, despite ongoing safety concerns.^7^

**Fig. 1:**
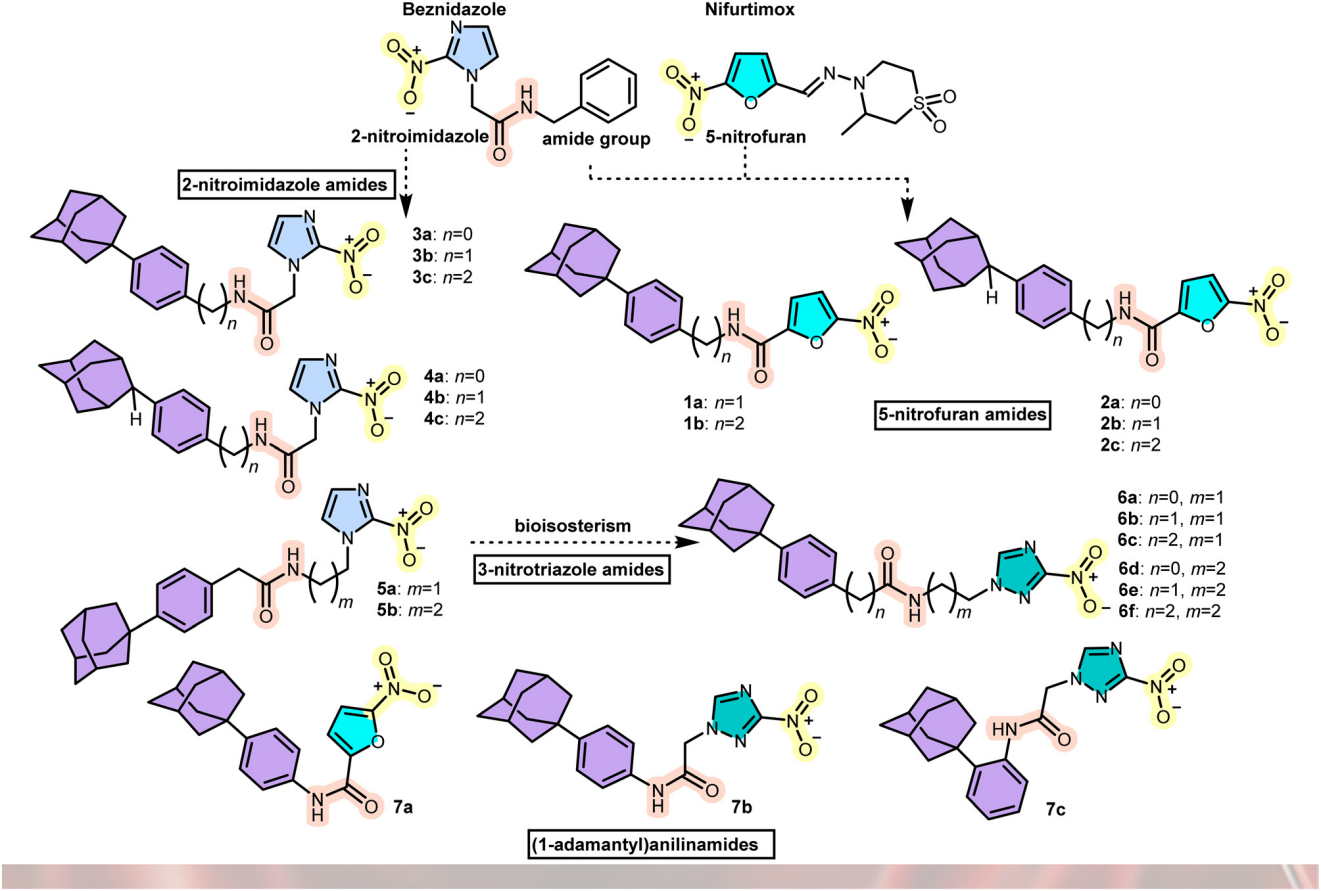
Nitroheterocyclic aromatic adamantane amides.

Based on the promising findings of our initial work on *N*-[4-(1-adamantyl)phenylalkyl]-5-nitrofuran-2-carboxamides 1a–c against *T. brucei* and *T. cruzi*,^8^ we report herein the synthesis and the trypanocidal evaluation of the nitroheterocyclic aromatic adamantane amides, 2a–c, 3a–c, 4a–c, 5a,b, 6a–f and 7a–c (Fig. 1).

As a structural variation of the *N*-[4-(1-adamantyl)phenylalkyl]-5-nitrofuran-2-carboxamides 1a,b, we synthesized the congeneric C-2 adducts, 2a–c. Both nitroimidazoles and bicyclic nitroimidazoles are well known for their antitrypanosomal activity.^9^ Building on the benznidazole scaffold, we also synthesized C-1 and C-2 adamantane hybrids, the reverse amides 3a–c and 4a–c, respectively. The (CH_2_)_*n*_ spacer length between the adamantane cage and the aromatic ring varies from 0 to 2 methylene units in this series of derivatives. Additionally, we explored the effect of the spacer length between the nitroheterocyclic moiety and the amido group, producing adducts 5a and b. In addition, based on previous reports on the antitrypanosomal activity of nitro-triazole derivatives,^10,11^ we replaced the (2-nitro-1*H*-imidazol-1-yl) functional group with the (3-nitro-1*H*-1,2,4-triazol-1-yl) moiety in the adducts 6a–f, to further probe how the spacer length affects the amide bond's positioning relative to both ends of the side chain. Lastly, we compared the (1-adamantyl)anilinamides with different heterocyclic rings (7a and 7b) or different aromatic substitutions (*para*-7b*vs. ortho*-7c).

## Results and discussion

### Chemistry

**Scheme 1:**
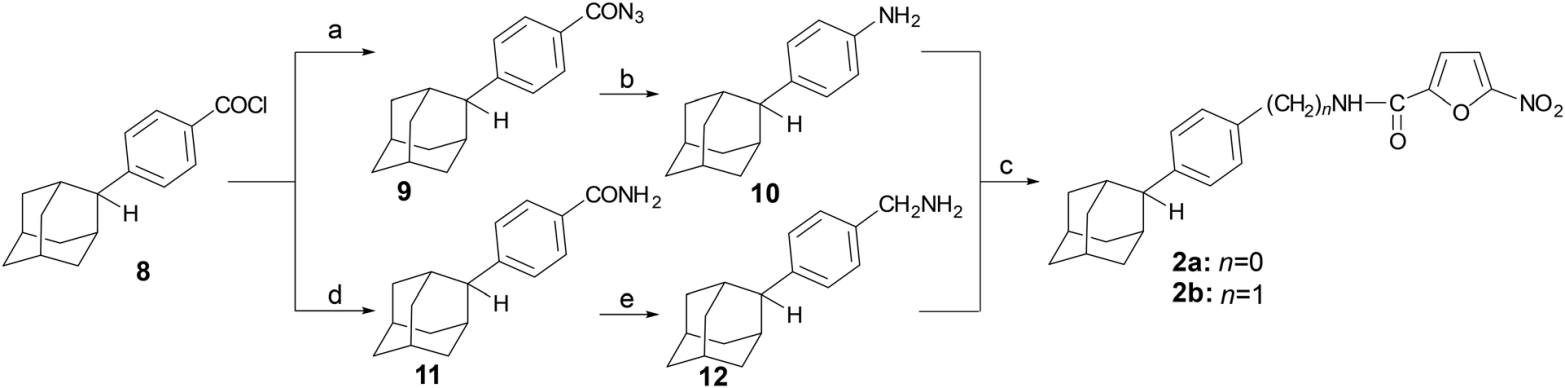
Reagents and conditions: a) NaN_3_, acetone/water, 0 °C, 0.5 h, b) i. toluene, 95 °C, ii. HCl 36%, 95 °C, 1 h, 54% (from 8) c) 5-nitro-2-furoic acid, HBTU, DIPEA, DCM/DMF 1 : 1 (v/v), RT, 24 h, 78% (2a), 70% (2b), d) NH_3_, THF, 0 °C then RT, 0.5 h, 71%, e) LiAlH_4_, THF, RT, 24 h, 66%.

The preparation of analogues 2a,b was accomplished *via* the reaction sequence shown in Scheme 1. 4-(2-Adamantyl)benzoyl chloride (8)^8^ was converted to the corresponding benzoyl azide 9, which upon heating at 95 °C in toluene underwent Curtius rearrangement to give 4-(2-adamantyl)aniline (10) *via* the acidic hydrolysis of the intermediate alkyl isocyanate. The benzoyl chloride 8 (ref. 8) was treated with ammonia to afford the corresponding benzamide 11, which was then reduced with LiAlH_4_, to the corresponding methanamine 12.

Both amines 10 and 12 (ref. 12) were converted into the desired *N*-substituted carboxamides 2a–b*via* amidation with 5-nitro-furoic acid, using 2-(1*H*-benzotriazol-1-yl)-1,1,3,3-tetramethyluronium hexafluorophosphate (HBTU) in the presence of diisopropylethylamine (DIPEA).

**Scheme 2:**
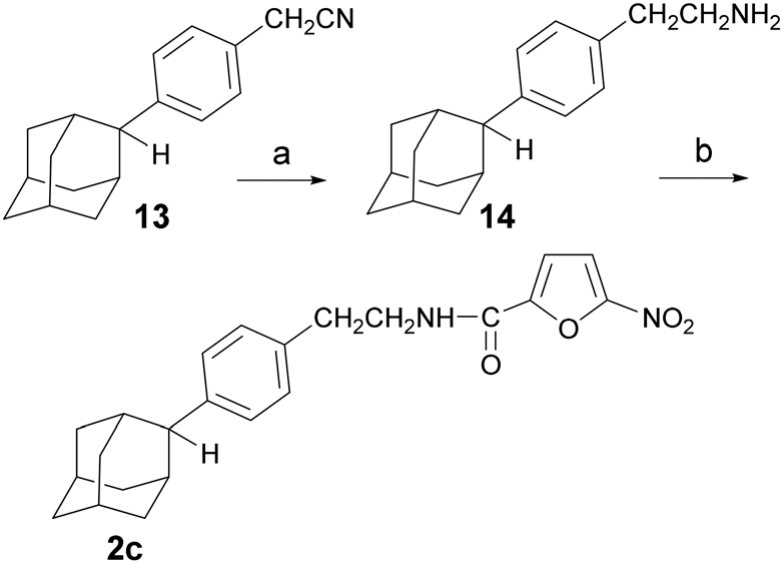
Reagents and conditions: a) BMS 2 M in toluene, THF, RT for 24 h then 50 °C for 5 h, 81%, b) 5-nitro-2-furoic acid, HBTU, DIPEA, DCM/DMF 1 : 1 (v/v), RT, 24 h, 76%.

The synthesis of the *N*-[4-(2-adamantyl)phenethyl]-5-nitrofuran-2-carboxamide (2c) is outlined in Scheme 2. The reduction of the phenylacetonitrile 13 (ref. 13) to the corresponding amine was a key step in this process. Several reduction protocols were tested, including the use of lithium aluminum hydride and catalytic hydrogenation in the presence of RANEY®-Ni (*vide* SI). However, both approaches failed to produce the desired amine 14. After considerable experimentation, the desired reduction was effected with borane dimethylsulfide, affording the ethanamine 14, which was then converted into the desired carboxamide 2c, *via* the previously described method.

**Scheme 3:**
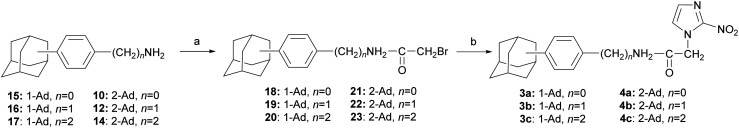
Reagents and conditions: a) BrCH_2_COCl, Na_2_CO_3_, CHCl_3_ – H_2_O, 0 °C to RT, 48 h, b) sodium 2-nitroimidazol-1-ide, DMF, 80 °C, 48 h, 35% over two steps (3a from 15), 38% (3b from 16), 33% (3c from 17), 23% (4a from 10), 26% (4b from 12), 31% (4c from 14).

The 2-nitroimidazole acetamides 3a–c and 4a–c were obtained by the bromoacetylating the corresponding amines 10,^12^12, and 14–17,^14,15^ using bromoacetyl chloride in a biphasic chloroform/water system in the presence of sodium carbonate. The resulting bromoacetamides 18–23 were then coupled with the sodium salt of 2-nitroimidazole (prepared from 2-nitroimidazole and sodium hydride), as demonstrated in Scheme 3.

**Scheme 4:**
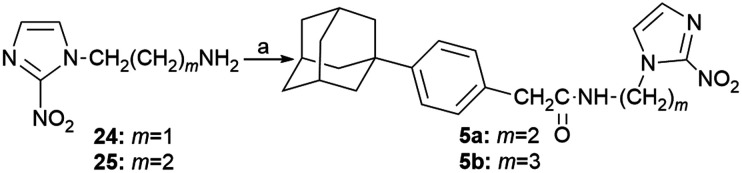
Reagents and conditions: a) 2-[4-(1-adamantyl)phenyl]acetyl chloride, Et_3_N, DCM, RT, 24 h, 62% (5a), 58% (5b).

The *N*-alkyl-2-nitroimidazole acetamides 5a–b were prepared by coupling the appropriate amines 24, 25 (ref. 16 and 17) with 2-[4-(1-adamantyl)phenyl]acetyl chloride,^14^ as shown in Scheme 4.

**Scheme 5:**
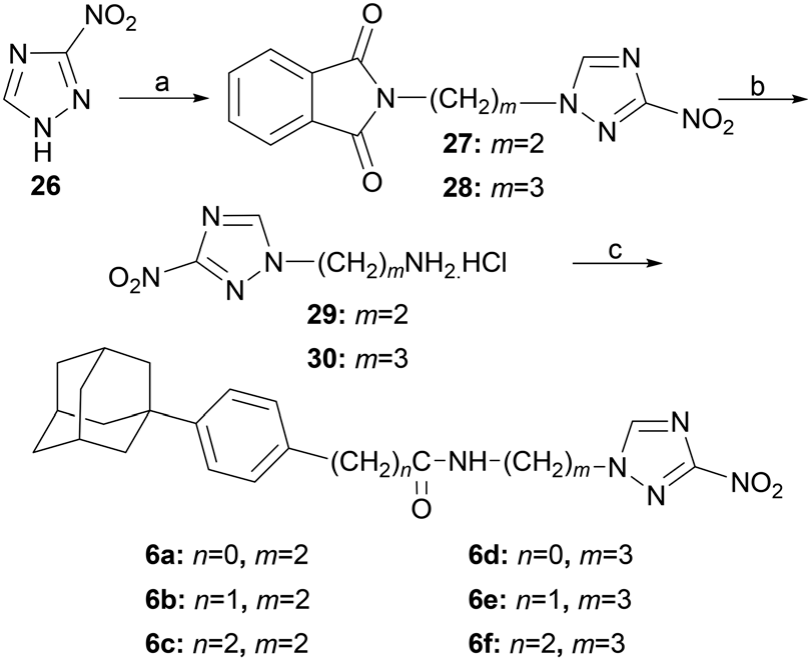
Reagents and conditions: a) 1. NaH, DMF, RT, 1.5 h, 2. 20: *N*-(2-bromoethyl)phthalimide, RT, 5 d, 21: *N*-(3-bromopropyl)phthalimide, RT, 5 d, b) 1. hydrazine hydrate, EtOH, reflux, 2 h, 2. HCl 2 N in EtOH, 80% (29 from 26), 76% (30 from 26) c) HBTU, DIPEA, DCM/DMF 1 : 1 (v/v), RT, 24 h for 29 and 4–(1-adamantyl)benzoic acid, 71% (6a), 29 and 2-[4-(1-adamantyl)phenyl]acetic acid, 64% (6b), 29 and 3-[4-(1-adamantyl)phenyl]propanoic acid, 76% (6c), for 30 and 4-(1-adamantyl)benzoic acid, 68% (6d), 30 and 2-[4-(1-adamantyl)phenyl]acetic acid, 72% (6e), 30 and 3-[4-(1-adamantyl)phenyl]propanoic acid, 76% (6f).

The 3-nitrotriazole amides 6a–f were synthesized as depicted in Scheme 5. Alkylation of 3-nitro-1*H*-1,2,4-triazole (26) with *N*-(2-bromoethyl)phthalimide or *N*-(3-bromopropyl)phthalimide led to the corresponding diones 27, 28, which upon hydrazinolysis afforded the desired amines 29, 30, respectively. Interestingly, the amine 29 was obtained in 80% yield, compared to the reported 69%.^18^

These amines were then coupled with the appropriate carboxylic acids 4-(1-adamantyl)benzoic acid,^19^ 2-[4-(1-adamantyl)phenyl]acetic acid,^14^ and 3-[4-(1-adamantyl)phenyl]propanoic acid,^14^ as previously shown, to give the desired benzamides 6a–f.

**Scheme 6:**
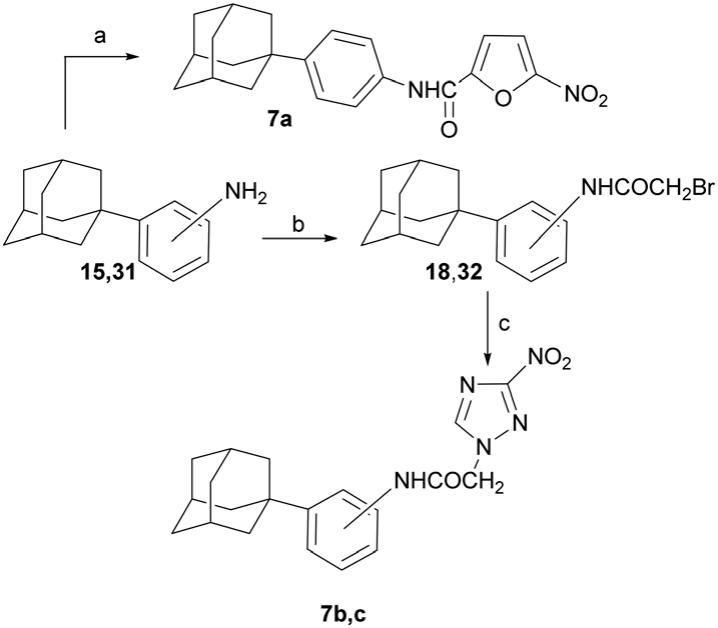
Reagents and conditions: a) 5-nitro-2-furoic acid, HBTU, DIPEA, DCM/DMF 1 : 1 (v/v), RT, 24 h, 76%; b) BrCH_2_COCl, Na_2_CO_3_, CHCl_3_ – H_2_O, 0 °C to RT, 48 h, 84–89%; c) sodium 3-nitro-1,2,4-triazol-1-ide, DMF, 50 °C, 48 h, 52–91%.

The synthesis of the (1-adamantyl)anilinamides 7a–c is illustrated in Scheme 6.

The *N*-substituted-5-nitrofuran-2-carboxamide 7a was synthesized *via* amidation of the 4-(1-adamantyl)aniline (15)^14^ with 5-nitrofuroic acid, using the protocol described for carboxamides 2a–c. Aniline 15 and 2-(1-adamantyl)aniline 31 (ref. 20) were bromoacylated to the corresponding amides 18, 33. The latter acetamides led to the desired anilamides 7a–c, as shown above.

### Pharmacology

The nitroheterocyclic adamantane amides 2a–c, 3a–c, 4a–c, 5a,b, 6a–f and 7a–c were tested for their activity against the bloodstream-form *T. brucei*, *T. cruzi* epimastigotes, intracellular amastigotes and *L. infantum* promastigotes and intracellular amastigotes. The results are listed in Tables 1–3.

**Table 1:** Activity against bloodstream-form *Trypanosoma brucei*

cmpd	*T. brucei* IC_50_^a^ (μM)	*T. brucei* IC_90_^a^ (μM)	L6† cells IC_50_ (μM)	S.I.^b^
2a	0.13 ± 0.04	0.33 ± 0.02	0.47 ± 0.07	4
2b	0.017 ± 0.003	0.031 ± 0.001	1.6 ± 0.1	95
2c	0.042 ± 0.008	0.073 ± 0.008	1.8 ± 0.2	42
6a	0.23 ± 0.05	0.36 ± 0.03	28 ± 7	120
6b	0.42 ± 0.07	1.8 ± 0.2	30 ± 2	72
6c	0.20 ± 0.02	0.32 ± 0.01	23 ± 2	114
6d	0.18 ± 0.05	0.31 ± 0.04	22 ± 1	123
6e	0.24 ± 0.01	0.40 ± 0.01	23 ± 3	96
6f	0.14 ± 0.02	0.28 ± 0.01	19 ± 2	134
7a	0.091 ± 0.005	0.22 ± 0.01	<0.03	
7b	0.20 ± 0.07	0.91 ± 0.19	7.1 ± 0.3	35
7c	>25	>25	—	—
Nfx	4.4 ± 0.7 (ref. 21)	—	32. ± 0.1 (ref. 21)	7
Fxd	2.4 ± 0.2^c^	—		>333 (ref. 22)

a IC_50_ and IC_90_: concentrations that inhibit growth by 50 and 90%, respectively.

b Selectivity index: ratio of IC_50_ values obtained with L6 cells and *T. brucei*.

c Fexinidazole was tested under the same conditions as for the reported derivatives.

**Table 2:** Activity against *Trypanosoma cruzi* epimastigotes and intracellular amastigotes

cmpd	*T.* cruzi (epimastigotes) IC_50_^a^ (μM)	*T.* cruzi (epimastigotes) IC_90_^a^ (μM)	*T. cruzi* (intracellular amastigotes) IC_50_^a^ (μM)	*T. cruzi* (intracellular amastigotes) IC_90_^a^ (μM)	L6† cells IC_50_ (μM)	S.I.^b^ (epimastigotes) IC_50_/Tc IC_50_	S.I.^b^ (intracellular) IC_50_/Tc IC_50_
2a	0.55 ± 0.05	1.2 ± 0.03			0.47 ± 0.07	—	
2b	0.56 ± 0.02	1.2 ± 0.02			1.6 ± 0.1	3	
2c	0.28 ± 0.08	1.2 ± 0.08			1.8 ± 0.2	6	
6a	0.54 ± 0.02	1.2 ± 0.01	4.88 ± 0.51	21.4 ± 3.4	28 ± 7	51	6
6b	0.20 ± 0.03	0.66 ± 0.04	1.83 ± 0.70	4.29 ± 0.31	30 ± 2	154	16
6c	0.45 ± 0.02	1.2 ± 0.02	1.58 ± 0.07	3.23 ± 0.85	23 ± 2	50	15
6d	0.45 ± 0.01	1.1 ± 0.02	3.73 ± 0.44	8.42 ± 0.44	22 ± 1	49	6
6e	0.14 ± 0.01	0.27 ± 0.02	2.48 ± 0.31	7.08 ± 0.47	23 ± 3	168	9
6f	0.44 ± 0.02	0.91 ± 0.01	2.44 ± 0.57	8.23 ± 1.78	19 ± 2	43	8
7a	>0.025	>0.025			<0.03		
7b	—	—			7.1 ± 0.3	—	
7c	>25	>25			—	—	
Nfx	3.1 ± 0.5 (ref. 23)	—			32. ± 0.1 (ref. 21)	10	
Bzn	5.4 ± 0.2^c^	—	1.04 ± 0.32	2.54 ± 0.81	510 ± 22 (ref. 21)	95	490

a IC_50_ and IC_90_: concentrations that inhibit growth by 50 and 90%, respectively.

b Selectivity index: ratio of IC_50_ values obtained with L6 cells and *T. cruzi*.

c Benznidazole was tested under the same conditions as for the reported derivatives.

**Table 3:** Activity against *Leishmania infantum* promastigotes and intracellular amastigotes, toxicity against THP1 cells and selectivity index

cpmd	*L. infantum* (promastigotes)	*L. infantum* (intracellular amastigotes)		THP-1 CC_50_ (μM)	SI (promastigotes) CC_50_/Li IC_50_	SI (intracellular amastigotes) CC_50_/Li ia IC_50_
	IC_50_ (μM), 95% CI	% of growth inhibition at 10 μM ± ST DEV (μM, *n* ≥ 3, SD)	IC_50_ (μM), 95% CI			
1a	3.46 (2.56 to 4.57)	Nt		CC_50_ > 100	SI > 29	
1b	0.66 (0.51 to 0.83)	Na		100 > CC_50_ > 50	151 > SI > 76	
2a	0.12 (0.094 to 0.16)	Na		6.25 > CC_50_ > 1.56	52 > SI > 26	
2b	0.09 (0.094 to 0.16)	84.6 ± 15.6	3.97 (3.11 to 5.05)	25 > CC_50_ > 12.5	272 > SI > 136	6 > SI > 3
2c	0.05 (0.05 to 0.06)	Na		12.5 > CC_50_ > 3.13	232 > SI > 58	
3a	0.70 (0.50 to 0.96)	Na		100 > CC_50_ > 25	143 > SI > 36	
3b	13.81 (12.14 to 15.66)	Na		100 > CC_50_ > 50	7 > SI > 4	
3c	22.13 (19.69 to 24.92)	Na		100 > CC_50_ > 50	4 > SI > 2	
4b	>40	Na		CC_50_ > 100	—	
4c	37.87 (30.96 to 49.04)	Na		CC_50_ > 50	SI > 1	
5a	3.48 (2.84 to 4.24)	Na		50 > CC_50_ > 25	3 > SI > 14	
5b	4.81 (2.84 to 7.42)	Na		100 > CC_50_ > 50	21 > SI > 10	
6a	0.74 (0.59 to 0.92)	Na		50 > CC_50_ > 25	135 > SI > 34	
6b	0.49 (0.43 to 0.57)	50.1 ± 23.0		100 > CC_50_ > 50	204 > SI > 102	
6c	0.42 (0.35 to 0.50)	47.6 ± 16.3		CC_50_ > 100	SI > 238	
6d	0.27 (0.25 to 0.29)	Na		100 > CC_50_ > 50	370 > SI > 185	
6e	0.31 (0.26 to 0.36)	59.5 ± 12.0	6.93 (5.62 to 8.55)	100 > CC_50_ > 50	322 > SI > 161	14 > SI > 7
6f	0.39 (0.36 to0.43)	Na		100 > CC_50_ > 50	256 > SI > 128	
7a	0.76 (0.53 to 1.06)	Na		25 > CC_50_ > 6.25	33 > SI > 8	
MF	11.93 (11.36 to 12.54)	92 ± 5.0	2.65 (2.33 to 3.01)	29.99 (24.57 to 36.52)	2.5	11.3

From the results shown in Tables 1 and 2, it becomes apparent that among the 5-nitrofuran-2-carboxamides 2a–c, the derivatives bearing C-2 substituents on the adamantane ring exhibit higher activity compared to their C-1 substituted counterparts, 1a–c.^8^ The benzylamide derivative 2b and the phenylacetamide analogue 2c have IC_50_ values of 17 nM and 42 nM, respectively, against *T. brucei*. However, this trend is reversed against *T. cruzi* epimastigotes, where phenylacetamide 2c exhibits twice the activity of benzylamide 2b, with both being active in the sub-micromolar range. Conversely, their C-1 substituted congeners display, in general, activity, in a micromolar scale, with the exception of the anilamide 7a, which has an IC_50_ value of 91 nM against *T. brucei*, although this is likely due to toxicity, since it is also highly deleterious to the mammalian cells. Replacing the 5-nitrofuran unit at the terminal position of the tested amides with 2-nitroimidazole did not give the expected results, as derivatives 3a–c, 4a–c, and 5a,b were inactive (>25 μM). Similarly, inverting the amide bond in the lateral chain linking the 4-(1-adamantyl)phenyl scaffold to the 2-nitroimidazole moiety, had no effect on the antitrypanosomal activity, as the corresponding compounds 5a and 5b remained inactive. However, the replacement of the 2-nitro(1*H*-imidazol-1-yl) moiety with the 3-nitro(1*H*-1,2,4-triazol-1-yl) ring significantly enhanced the pharmacological activity of the tested compounds. A comparison of the triazole analogues reveals that the propyldiamino linker in the side chain induces stronger antitrypanosomal action than the ethyldiamino linker, as compounds 6d–f are more potent than 6a–c. However, both homologous series follow the same structure–activity relationship (SAR) pattern: when the carbonyl group is removed from the phenolic ring, the antitrypanosomal activity increases. The propanamides 6c and 6f are more active than the corresponding acetamides 6b and 6e, as well as the benzamides 6a and 6d. Within the acetamide and benzamide series, the trend is reversed, with benzamides 6a and 6d being more active than acetamides 6b and 6e, though the latter exhibit a better selectivity index (SI). The less cytotoxic derivatives were tested against intracellular *T. cruzi* amastigotes., their activity was found to be in the good to moderate range. Regarding the activity against intracellular amastigotes, the ethanediamino derivatives 6b and 6c show higher activity compared to the other nitrotriazole analogues.

The nitrofuramide 2b displays nanomolar-scale activity, and is 140–260 fold more potent against *T. brucei* than the reference drugs fexinidazole and nifurtimox. Regarding the anilamides 7a–c, the short side chain of 7a reduces trypanocidal activity. Comparing the two aromatic isomers 7b and 7c, it is evident that the steric hindrance in the *ortho*-substituted 7c also reduces activity.

The results shown in Table 3, with respect to *L. infantum*, point towards the same pharmacological profile for the adamantane nitroheterocyclic amides. Most of the tested molecules were active at sub micromolar potency against promastigotes. The C-2 adamantane 5-nitrofuran-2-carboxamides substituted analogues, 2a–c, exhibit higher activity than their C-1 substituted congeners 1a,b.^8^ High anti-parasitic potency of 2a–c was also observed for *T. brucei* (Table 2). However, 2a–c were more toxic to the THP-1 cells used as hosts for the intracellular infections, with 2a being the most cytotoxic molecule tested. Replacing the 2-nitrofuran-1-yl moiety (in derivatives 1a,b, 2a–b) with the 2-nitro-(1*H*-imidazol-1-yl) ring (derivatives 3a–c, 4a–c, 5a,b) did not improve antileishmanial activity. Notably, the 2-nitroimidazole reverse amide 5a was six-fold more potent than the corresponding 2-nitroimidazole amide 3c with the same linker length. Once again, the 3-nitro-1*H*-1,2,4-triazole adducts were more potent than the 2-nitro-1*H*-imidazole compounds. However, in this case, the length of the diamine spacer between the amide bond and the heterocycle is not a determining factor for antileishmanial activity, as the propanediamino adducts 6e–f were only slightly more potent than the ethanediamino derivatives 6a–c. Among the tested compounds, nitrofuramide 2c was the most potent against *L. infantum* promastigotes, whilst nitrotriazole 6d exhibited the best balance between promastigote activity and cytotoxicity. Nitrofuramide 2b has an equipotent SI to the nitrotriazole 6d, but exhibits better antileishmanial activity, close to that of the derivative 2c, the most potent compound tested against promastigotes. The nitrofuramide 2b was also active against *L. infantum* amastigotes, although the IC_50_ value (3.97 μM) was higher. The toxicity issues of the C-2 adamantane 5-nitrofuran-2-carboxamides substituted analogues, however, do not result in a favourable SI (3 to 6). Only three other molecules presented significant activity against intracellular amastigotes (6b, 6c and 6e). In fact, 6e was the most promising molecule with a predicted SI between 7 and 14. It is noteworthy, that 6e was among the most potent tested against *T. cruzi* (both epimastigotes and amastigotes) and the molecule with the best SI (Table 3). These indices facilitate the development of useful structure–activity relationships.

In addition to the biological evaluation, an *in silico* assessment of the compounds' drug-likeness and predicted toxicity was conducted to further explore their potential as drug candidates. Drug-likeness and toxicity predictions for the synthetic nitro amides were performed using the SwissADME and ProTox-3.0 platforms, respectively.

The physicochemical properties, drug-likeness, and toxicological profiles of the adamantylamide compounds were evaluated to provide a comprehensive understanding of their potential. These compounds display a lipophilic backbone while adhering to Lipinski's rule of five. Due to their high lipophilicity, the compounds exhibit low ESOL values, while maintaining favorable properties in terms of rotatable bonds, hydrogen bond donors and acceptors, and surface area (TSA). Minor variations based on structural differences were observed. All derivatives show excellent drug-likeness, as supported by the biological assays.

While pharmacologically promising, the nitro group (–NO_2_) in their backbone places them within the PAINS (Pan assay interference compounds) functional groups. Additionally, the presence of a long aliphatic carbon chain may increase toxicity, as such molecules are more likely to bind multiple targets. According to the Protox 3 platform, without accounting for potential prodrug activity, this structure predicts potential respiratory, toxicity, carcinogenicity, and mutagenicity. However, considering the presence of the nitro group and its potential for activation *via* metabolic processes, these predictions may not fully reflect the compound's *in vivo* behavior.

In a previous study,^24^ we have synthesized both nitro- and non-nitro furan derivatives, and the lack of activity observed in the non-nitro compounds indicated that the presence of the nitro group on the furan ring is essential for conferring trypanocidal activity. Based on this indirect evidence regarding the mechanism of action, along with the rational design of our compounds inspired by benznidazole, we hypothesize that the current derivatives act through a similar pathway to that of known commercial nitroheterocyclic drugs—namely, activation by parasite-specific nitroreductase enzymes.

Summary tables and a heatmap of the results for each compound are provided in the SI.

## Conclusions

The new nitroheterocyclic adamantane amides described in the current work display promising trypanocidal activity. The adamantane nitrofurans and nitrotriazoles were in general more potent and less toxic than their congeneric nitroimidazoles, even though the nitroimidazole moiety is the pharmacophore group in the established drugs fexinidazole and benznidazole. Among the new derivatives, the nitrofurane 2b demonstrated the highest activity against the bloodstream forms of *T. brucei*, *T. cruzi* epimastigotes, and *L. infantum* promastigotes. However, its reduced activity against *T. cruzi* amastigotes *and L. infantum* amastigotes, suggests the need to further investigate the intracellular behavior of these compounds. On the other hand, the 3-nitrotriazolyl amide 6e is significally less toxic against *T. cruzi* and *L. infantum* (higher SI). The spacer length between the 4-(1-adamantyl)phenyl backbone and the nitroheterocyclic ring plays a crucial role in the potency of these series of derivatives. The two more interesting adducts 2b and 6e share a two-atom distance between the phenyl ring and the carbonyl group (2C or, C, N). Tailored modifications are recommended to enhance their physicochemical properties, potentially enabling them to more efficiently target trypanosomatids having an intracellular stage in their life cycle. The results obtained against the intracellular *T. brucei* and *L. infantum* amastigotes highlight the need for structural modifications aiming at improving the physicochemical properties of these compounds—particularly parameters such as aqueous solubility and membrane permeability. These characteristics are especially important when targeting the trypanosome species which exhibit an intracellular stage in their life cycle. As such, potent trypanocidal derivatives must be capable of efficiently penetrating host cell membranes and reaching effective intracellular concentrations. Enhancing these properties will not only improve intracellular targeting but also contribute to more favourable pharmacokinetic profiles, including absorption, distribution, and overall bioavailability of future analogues.

## Biology

### Cytotoxic activity against rat skeletal myoblast L6††HeLa and L6 cells were obtained from the London School of Hygiene and Tropical Medicine (LSHTM) cell line repository. cells

The cytotoxicity against mammalian cells was assessed using microtitre plates. Briefly, L6 cells (a rat skeletal muscle line) were seeded at 1 × 10^4^ mL^−1^ in 200 μL of RPMI-1640 growth medium (with 10% fetal calf serum) containing 7 different compound concentrations in a range previously established to encompass both the IC_50_ and IC_90_ values. The plates were incubated for 5 days at 37 °C and 20 μL Alamar Blue (Biosource Ltd, Wilton, North Yorkshire England.) was then added to each well. After an additional 8 hours incubation, the fluorescence was determined using a Gemini fluorescent plate reader (Molecular Devices). Inhibition of growth was calculated by comparison with control values and IC_50_ and IC_90_ values were determined in triplicate using linear regression analysis.

### *Trypanosoma brucei* culturing and drug testing

Bloodstream form *T. brucei* (strain 427) were cultured at 37 °C in modified Iscove's medium. Trypanocidal activity was assessed by growing parasites in microtiter plates in the presence of various drug concentrations. Parasites were seeded at 0.25 × 10^5^ mL^−1^ in 200 μL of growth medium containing 7 different compound concentrations in a range previously established to encompass both the IC_50_ and IC_90_ values. The plates were incubated for 48 hours at 37 °C and 20 μL Alamar Blue was then added to each well. After an additional overnight incubation, the fluorescence was determined using a Gemini fluorescent plate reader (Molecular Devices). Inhibition of growth was calculated by comparison with control values and IC_50_ and IC_90_ values were determined in triplicate using linear regression analysis.

### *Trypanosoma cruzi* epimastigotes culturing and drug testing

*T. cruzi* epimastigotes (strain CL Brener) were cultured at 28 °C in supplemented RPMI-1640 medium (with 10% calf serum). Trypanocidal activity was assessed by growing parasites in microtiter plates in the presence of various drug concentrations. Parasites were seeded at 2.5 × 10^−5^ mL^−1^ in 200 μL of growth medium containing 7 different compound concentrations in a range previously established to encompass both the IC_50_ and IC_90_ values. The plates were incubated for 4 days at 28 °C and 20 μL Alamar Blue (Biosource UK Ltd) was then added to each well. After an additional 3 days incubation, the fluorescence was determined using a Gemini fluorescent plate reader (Molecular Devices). Inhibition of growth was calculated by comparison with control values and IC_50_ and IC_90_ values were determined in triplicate using linear regression analysis.

### Intracellular *Trypanosoma cruzi* amastigotes culturing and drug testing

Assays were performed in 96-well black (clear bottom) microtitre plates (Corning Inc.). HeLa† cells were seeded at 10^4^ per well, incubated for 3 days, and then infected with *T. cruzi* CL Brener PpyRE9h : mScarlet^25^ culture form trypomastigotes (1 : 1 multiplicity of infection). After 16 hours, extracellular parasites were removed by washing in growth medium (×3), test compounds were added, and the plates incubated for 5 days. Fluorescence intensities were then determined using a BMG FLUOstar Omega plate reader (ex 545 nm, em 590 nm), and the data analysed using GraphPad Prism 9.0 software. The values were expressed as IC_50_/_90_ ± SD and are the mean of triplicate experiments.

### *Leishmania infantum* promastigotes culturing and drug testing

Promastigotes from the *L. infantum* strain (MHOM/MA/67/ITMAP-263) were cultivated in 5 ml of Schneider's insect medium, which was supplemented with 10% heat-inactivated fetal bovine serum (FBS), 200 U ml^−1^ of penicillin/streptomycin, 6 μg ml^−1^ of phenol red, and 5 mM of HEPES. The cultures were maintained in an incubator at 27 °C and diluted to a concentration of 2 × 10^5^ cells per ml every 5 days. For the assays the parasites used were equivalent to late/log with two or three days of culture.

*Leishmania infantum* Luciferase-expressing *L. infantum* (strain MHOM/MA/67/ITMAP-263) axenic amastigotes were cultured in MAA/20 medium at 37 °C with 5% CO_2_, as described by Sereno *et al.* 1998.^26^ The parasites were maintained in 5 mL T25 ventilated flasks and subcultured every seven days at a concentration of 1 × 10^6^ cells per mL.

The THP-1 cell line, a human leukemia line (ATCC® TIB-202™), was cultured in RPMI-1640 medium supplemented with 10% heat-inactivated fetal bovine serum (FBS), 2 mM. l-Glutamine, 100 IU mL^−1^ penicillin/streptomycin, and 20 mM HEPES. The cells were maintained in a humidified incubator at 37 °C with 5% CO2. Subculturing was performed every three days, using 20 mL of media at a concentration of 2 × 10^5^ cells per mL in a T75 flask. All cell culture reagents were purchased from Lonza Bioscience (Morrisville, NC).

The efficacy of the compounds against *L. infantum* promastigotes was evaluated using a resazurin-based assay. Parasites were added to 100 μl of serial dilutions of the compounds in supplemented complete medium at a cell density of 5 × 10^5^ ml^−1^. As a quality control, a dose–response curve with the antileishmanial drug miltefosine was included in all assays. The final volume of each assay was 200 μl per well, and each condition was tested in duplicate. After a 72-hour incubation under specific conditions for each parasite, 20 μl of a 0.5 mM resazurin solution was added, and the plates were incubated for an additional 4 hours under the same conditions. Fluorescence was measured at 544 nm for excitation and 590 nm for emission using a Synergy 2 Multi-Mode Reader (Biotek, Winooski, VT, USA). Results were expressed as the percentage of parasite growth inhibition compared to controls (untreated parasites) and represent the average of at least three independent experiments. The effect was evaluated by determining the IC50 value (the concentration required to inhibit 50% growth), calculated using non-linear regression curves *via* GraphPad Prism version 8.1.1 for Windows (GraphPad Software, San Diego, CA, USA).

### *Leishmania infantum* amastigotes culturing and drug testing

The activity against intracellular amastigotes of *L. infantum* was evaluated using a modified method described by Santarem, Tavares, and Cordeiro-da-Silva (2019).^27^ THP-1 cells were differentiated into macrophages using PMA, as previously outlined. The differentiated macrophages were then infected with *L. infantum* axenic amastigotes expressing episomal luciferase at a macrophage-to-amastigote ratio of 1 : 10 for four hours at 37 °C in a 5% CO2 atmosphere. After this incubation, non-internalized parasites were washed away, and various concentrations of test compounds were added in a final volume of 100 μL. A dose–response curve for miltefosine was included in all assays to serve as a quality control. Each experimental condition was tested in quadruplicate. After 72 hours of incubation, the medium was replaced with 100 μL of PBS, and 25 μL of Glo-lysis buffer from the Steady-Glo Luciferase Assay System (Promega, Madison, WI, USA) was added. The plates were then agitated at 100 rpm for 10 minutes at room temperature. Following this, 30 μL of the Steady-Glo reagent (Promega, Madison, WI, USA) was added, and the mixture was incubated for 15 minutes in the dark under the same conditions. A total of 140 μL from each well was transferred to white-bottom 96-well plates, and luminescence intensity was measured using a Synergy 2 Multi-Mode Reader (Biotek, Winooski, VT, USA). The antileishmanial effect was assessed by comparing the results with those from non-treated infected cells. The IC_50_ value were determined through non-linear regression analysis using GraphPad Prism version 8.1.1 for Windows (GraphPad Software, San Diego, CA, USA). Results represent the average of at least three independent experiments.

The cytotoxicity of the compounds on THP-1-derived macrophages was evaluated using the colorimetric MTT assay (3-(4,5-dimethylthiazol-2-yl)-2,5-diphenyltetrazolium bromide), as described in Magoulas *et al.* (2021).^28^ THP-1 cells were suspended in complete RPMI medium at a density of 1 × 10^6^ cells per mL, and 100 μL of this suspension was seeded into each well of a 96-well plate. The cells were differentiated into macrophages by adding 40 ng mL^−1^ of phorbol-myristate 13-acetate (PMA, Sigma, Saint Louis, MI, USA) for 24 hours, followed by replacement with fresh medium for an additional 24 hours. Subsequently, the cells were incubated with 100 μL of various compounds, diluted in complete RPMI medium, at concentrations ranging from 100 to 12.5 μM. Each condition was tested in triplicate. After 72 hours of incubation at 37 °C with 5% CO2, the medium was removed, and 200 μL of a 0.5 mg mL^−1^ MTT solution, diluted in RPMI, was added to each well. The plates were incubated for an additional 4 hours. Following this incubation, 160 μL of the medium was removed and replaced with 160 μL of 2-propanol. Absorbance was measured at 570 nm using a Synergy 2 Multi-Mode Reader (Biotek, Winooski, VT, USA). Cytotoxicity was determined by presenting the CC50 (the concentration of the drug that reduces cell viability by 50%) interval or by calculating the CC_50_ value through non-linear regression analysis using GraphPad Prism version 8.1.1 for Windows (GraphPad Software, San Diego, CA, USA). The results represent the average of at least three independent experiments.

## Synthetic procedures

All chemicals and solvents were obtained from commercial suppliers and used without further purification. Concentrated refers to the removal of solvent with a rotary evaporator at normal water aspirator pressure, followed by further evacuation on a high-vacuum line. Reactions were monitored by thin layer chromatography. Thin-layer chromatography was performed using E. Merck precoated silica gel 60 F_254_ plates. Developed TLC plates were visualized with UV light (254 nm) and iodine. The chromatographic purification of the products was carried out using Silica gel 60 (40–63 μm, 230–400 mesh ASTM, Silica flash). Melting points were determined on a Büchi 530 apparatus and are uncorrected. Infrared (IR) spectra were recorded on a Perkin-Elmer 833 spectrophotomere.^1^H-NMR and ^13^C-NMR spectra were taken in CDCl_3_, DMSO-*d*_6_, methanol-*d*_4_ or acetone-*d*_6_ at 293 K (20 °C) on a Bruker Ultrashield™ Plus Avance III 600 spectrometer (150.9 MHz, ^13^C-NMR) and a Bruker DRX400 spectrometer (100.62 MHz, ^13^C-NMR). The measured chemical shifts are reported in *δ* (ppm), and the residual solvent signal was used as the internal calibration standard (CDCl_3_: ^1^H = 7.26 ppm, ^13^C = 77.18 ppm); (acetone-*d*_6_: ^1^H = 2.05 ppm, ^13^C = 29.84 ppm); (methanol-*d*_4_: ^1^H = 3.31 ppm, ^13^C = 49.00 ppm); (DMSO-*d*_6_: 1H = 2.50 ppm, ^13^C = 39.52 ppm). Splitting patterns are designated as follows: s, singlet; br s, broad singlet; d, doublet; t, triplet; q, quartet; multiplet; complex m, complex multiplet. Coupling constants (*J*) are expressed in units of Hertz (Hz). ^1^H- and ^13^C-NMR peaks were assigned based on the combined analysis of a series of ^1^H–^1^H (COSY) and ^1^H–^13^C (HSQC, HMBC) correlation spectra. Microanalyses for the final compounds were carried out by the NCSR Demokritos, Greece, and the results obtained had a maximum deviation of ± 0.4% from the theoretical value.

### 4-(2-Adamantyl)benzoyl azide (9)

A solution of sodium azide (500 mg, 8.08 mmol) in water (5 mL) was added dropwise, with stirring and cooling (∼0 °C), to a solution of chloride 8 (ref. 8) (860 mg, 3.12 mmol) in anhydrous acetone (25 mL). The reaction mixture was stirred at 0 °C for 0.5 hours, and then the acetone was removed under vacuum. Water was added to the residue, and the resulting mixture was extracted with benzene. The combined organic extracts were washed with water, dried over Na_2_SO_4_, and the solvent was evaporated *in vacuo* at a temperature below 40 °C. A pale yellow solid product (870 mg) was obtained and used in the next step without further purification (Yield: 99%) IR (Nujol), *v*(N=N): 2133.5 cm^−1^, *v*(C=O) 1701.2 cm^−1^.

### 4-(2-Adamantyl)aniline (10)

A solution of acyl azide 9 (870 mg, 3.11 mmol) in anhydrous toluene (3 mL) was gradually heated until gas evolution is observed (∼95 °C). Heating was carefully continued at 95 °C until the N_2_ gas evolution ceased. Then, concentrated HCl (5 mL) was added dropwise to the reaction mixture, causing vigorous gas evolution. Stirring continued at this temperature until CO_2_ evolution ceases (∼1 hour). The reaction mixture was then stirred until it reached room temperature, and the solvents were evaporated *in vacuo*. The residue was treated with anhydrous ether and left in the refrigerator for one day. The precipitated hydrochloride salt was collected by filtration and dried, to yield 440 mg of a white crystalline product (yield: 54%). M.p.: 278 °C (MeOH/Et_2_O) (dec); ^1^H-NMR (400 MHz, DMSO-*d*_6_), *δ* (ppm): 1.52–1.55 (d, 2H, *J* ≈ 12.4, 4,9-H_eq_), 1.66–1.73 (complex m, 5H, 4,9-H_ax_, 5,6-H), 1.94 (s, 5H, 7,8,10-H), 2.44 (s, 2H, 1,3-H), 2.97 (s, 1H, 2-H), 7.32–7.34 (d, 2H, *J* ≈ 8.4 Hz, 3,5-H_ar_), 7.44–7.46 (d, 2H, *J* ≈ 8.4 Hz, 2,6-H_ar_), 10.23 (s, 3H, NH_2_·HCl); ^13^C-NMR (100 MHz, DMSO-*d*_6_), *δ* (ppm): 26.97 (7-C), 27.30 (5-C), 30.21 (1,3-C), 31.41 (4,9-C), 37.21 (6-C), 38.22 (8,10-C), 45.68 (2-C), 122.93 (3,5-C_ar_), 127.88 (2,6-C_ar_), 129.02 (4-C_ar_), 143.66 (1-C_ar_). The free base was obtained from its hydrochloride salt by addition of a saturated Na_2_CO_3_ solution, followed by extraction with ethyl acetate using standard procedures.

### 4-(2-Adamantyl)benzamide (11)

A solution of chloride 8 (ref. 8) (540 mg, 1.95 mmol) in anhydrous tetrahydrofuran (5 mL) was added dropwise, with stirring and cooling, to an aqueous NH_3_ solution (25%) (15 mL). The reaction mixture was stirred at room temperature for 0.5 hours, and then tetrahydrofuran was removed *in vacuo*. Water is added to the residue, and the resulting mixture was extracted with ethyl acetate. The combined organic extracts were washed with water, dried over Na_2_SO_4_, and the solvent was evaporated *in vacuo*. A white crystalline product (344 mg) was obtained (yield: 71%).

**Fig. 2:**
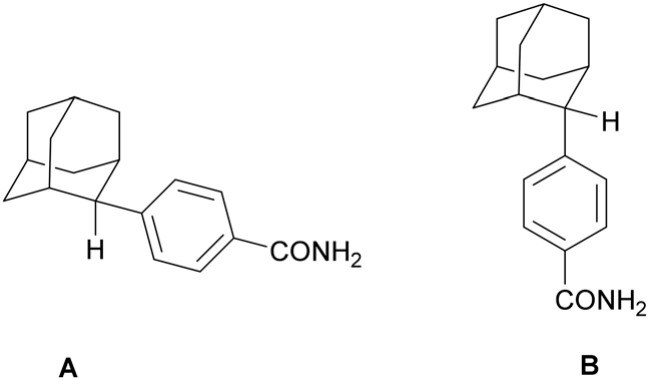
Structure of rotamers A and B.

The product is a mixture of rotamers A and B (Fig. 2).

^1^H-NMR (CDCl_3_), *δ* (ppm): (rotamer A): 1.56–1.59 (∼d, 2H, 4,9-H_eq_), 1.64–1.79 (complex m, 6H, 4,9-H_ax_, 5,6,7-H), 1.85–2.04 (complex m, 4H, 8,10-H), 2.49 (s, 1H, 1-H), 2.62–2.65 (∼d, 1H, 3-H), 2.88 (s, 1H, 2-H), 5.93 (br.s, 2H, NH_2_), 7.42–7.44 (d, 2H, *J* ≈ 8.7 Hz, 3,5-H_ar_), 7.76–7.78 (d, 2H, *J* ≈ 8.7 Hz, 2,6-H_ar_); ^13^C-NMR (100 MHz, DMSO-*d*_6_), *δ* (ppm): 26.79 (7-C), 27.81 (5-C), 30.21 (1-C), 32.12 (4,9-C), 33.84 (3-C), 35.12 (6-C), 39.19 (8,10-C), 47.11 (2-C), 127.28 (3,5-C_ar_), 127.42 (2,6-C_ar_), 130.32 (4-C_ar_), 149.32 (1-C_ar_), 169.71 (C=O).

^1^H-NMR (CDCl_3_), *δ* (ppm): (rotamer B): 1.56–1.59 (∼d, 2H, 4,9-H_eq_), 1.64–1.79 (complex m, 6H, 4,9-H_ax_, 5,6,7-H), 1.85–2.04 (complex m, 4H, 8,10-H), 2.49 (s, 1H, 1-H), 2.62–2.65 (∼d, 1H, 3-H), 3.03 (s, 1H, 2-H), 5.93 (br.s, 2H, NH_2_), 7.62–7.64 (d, 2H, *J* ≈ 8.7 Hz, 3,5-H_ar_), 7.82–7.84 (d, 2H, *J* ≈ 8.7 Hz, 2,6-H_ar_); ^13^C-NMR (100 MHz, DMSO-*d*_6_), *δ* (ppm): 27.19 (7-C), 28.07 (5-C), 31.19 (1-C), 32.12 (4,9-C), 33.84 (3-C), 37.30 (2-C), 37.87 (6-C), 39.19 (8,10-C), 125.59 (3,5-C_ar_), 128.03 (2,6-C_ar_), 132.43 (4-C_ar_), 148.35 (1-C_ar_), 169.71 (C=O).

### [4-(2-Adamantyl)phenyl]methan-1-amine (12)

Amide 11 (350 mg, 1.37 mmol) in anhydrous tetrahydrofuran (10 mL) was added dropwise to a stirred suspension of LiAlH_4_ (700 mg, 19.0 mmol) in anhydrous tetrahydrofuran (30 mL). The reaction mixture was gently refluxed for 24 hours and then was hydrolyzed at 0 °C by dropwise addition of ethanol, water and NaOH 10% solution. The inorganic hydroxides were removed by filtration and washed with hot tetrahydrofuran. The combined filtrates were evaporated *in vacuo* and water was added into the residue. The resulting mixture was extracted with AcOEt, the combined organic layers were washed with water, dried over Na_2_SO_4_ and evaporated *in vacuo*. The residue was dissolved in ethanol, and an ethanolic solution of 2.5 M HCl is added until pH ∼1. After refrigeration for 1 day, the precipitated hydrochloride salt was collected by filtration and dried. Yield: 250 mg (66%) of a white crystalline product. M.p.: 275 °C (MeOH/Et_2_O) (dec); ^1^H-NMR (400 MHz, DMSO-*d*_6_), *δ* (ppm): 1.51–1.54 (d, 2H, *J* ≈ 12, 4,9-H_eq_), 1.67–1.73 (complex m, 5H, 4,9-H_ax_, 5,6-H), 1.94 (∼s, 5H, 7,8,10-H), 2.46 (∼s, 2H, 1,3-H), 2.95 (s, 1H, 2-H), 3.96–3.97 (d, 2H, *J* ≈ 5.3 Hz, α-H), 7.37–7.39 (d, 2H, *J* ≈ 8.4 Hz, 3,5-H_ar_), 7.45–7.47 (d, 2H, *J* ≈ 8.4 Hz, 2,6-H_ar_), 8.49 (s, 3H, NH_2_·HCl); ^13^C-NMR (100 MHz, DMSO-*d*_6_), *δ* (ppm): 27.04 (7-C), 27.33 (5-C), 30.19 (1,3-C), 31.49 (4,9-C), 37.27 (6-C), 38.39 (8,10-C), 41.82 (α-C), 45.84 (2-C), 126.73 (3,5-C_ar_), 128.89 (2,6-C_ar_), 130.88 (4-C_ar_), 144.03 (1-C_ar_). The free base was obtained from its hydrochloride salt by addition of a saturated Na_2_CO_3_ solution, followed by extraction with ethyl acetate using standard procedures.

### 2-[4-(2-Adamantyl)phenyl]ethan-1-amine (14)

To a stirred solution of nitrile 13 (ref. 13) (200 mg, 0.8 mmol) in anhydrous tetrahydrofuran (15 mL), a 2 M borane dimethyl sulfide complex (BMS) in toluene (1.5 mL) was added dropwise under stirring and cooling. The solution was stirred at room temperature for 24 hours under an argon atmosphere. The next day, the reaction mixture was gently refluxed for 3 hours. The reaction mixture was stirred until it reached room temperature, methanol (∼20 mL) was added, and stirring continued for another 0.5 hours. The solvents were then removed under vacuum, and 2.5 M ethanolic HCl solution was added to the residue (until pH ∼ 1). After one day in the refrigerator, the precipitated hydrochloride salt was collected by filtration and dried over P_2_O_5_. Yield: 190 mg (81%) of a white crystalline product. M.p.: 216 °C (MeOH/Et_2_O) (dec); ^1^H-NMR (400 MHz, DMSO-*d*_6_), *δ* (ppm): 1.50–1.53 (d, 2H, *J* ≈ 13, 4,9-H_eq_), 1.70–1.73 (∼d, 5H, 4,9-H_ax_, 5,6-H), 1.89–1.97 (m, 5H, 7,8,10-H), 2.43 (∼s, 2H, 1,3-H), 2.84–2.88 (∼t, 2H, β-CH_2_), 2.92 (s, 1H, 2-H), 3.00–3.04 (∼t, 2H, α-H), 7.20–7.22 (d, 2H, *J* ≈ 8.2 Hz, 3,5-H_ar_), 7.29–7.31 (d, 2H, *J* ≈ 8.2 Hz, 2,6-H_ar_), 8.03 (s, 3H, NH_2_·HCl). The free base was obtained from its hydrochloride salt by addition of a saturated Na_2_CO_3_ solution, followed by extraction with ethyl acetate using standard procedure.

### General procedure for the preparation of carboxamides 2a–c & 7a as exemplified for compound 7a

To a stirred solution of the aniline hydrochloride 15 (ref. 14) (200 mg, 0.76 mmol) in a solvent mixture of anhydrous DMF : anhydrous DCM (1 : 1, 10 mL), the following reagents were sequentially added: 5-nitro-furoic acid (143 mg, 0.91 mmol), HBTU (345 mg, 0.91 mmol), and DIPEA (0.5 ml, 343 mg, 2.65 mmol). The mixture was stirred at room temperature for 24 hours under an argon atmosphere. The reaction mixture was then extracted with ethyl acetate, and the combined organic layers were washed with water, and brine, dried over Na_2_SO_4_, and evaporated *in vacuo*. The residue was purified by column chromatography using a gradient elution of methanol in ethyl acetate (0–5%) to afford 7a as a yellow-orange solid (210 mg, 76%).

#### *N*-[4-(2-Adamantyl)phenyl]-5-nitrofuran-2-carboxamide (2a)

Yield: 78% (from aniline 10); yellow-orange solid; M.p.: 222–224 °C (dec) (Et_2_O); ^1^H-NMR (400 MHz, CDCl_3_), *δ* (ppm): 1.56–1.60 (∼d, 2H, 4,9-H_eq_), 1.78–1.85 (m, 5H, 4,9-H_ax_, 5,6-H), 1.93–2.03 (m, 5H, 7,8,10-H), 2.47 (s, 2H, 1,3-H), 3.00 (s, 1H, 2-H), 7.37–7.41 (m, 4H, 3,4-H_f_, 3,5-H_ar_), 7.61–7.63 (d, 2H, *J* ≈ 8.7 Hz, 2,6-H_ar_), 8.18 (s, 1H, NH); ^13^C-NMR (100 MHz, CDCl_3_), *δ* (ppm): 27.87 (7-C), 28.17 (5-C), 31.20 (1,3-C), 32.07 (4,9-C), 37.99 (6-C), 39.23 (8,10-C), 46.68 (2-C), 112.86 (4-C_f_), 116.77 (3-C_f_), 120.38 (3,5-C_ar_), 127.83 (2,6-C_ar_), 133.64 (1-C_ar_), 142.20 (2-C_f_), 148.21 (4-C_ar_), 151.27 (5-C_f_), 154.00 (C=O). Anal. calcd for C_21_H_22_N_2_O_4_: C, 68.84; H, 6.05; N, 7.65; found: C, 68.51; H, 6.38; N, 7.83.

#### *N*-[4-(2-Adamantyl)benzyl]-5-nitrofuran-2-carboxamide (2b)

Yield: 70% (from methanamine 12); orange solid; M.p.: 153 °C (dec) (Et_2_O).

The product is a mixture of ***E*** and ***Z*** conformers*. E* > *Z*.

^1^H-NMR (400 MHz, CDCl_3_), *δ* (ppm): (conformer *E*): ^1^H-NMR (400 MHz, CDCl_3_), *δ* (ppm): 1.55–1.58 (d, 2H, *J* ≈ 12 Hz 4,9-H_eq_), 1.77–1.84 (m, 5H, 4,9-H_ax_, 5,6-H), 1.92–2.02 (m, 5H, 7,8,10-H), 2.46 (s, 2H, 1,3-H), 3.00 (s, 1H, 2-H), 4.60–4.62 (d, 2H, *J* ≈ 6 Hz, α-H), 6.83 (s, 1H, NH), 7.28–7.31 (m, 3H, 3-H_f_, 3,5-H_ar_), 7.35–7.37 (m, 3H, 4-H_f_, 2,6-H_ar_); ^13^C-NMR (100 MHz, CDCl_3_), *δ* (ppm): 27.88 (7-C), 28.15 (5-C), 31.20 (1,3-C), 32.09 (4,9-C), 37.98 (6-C), 39.25 (8,10-C), 43.54 (α-C), 46.80 (2-C), 112.57 (4-C_f_), 116.21 (3-C_f_), 127.59 (2,6-C_ar_), 128.16 (3,5-C_ar_), 133.73 (1-C_ar_), 144.66 (2-C_f_), 148.16 (4-C_ar_), 151.41 (5-C_f_), 156.20 (C=O).

^1^H-NMR (CDCl_3_), *δ* (ppm): (400 MHz) (conformer *Z*): ^1^H-NMR (400 MHz, CDCl_3_), *δ* (ppm): 1.55–1.58 (d, 2H, *J* ≈ 12 Hz 4,9-H_eq_), 1.77–1.84 (m, 5H, 4,9-H_ax_, 5,6-H), 1.92–2.02 (m, 5H, 7,8,10-H), 2.46 (s, 2H, 1,3-H), 3.00 (s, 1H, 2-H), 4.64–4.65 (∼d, 2H, α-H), 6.83 (s, 1H, NH), 7.28–7.31 (m, 1H, 3-H_f_), 7.35–7.37 (m, 1H, 4-H_f_), 7.46–7.48 (∼d, 2H, 3,5-H_ar_), 7.53–7.55 (∼d, 2H, 2,6-H_ar_); ^13^C-NMR (100 MHz, CDCl_3_), *δ* (ppm): 27.88 (7-C), 28.15 (5-C), 31.20 (1,3-C), 32.09 (4,9-C), 37.98 (6-C), 39.25 (8,10-C), 43.40 (α-C), 46.80 (2-C), 112.57 (4-C_f_), 116.21 (3-C_f_), 126.24 (2,6-C_ar_), 127.84 (3,5-C_ar_), 133.73 (1-C_ar_), 144.66 (2-C_f_), 148.16 (4-C_ar_), 151.41 (5-C_f_), 156.20 (C=O). Anal. calcd for C_22_H_24_N_2_O_4_: C, 69.46; H, 6.36; N, 7.36; found: C, 69.27; H, 6.49; N, 7.11.

#### *N*-[4-(2-Adamantyl)phenethyl]-5-nitrofuran-2-carboxamide (2c)

Yield: 76% (from ethanamine 14); yellow-orange solid; M.p.: 228 °C (dec) (Et_2_O).

^1^H-NMR (600 MHz, CDCl_3_), *δ* (ppm): 1.55–1.57 (d, 2H, *J* ≈ 13 Hz, 4,9-H_eq_), 1.77–1.85 (m, 5H, 4,9-H_ax_, 5,6-H), 1.93–2.02 (m, 5H, 7,8,10-H), 2.46 (s, 2H, 1,3-H), 2.91–2.93 (t, 2H, *J* ≈ 7.1 Hz, β-CH_2_), 2.99 (s, 1H, 2-H), 3.70–3.73 (d, 2H, *J* ≈ 6.8 Hz, α-H), 6.61 (s, 1H, NH), 7.28–7.31 (d, 2H, *J* ≈ 7.6 Hz, 3,5-H_ar_), 7.23–7.24 (d, 1H, *J* ≈ 4 Hz, 3-H_f_), 7.32–7.34 (m, 3H, 4-H_f_, 2,6-H_ar_); ^13^C-NMR (150 MHz, CDCl_3_), *δ* (ppm): 27.97 (7-C), 28.22 (5-C), 31.26 (1,3-C), 32.13 (4,9-C), 35.20 (β-C), 38.06 (6-C), 39.33 (8,10-C), 43.54 (α-C), 46.80 (2-C), 112.47 (4-C_f_), 115.88 (3-C_f_), 127.49 (3,5-C_ar_), 128.64 (2,6-C_ar_), 134.90 (1-C_ar_), 143.26 (2-C_f_), 148.32 (4-C_ar_), 151.36 (5-C_f_), 156.35 (C=O); anal. calcd for C_23_H_26_N_2_O_4_: C, 70.03; H, 6.64; N, 7.10; found: C, 70.17; H, 6.38; N, 6.99.

#### *N*-[4-(1-Adamantyl)phenyl]-5-nitrofuran-2-carboxamide (7a)

M.p.: 243–245 °C (dec) (Et_2_O). ^1^H-NMR (CDCl_3_), *δ* (ppm): (600 MHz, CDCl_3_), *δ* (ppm): 1.75–1.81 (∼q, 6H, 4,6,10-H), 1.92 (s, 6H, 2,8,9-H), 2.11 (s, 3H, 3,5,7-H), 7.36–7.40 (m, 4H, 3,4-H_f_, 3,5-H_ar_), 7.60–7.61 (d, 2H, *J* ≈ 8.7 Hz, 2,6-H_ar_), 8.17 (s, 1H, NH); ^13^C-NMR (150 MHz, CDCl_3_), *δ* (ppm): 29.09 (3,5,7-C), 36.24 (1-C), 36.91 (4,6,10-C), 43.34 (2,8,9-C), 112.80 (3-C_f_), 116.67 (4-C_f_), 120.37 (3,5-C_ar_), 125.89 (2,6-C_ar_), 133.90 (1-C_ar_), 148.23 (2-C_f_), 149.15 (4-C_ar_), 151.46 (5-C_f_), 154.00 (C=O); anal. calcd for C_21_H_22_N_2_O_4_: C, 68.84; H, 6.05; N, 7.65; found: C, 69.03; H, 6.23; N, 7.44.

### General procedure for the preparation of bromoacetamides 18–23 and 32 as exemplified for compound 18

To a solution of 4-(1-adamantyl)aniline (15) (300 mg, 1.32 mmol) in anhydrous chloroform (5 mL), water (5 mL) and Na_2_CO_3_ (154 mg, 1.45 mmol) were added. Then, under stirring and an argon atmosphere, a solution of bromoacetyl chloride (229 mg, 1.45 mmol) in anhydrous chloroform (2 mL) was added dropwise at 0 °C. The reaction mixture was stirred at room temperature under an argon atmosphere for 48 hours. After completion of the reaction, the organic layer was separated, washed with a Na_2_CO_3_ solution, brine, and water, dried over Na_2_SO_4_, and evaporated *in vacuo* to afford the crude bromoacetamide 18 which was used in the next step without further purification.

#### *N*-[4-(1-Adamantyl)phenyl]-2-bromoacetamide (18)

400 mg (from aniline 15 (ref. 14)); sticky brown-orange solid.

#### *N*-[4-(1-Adamantyl)benzyl]-2-bromoacetamide (19)

500 mg (from methanamine 16 (ref. 15)); sticky brown-orange solid.

#### *N*-[4-(1-Adamantyl)phenethyl]-2-bromoacetamide (20)

200 mg (from ethanamine 17 (ref. 15)); sticky brown-orange solid.

#### *N*-[4-(2-Adamantyl)phenyl]-2-bromoacetamide (21)

180 mg (from aniline 10); sticky brown-orange solid.

#### *N*-[4-(2-Adamantyl)benzyl]-2-bromoacetamide (22)

260 mg (from methanamine 12); sticky brown-orange solid.

#### *N*-[4-(2-Adamantyl)phenethyl]-2-bromoacetamide (23)

200 mg (from ethanamine 14); sticky brown-orange solid.

#### *N*-[2-(1-Adamantyl)phenyl]-2-bromoacetamide (32)

388 mg (from aniline 31 (ref. 19)); sticky brown-orange solid.

### General procedure for the preparation of 2-nitroimidazole acetamides 3a–c & 4a–c as exemplified for compound 3a

To a stirred solution of 2-nitroimidazole (515 mg, 4.55 mmol) in anhydrous dimethylformamide (15 mL), sodium hydride (NaH) (131, mg, 5.46 mmol, 218 mg of a 60% dispersion in mineral oil, pre-washed with *n*-pentane) was added in small portions. The reaction mixture was heated at 70 °C under an argon atmosphere for 1 hour. Then, a solution of bromoacetamide 18 in anhydrous DMF (5 mL) was added, and the reaction mixture was stirred at 80 °C for 48 hours under an argon atmosphere. After completion, the reaction mixture was cooled, water was added, and extraction was performed with ethyl acetate. The combined organic extracts were washed with brine and water, dried over Na_2_SO_4_, and evaporated *in vacuo*. The residue was purified by column chromatography, using a gradient elution of methanol in ethyl acetate (5–20%) to afford 3a as a dark brown-orange solid (176 mg, 35% from 15).

#### *N*-[4-(1-Adamantyl)phenyl]-2-(2-nitro-1*H*-imidazol-1-yl)acetamide (3a)

M.p.: 106–107 °C (dec) (AcOEt/*n*-hexane); ^1^H-NMR (400 MHz, acetone-*d*_6_), *δ* (ppm): 1.78–1.81 (∼q, 6H, 4,6,10-H), 1.91 (br.s, 6H, 2,8,9-H), 2.09 (s, 3H, 3,5,7-H), 4.02 (s, 2H, α′-CH_2_), 7.23 (br.s, 1H, NH), 7.32–7.34 (d, 2H, *J* ≈ 8.7 Hz, 3,5-H_ar_), 7.39–7.41 (∼d, 1H, 4-H_im_), 7.52–7.66 (complex m, 3H, 5-H_im_, 2,6-H_ar_); ^13^C-NMR (100 MHz, acetone-*d*_6_), *δ* (ppm): 29.45 (3,5,7-C), 31.89 (α-C), 36.54 (1-C), 37.38 (4,6,10-C), 43.89 (2,8,9-C), 119.99 (3,5-C_ar_), 120.16 (5-C_im_), 125.94 (2,6-C_ar_), 126.10 (4-C_im_), 137.28 (1-C_ar_), 147.40 (2-C_im_), 147.91 (4-C_ar_), 165.12 (C=O); anal. calcd for C_21_H_24_N_4_O_3_: C, 66.30; H, 6.36; N, 14.73; found: C, 66.46; H, 6.09; N, 14.49.

#### *N*-[4-(1-Adamantyl)benzyl]-2-(2-nitro-1*H*-imidazol-1-yl)acetamide (3b)

Yield: 38% (from 16); dark brown-orange solid; M.p.: 117–118 °C (dec) (AcOEt/*n*-hexane).

The product is a mixture of ***E*** and ***Z*** conformers. *E*/*Z*: 1.8.

^1^H-NMR (400 MHz, CDCl_3_), *δ* (ppm): (conformer *E*): 1.73–1.81 (∼q, 6H, 4,6,10-H), 1.89 (br.s, 6H, 2,8,9-H), 2.10 (s, 3H, 3,5,7-H), 4.42–4.44 (d, 2H, *J* ≈ 5.6 Hz, α-CH_2_), 5.00 (s, 2H, α′-CH_2_), 6.22 (br.s, 1H, NH), 7.13–7.16 (m, 2H, 2,6-H_ar_), 7.20–7.22 (d, *J* ≈ 8.2 Hz, 4-H_im_), 7.25–7.30 (m, 2H, 3,5-H_ar_), 7.33–7.35 (d, *J* ≈ 8.2 Hz, 5-H_im_); ^13^C-NMR (100 MHz, CDCl_3_), *δ* (ppm): 29.02 (3,5,7-C), 36.38 (1-C), 36.85 (4,6,10-C), 43.99 (2,8,9-C), 44.58 (α-C), 52.43 (α′-C), 124.72 (3,5-C_ar_), 125.57 (5-C_im_), 127.31 (4-C_im_), 128.71 (2,6-C_ar_), 136.81 (1-C_ar_), 144.84 (2-C_im_), 152.41 (4-C_ar_), 164.59 (C=O).

^1^H-NMR (400 MHz, CDCl_3_), *δ* (ppm): (conformer *E*): 1.73–1.81 (∼q, 6H, 4,6,10-H), 1.89 (br.s, 6H, 2,8,9-H), 2.10 (s, 3H, 3,5,7-H), 4.42–4.47 (m, 2H, α-CH_2_), 5.00–5.01 (∼d, 2H, α′-CH_2_), 6.22 (br.s, 1H, NH), 7.06–7.08 (∼d, 1H, 4-H_im_), 7.13–7.16 (m, 2H, 3,5-H_ar_), 7.20–7.35 (very complex m, 3H, 5-H_im_, 2,6-H_ar_); ^13^C-NMR (100 MHz, CDCl_3_), *δ* (ppm): 29.02 (3,5,7-C), 36.38 (1-C), 36.85 (4,6,10-C), 43.99 (2,8,9-C), 43.94 (α-C), 52.37 (α′-C), 124.72 (2,6-C_ar_), 125.19 (5-C_im_), 127.86 (4-C_im_), 128.78 (2,6-C_ar_), 134.18 (1-C_ar_), 144.84 (2-C_im_), 151.34 (4-C_ar_), 164.59 (C=O). Anal. calcd for C_22_H_26_N_4_O_3_: C, 66.99; H, 6.64; N, 14.20; found: C, 67.16; H, 6.97; N, 14.09.

#### *N*-[4-(1-Adamantyl)phenethyl]-2-(2-nitro-1*H*-imidazol-1-yl) acetamide (3c)

Yield: 33% (from 17); dark brown-orange solid; M.p.: 125–126 °C (dec) (AcOEt/*n*-hexane).

The product is a mixture of ***E*** and ***Z*** conformers*. E*/*Z*: 1.6.

^1^H-NMR (400 MHz, CDCl_3_), *δ* (ppm): (conformer *E*): 1.73–1.81 (∼q, 6H, 4,6,10-H), 1.90 (br.s, 6H, 2,8,9-H), 2.09 (s, 3H, 3,5,7-H), 2.80–2.85 (m, 2H, β-CH_2_), 2.93 (s, 2H, α′-CH_2_), 3.51–3.57 (m, 2H, α-CH_2_), 7.02–7.04 (d, 1H, *J* ≈ 6.6 Hz, 4-H_im_), 7.14–7.31 (very complex m, 6H, NH, 5-H_im_, 3,5-H_ar_, 2,6-H_ar_); ^13^C-NMR (100 MHz, CDCl_3_), *δ* (ppm): 29.07 (3,5,7-C), 35.33 (1-C), 36.18 (β-C), 36.92 (4,6,10-C), 40.31 (α-C), 43.34 (2,8,9-C), 63.25 (α′-C), 123.13 (3,5-C_ar_), 125.14 (5-C_im_), 125.57 (4-C_im_), 128.60 (2,6-C_ar_), 138.73 (1-C_ar_), 149.72 (2-C_im_), 151.84 (4-C_ar_), 170.57 (C=O).

^1^H-NMR (400 MHz, CDCl_3_), *δ* (ppm): (conformer *Z*): 1.73–1.81 (∼q, 6H, 4,6,10-H), 1.90 (br.s, 6H, 2,8,9-H), 2.09 (s, 3H, 3,5,7-H), 2.80–2.85 (m, 2H, β-CH_2_), 2.92 (s, 2H, α′-CH_2_), 3.51–3.57 (m, 2H, α-CH_2_), 7.02–7.04 (d, 1H, *J* ≈ 6.6 Hz, 4-H_im_), 7.14–7.31 (very complex m, 6H, NH, 5-H_im_, 3,5-H_ar_, 2,6-H_ar_); ^13^C-NMR (100 MHz, CDCl_3_), *δ* (ppm): 29.07 (3,5,7-C), 35.33 (1-C), 36.05 (β-C), 36.92 (4,6,10-C), 40.16 (α-C), 43.34 (2,8,9-C), 63.19 (α′-C), 123.13 (3,5-C_ar_), 125.14 (5-C_im_), 125.97 (4-C_im_), 128.47 (2,6-C_ar_), 136.03 (1-C_ar_), 149.72 (2-C_im_), 151.84 (4-C_ar_), 170.57 (C=O). Anal. calcd for C_23_H_28_N_4_O_3_: C, 67.63; H, 6.91; N, 13.72; found: C, 67.36; H, 7.13; N, 13.44.

#### *N*-[4-(2-Adamantyl)phenyl]-2-(2-nitro-1*H*-imidazol-1-yl)acetamide (4a)

Yield: 23% (from 10); dark brown-orange solid; M.p.: 130–131 °C (dec) (AcOEt/*n*-hexane); ^1^H-NMR (400 MHz, CDCl_3_), *δ* (ppm): 1.55–1.97 (very complex m, 12H, 4,5,6,7,8,9,10-H), 2.37–2.43 (∼d, 2H, 1,3-H), 2.69 (br.s, 1H, 2-H), 3.03 (s, 1H, α′-CH_2_), 3.36 (s, 1H, α′-CH_2_), 4.95 (br.s, 1H, NH), 7.44–7.55 (very complex m, 5H, 3,5-H_ar_, 2,6-H_ar_, 4-H_im_, 5-H_im_), 7.43–7.52 (m, 1H, 4-H_im_); ^13^C-NMR (100 MHz, CDCl_3_), *δ* (ppm): 27.89 (7-C), 29.84 (5-C), 31.16 (1,3-C), 32.04 (4,9-C), 33.00 (α′-C), 38.02 (6-C), 39.23 (8,10-C), 46.13 (2-C), 119.48 (3,5-C_ar_), 120.18 (5-C_im_), 126.23 (4-C_im_), 127.47 (2,6-C_ar_), 136.09 (1-C_ar_), 140.97 (2-C_im_), 153.12 (4-C_ar_), 172.28 (C=O); anal. calcd for C_21_H_24_N_4_O_3_: C, 66.30; H, 6.36; N, 14.73; found: C, 66.38; H, 6.21; N, 14.66.

#### *N*-[4-(2-Adamantyl)benzyl]-2-(2-nitro-1*H*-imidazol-1-yl)acetamide (4b)

Yield: 26% (from 12); dark brown-orange solid; M.p.: 119–121 °C (dec) (AcOEt/*n*-hexane); ^1^H-NMR (400 MHz, CDCl_3_), *δ* (ppm): 1.54–1.57 (∼d, 2H, 4,9-H_eq_), 1.61–1.77 (m, 5H, 4,9-H_ax_, 5,6-H), 1.92–2.01 (m, 5H, 7,8,10-H), 2.44 (br.s, 2H, 1,3-H), 2.97 (br.s, 1H, 2-H), 3.40 (s, 2H, α-H), 4.47 (s, 2H, α′-CH_2_), 6.80 (br.s, 1H, NH), 7.08–7.40 (very complex m, 5H, 4-H_im_, 2,3,5,6-H_ar_), 7.43–7.52 (m, 1H, 5-H_im_); ^13^C-NMR (100 MHz, CDCl_3_), *δ* (ppm): 27.88 (7-C), 28.15 (5-C), 29.85 (1,3-C), 31.18 (4,9-C), 32.08 (α-C), 37.79 (6-C), 39.25 (8,10-C), 42.73 (α′-C), 46.74 (2-C), 119.22 (3,5-C_ar_), 124.60 (5-C_im_), 125.16 (4-C_im_), 127.70 (2,6-C_ar_), 134.61 (1-C_ar_), 144.06 (2-C_im_), 155.52 (4-C_ar_), 173.51 (C=O); anal. calcd for C_22_H_26_N_4_O_3_: C, 66.99; H, 6.64; N, 14.20; found: C, 66.75; H, 6.72; N, 14.47.

#### *N*-[4-(2-Adamantyl)phenethyl]-2-(2-nitro-1*H*-imidazol-1-yl)acetamide (4c)

Yield: 31% (from 14); dark brown-orange solid; M.p.: 110–111 °C (dec) (AcOEt/*n*-hexane);^1^H-NMR (400 MHz, acetone-*d*_6_/methanol-*d*_4_), *δ* (ppm): 1.57–1.58 (∼d, 2H, 4,9-H_eq_), 1.75–1.81 (∼d, 5H, 4,9-H_ax_, 5,6-H), 1.96–2.03 (m, 5H, 7,8,10-H), 2.45 (br.s, 2H, 1,3-H), 2.79 (br.s, 1H, 2-H), 2.90–3.03 (d, 2H, *J* ≈ 6.8 Hz, β-H), 3.32 (s, 2H, α′-CH_2_), 3.44–3.45 (very complex m, 2H, α-CH_2_), 7.14–7.20 (m, 3H, NH, 3,5-H_ar_), 7.28–7.35 (very complex m, 3H, 4-H_im_, 2,6-H_ar_), 7.43–7.52 (m, 1H, 5-H_im_); ^13^C-NMR (100 MHz, CDCl_3_), *δ* (ppm): 27.68 (7-C), 28.42 (5-C), 31.61 (1,3-C), 32.17 (4,9-C), 35.00 (β-C), 38.16 (6-C), 39.38 (α-C), 39.42 (8,10-C), 46.99 (2-C), 47.21 (α′-C), 125.87 (3,5-C_ar_), 127.15 (5-C_im_), 127.84 (4-C_im_), 128.59 (2,6-C_ar_), 136.31 (1-C_ar_), 143.23 (2-C_im_), 152.25 (4-C_ar_), 174.51 (C=O); anal. calcd for C_23_H_28_N_4_O_3_: C, 67.63; H, 6.91; N, 13.72; found: C, 67.67; H, 6.84; N, 13.91.

### General procedure for the preparation of carboxamides 5a,b as exemplified for 5a

A solution of 2-[4-(1-adamantyl)phenyl]acetyl chloride 14 (ref. 14) (326 mg, 1.13 mmol) in anhydrous dichloromethane (2 mL) was added dropwise, under stirring and at 0 °C, to a solution of amine 24 (ref. 16 and 17) (180 mg, 1.15 mmol) in anhydrous dichloromethane (3.0 mL) and Et_3_N (582 mg, 5.75 mmol, 0.8 ml). The reaction mixture was stirred at room temperature for 24 hours under an argon atmosphere. Upon completion of the reaction, water was added, and the mixture was extracted with dichloromethane. The combined organic extracts were washed with brine and water, dried over Na_2_SO_4_, and the solvent was evaporated *in vacuo*. The residue was purified by column chromatography using gradient elution with a methanol/ethyl acetate solvent mixture, from 5 to 20% to afford 5a as a yellow solid (286 mg, 62%).

#### 2-[4-(1-Adamantyl)phenyl]-*N*-[2-(2-nitro-1*H*-imidazol-1-yl)ethyl] acetamide (5a)

M.p.: 140–142 °C (AcOEt/*n*-hexane).

The product is a mixture of *E* and *Z* conformers. *E*/*Z*: 10/3.

^1^H-NMR (CDCl_3_), *δ* (ppm): (conformer *E*) (400 MHz, CDCl_3_), *δ* (ppm): 1.65–1.74 (q, 6H, *J* ≈ 11 Hz 4,6,10-H), 1.82 (br.s, 6H, 2,8,9-H), 2.03 (s, 3H, 3,5,7-H), 3.49 (s, 2H, α-CH_2_), 3.56–3.59 (∼d, 2H, α′-CH_2_), 4.44–4.47 (t, 2H, *J* ≈ 5.6 Hz, β′-CH_2_), 6.04 (br.s, 1H, NH), 6.70–6.71 (∼d, 1H, 5-H_im_), 6.87–6.89 (∼d, 1H, 4-H_im_), 7.19–7.27 (m, 4H, 2,3,5,6-H_ar_); ^13^C-NMR (100 MHz, CDCl_3_), *δ* (ppm): 29.00 (3,5,7-C), 36.36 (1-C), 36.83 (4,6,10-C), 39.83 (α′-C), 43.30 (2,8,9-C), 44.06 (α-C), 49.14 (β′-C), 124.29 (3,5-C_ar_), 125.78 (2,6-C_ar_), 127.26 (5-C_im_), 128.23 (4-C_im_), 134.29 (1-C_ar_), 144.57 (2-C_im_), 152.57 (4-C_ar_), 172.48 (C=O).

^1^H-NMR (CDCl_3_), *δ* (ppm): (conformer *Z*) (400 MHz, CDCl_3_), *δ* (ppm): 1.65–1.74 (q, 6H, *J* ≈ 11 Hz 4,6,10-H), 1.82 (br.s, 6H, 2,8,9-H), 2.03 (s, 3H, 3,5,7-H), 3.46 (s, 2H, α-CH_2_), 3.56–3.59 (∼d, 2H, α′-CH_2_), 4.44–4.47 (t, 2H, *J* ≈ 5.6 Hz, β′-CH_2_), 6.04 (br.s, 1H, NH), 6.70–6.71 (∼d, 1H, 5-H_im_), 6.87–6.89 (∼d, 1H, 4-H_im_), 6.96–6.98 (∼d, 2H, 3,5- H_ar_), 7.08–7.11 (∼d, 2H, 2,6-H_ar_); ^13^C-NMR (100 MHz, CDCl_3_), *δ* (ppm): 29.01 (3,5,7-C), 36.20 (1-C), 36.84 (4,6,10-C), 39.83 (α′-C), 43.30 (2,8,9-C), 44.06 (α-C), 49.14 (β′-C), 126.51 (3,5-C_ar_), 127.26 (5-C_im_), 128.23 (4-C_im_), 129.15 (2,6-C_ar_), 131.55 (1-C_ar_), 144.57 (2-C_im_), 150.95 (4-C_ar_), 172.48 (C=O). Anal. calcd for C_23_H_28_N_4_O_3_: C, 67.63; H, 6.91; N, 13.72; found: C, 67.58; H, 7.13; N, 13.58.

#### 2-[4-(1-Adamantyl)phenyl]-*N*-[3-(2-nitro-1*H*-imidazol-1-yl)propyl] acetamide (5b)

Yield: 58% (from 25 (ref. 16 and 17)); yellow solif; M.p.: 112–114 °C (AcOEt/*n*-hexane).

The product is a mixture of ***E*** and ***Z*** conformers. *E*/*Z*: 3/1.

^1^H-NMR (CDCl_3_), *δ* (ppm): (conformer *E*) (400 MHz, CDCl_3_), *δ* (ppm): 1.72–1.81 (∼q, 6H, 4,6,10-H), 1.90 (br.s, 6H, 2,8,9-H), 1.96–1.99 (m, 2H, β′-CH_2_), 2.09 (s, 3H, 3,5,7-H), 3.25–3.30 (q, 2H, *J* ≈ 6.2 Hz, α′-CH_2_), 3.59 (s, 2H, α-CH_2_), 4.33–4.36 (t, 2H, *J* ≈ 6.7 Hz, γ′-CH_2_), 5.61 (br.s, 1H, NH), 7.11 (s, 1H, 5-H_im_), 7.17–7.21 (m, 2H, 3,5-H_ar_), 7.25–7.26 (∼d, 1H, 4-H_im_), 7.31–7.33 (m, 2H, 2,6-H_ar_); ^13^C-NMR (100 MHz, CDCl_3_), *δ* (ppm): 29.01 (3,5,7-C), 31.16 (β′-C), 36.22 (1-C), 36.40 (α′-C), 36.84 (4,6,10-C), 43.30 (2,8,9-C), 44.32 (α-C), 47.77 (γ′-C), 124.39 (2,6-C_ar_), 125.93 (3,5-C_ar_), 126.26 (4-C_im_), 128.64 (5-C_im_), 134.34 (1-C_ar_), 144.27 (2-C_im_), 152.72 (4-C_ar_), 172.21 (C=O).

^1^H-NMR (CDCl_3_), *δ* (ppm): (conformer *E*) (400 MHz, CDCl_3_), *δ* (ppm): 1.72–1.81 (∼q, 6H, 4,6,10-H), 1.90 (br.s, 6H, 2,8,9-H), 1.96–1.99 (m, 2H, β′-CH_2_), 2.09 (s, 3H, 3,5,7-H), 3.25–3.30 (q, 2H, *J* ≈ 6.2 Hz, α′-CH_2_), 3.56 (s, 2H, α-CH_2_), 4.33–4.36 (t, 2H, *J* ≈ 6.7 Hz, γ′-CH_2_), 5.61 (br.s, 1H, NH), 7.08–7.11 (m, 2H, 3.5-H_ar_),7.11 (s, 1H, 5-H_im_), 7.25–7.26 (∼d, 1H, 4-H_im_), 7.35–7.37 (m, 2H, 2,6-H_ar_); ^13^C-NMR (100 MHz, CDCl_3_), *δ* (ppm): 29.01 (3,5,7-C), 31.16 (β′-C), 36.22 (1-C), 36.40 (α′-C), 36.84 (4,6,10-C), 43.30 (2,8,9-C), 44.32 (α-C), 47.77 (γ′-C), 126.26 (4-C_im_), 126.61 (3,5-C_ar_), 128.64 (5-C_im_), 129.32 (2,6-C_ar_), 134.34 (1-C_ar_), 144.27 (2-C_im_), 152.72 (4-C_ar_), 172.21 (C=O). Anal. calcd for C_24_H_30_N_4_O_3_: C, 68.22; H, 7.16; N, 13.26; found: C, 68.16; H, 7.44; N, 13.45.

### 2-[2-(3-Nitro-1*H*-1,2,4-triazol-1-yl)ethyl]isoindoline-1,3-dione (27)

To a stirred solution of 3-nitro-1*H*-1,2,4-triazole (500 mg, 4.40 mmol) in anhydrous dimethylformamide (15 mL), sodium hydride (NaH) (116 mg, 4.80 mmol, 195 mg of a 60% dispersion in mineral oil, pre-washed with *n*-pentane) was added. The reaction mixture was stirred at room temperature under an argon atmosphere for 1.5 hours. Then, *N*-(3-bromoethyl)phthalimide (1.20 g, 4.70 mmol) was added, and the reaction mixture was stirred at room temperature under an argon atmosphere for 5 days. Water was then added, and the mixture was extracted with ethyl acetate. The combined organic extracts were washed with brine and water, dried over Na_2_SO_4_, and the solvent was evaporated *in vacuo*, to afford 1.3 g of a white solid, which was used in the next step without further purification.

### 2-(3-Nitro-1*H*-1,2,4-triazol-1-yl)than-1-amine hydrochloride (29)

To a stirred suspension of dione 27 (1.30 g, 4.50 mmol) in ethanol (30 mL), hydrazine hydrate (0.82 mL, 453 mg, 9.10 mmol) was added, and the mixture was stirred at room temperature for 2.5 hours. After completion of the reaction, the mixture was cooled to 0 °C, filtered, and the filtrate was evaporated *in vacuo*. The residue was dissolved in ethanol, and a 2.5 M ethanolic HCl solution was added until pH ∼ 1. After refrigerating for one day, the precipitated hydrochloride salt was collected by filtration, dried, and recrystallized from a methanol/ethyl acetate mixture, to afford 700 mg of a pale yellow solid (yield: 80%). All data are in accordance to literature.^7^

### 2-[3-(3-Nitro-1*H*-1,2,4-triazol-1-yl)propyl]isoindoline-1,3-dione (28)

Prepared from 3-nitro-1*H*-1,2,4-triazole and *N*-(3-bromopropyl)phthalimide in an analogous manner to dione 27. This afforded 1.60 g of a white solid, which was used in the next step without further purification.

### 3-(3-Nitro-1*H*-1,2,4-triazol-1-yl)propan-1-amine hydrochloride (30)

Prepared by hydrazinolysis of dione 28 in an analogous manner to hydrochloride 29. This afforded 590 mg of a pale yellow solid (yield: 76%). M.p.: 195–197 °C (dec) (MeOH/AcOEt). ^1^H-NMR (DMSO-*d*_6_), *δ* (ppm): 2.12–2.19 (quintet, 2H, *J* ≈ 7.1 Hz, β-CH_2_), 2.78–2.86 (quintet, 2H, *J* ≈ 7.2 Hz, α-CH_2_), 4.45–4.48 (t, 2H, *J* ≈ 6.5 Hz, γ-CH_2_), 8.11 (br.s, 3H, NH_2_·HCl), 8.94 (s, 1H, 5-H_t_).

### General procedure for the preparation of carboxamides 6a–f as exemplified for compound 6a

To a stirred solution of the ethanamine hydrochloride 29 (200 mg, 1 mmol) in a solvent mixture of anhydrous DMF : anhydrous DCM (1 : 1, 10 mL), the following reagents were sequentially added: 4-(1-adamantyl)-benzoic acid^19^ (318 mg, 1.2 mmol), HBTU (470 mg, 1.2 mmol), and DIPEA (467 mg, 3.7 mmol, 0.6 ml). The mixture was stirred at room temperature for 24 hours under an argon atmosphere. The reaction mixture was then extracted with ethyl acetate, and the combined organic layers were washed with water and brine, dried over Na_2_SO_4_, and evaporated *in vacuo*. The residue was purified by column chromatography using a gradient elution of methanol in ethyl acetate (0–20%) to afford benzamide 6a as a white crystalline solid. (290 mg, 71%).

#### 4-(1-Adamantyl)-*N*-[2-(3-nitro-1*H*-1,2,4-triazol-1-yl)ethyl] benzamide (6a)

M.p.: 150–152 °C (AcOEt/*n*-hexane).

The product is a mixture of ***E*** and ***Z*** conformers. *E*/*Z*: 9/1.

^1^H-NMR (CDCl_3_), *δ* (ppm): (conformer *E*) (600 MHz, CDCl_3_), *δ* (ppm):1.73–1.81 (q, 6H, *J* ≈ 21.6 Hz 4,6,10-H), 1.90 (br.s, 6H, 2,8,9-H), 2.10 (s, 3H, 3,5,7-H), 3.92–3.95 (q, 2H, *J* ≈ 5.5 Hz, α′-CH_2_), 4.58–4.59 (t, 2H, *J* ≈ 5.5 Hz, β′-CH_2_), 6.67 (br.s, 1H, NH), 7.39–7.41 (d, 2H, *J* ≈ 8.1 Hz, 3,5-H_ar_), 7.65–7.66 (d, 2H, *J* ≈ 8.1 Hz, 2,6-H_ar_), 8.15 (s, 1H, 5-H_t_); ^13^C-NMR (150 MHz, CDCl_3_), *δ* (ppm): 28.96 (3,5,7-C), 31.71 (1-C), 36.80 (4,6,10-C), 39.69 (α′-C), 43.10 (2,8,9-C), 50.44 (β′-C), 125.52 (3,5-C_ar_), 126.97 (2,6-C_ar_), 130.41 (1-C_ar_), 145.91 (5-C_t_), 156.11 (4-C_ar_), 163.27 (3-C_t_), 168.55 (CO).

^1^H-NMR (CDCl_3_), *δ* (ppm): (conformer *Z*) (600 MHz, CDCl_3_), *δ* (ppm): 1.73–1.81 (q, 6H, *J* ≈ 21.6 Hz 4,6,10-H), 1.90 (br.s, 6H, 2,8,9-H), 2.10 (s, 3H, 3,5,7-H), 3.92–3.95 (q, 2H, *J* ≈ 5.5 Hz, α′-CH_2_), 4.58–4.59 (t, 2H, *J* ≈ 5.5 Hz, β′-CH_2_), 6.72 (br.s, 1H, NH), 7.39–7.41 (d, 2H, *J* ≈ 8.1 Hz, 3,5-H_ar_), 7.65–7.66 (d, 2H, *J* ≈ 8.1 Hz, 2,6-H_ar_), 8.17 (s, 1H, 5-H_t_); ^13^C-NMR (150 MHz, CDCl_3_), *δ* (ppm): 28.96 (3,5,7-C), 31.71 (1-C), 36.80 (4,6,10-C), 39.69 (α′-C), 43.10 (2,8,9-C), 50.44 (β′-C), 125.52 (3,5-C_ar_), 126.97 (2,6-C_ar_), 130.41 (1-C_ar_), 145.91 (5-C_t_), 156.11 (4-C_ar_), 163.27 (3-C_t_), 168.55 (C=O). Anal. calcd for C_21_H_25_N_5_O_3_: C, 63.78; H, 6.37; N, 17.71; found: C, 63.66; H, 6.59; N, 17.63.

#### 2-[4-(1-Adamantyl)phenyl]-*N*-[2-(3-nitro-1*H*-1,2,4-triazol-1-yl) ethyl]acetamide (6b)

Yield: 64% (from 29 and 2-[4-(1-adamantyl)phenyl]acetic acid^14^); white crystalline solid; M.p.: 150–151 °C (AcOEt/*n*-hexane).

The product is a mixture of ***E*** and ***Z*** conformers. *E*/*Z*: 4/1.

^1^H-NMR (CDCl_3_), *δ* (ppm): (conformer *E*) (400 MHz, CDCl_3_), *δ* (ppm): 1.72–1.81 (m, 6H, 4,6,10-H), 1.88 (br.s, 6H, 2,8,9-H), 2.09 (s, 3H, 3,5,7-H), 3.50 (s, 2H, α-CH_2_), 3.67–3.71 (q, 2H, *J* ≈ 5.5 Hz, α′-CH_2_), 4.41–4.44 (t, 2H, *J* ≈ 5.7 Hz, β′-CH_2_), 5.67 (br.s, 1H, NH), 7.07–7.09 (d, 2H, *J* ≈ 8.2 Hz, 3,5-H_ar_), 7.30–7.32 (d, 2H, *J* ≈ 8.2 Hz, 2,6-H_ar_), 7.98 (s, 1H, 5-H_t_); ^13^C-NMR (100 MHz, CDCl_3_), *δ* (ppm): 29.01 (3,5,7-C), 36.20 (1-C), 36.84 (4,6,10-C), 39.12 (α′-C), 43.20 (2,8,9-C), 43.26 (α-C), 49.99 (β′-C), 125.97 (3,5-C_ar_), 129.07 (2,6-C_ar_), 131.06 (1-C_ar_), 145.70 (5-C_t_), 151.30 (4-C_ar_), 163.15 (3-C_t_), 172.63 (CO).

^1^H-NMR (CDCl_3_), *δ* (ppm): (conformer *Z*) (400 MHz, CDCl_3_), *δ* (ppm): 1.72–1.81 (m, 6H, 4,6,10-H), 1.88 (br.s, 6H, 2,8,9-H), 2.09 (s, 3H, 3,5,7-H), 3.53 (s, 2H, α-CH_2_), 3.67–3.71 (q, 2H, *J* ≈ 5.5 Hz, α′-CH_2_), 4.41–4.44 (t, 2H, *J* ≈ 5.7 Hz, β′-CH_2_), 5.67 (br.s, 1H, NH), 7.07–7.09 (d, 2H, *J* ≈ 8.2 Hz, 3,5-H_ar_), 7.30–7.32 (d, 2H, *J* ≈ 8.2 Hz, 2,6-H_ar_), 7.92 (s, 1H, 5-H_t_); ^13^C-NMR (100 MHz, CDCl_3_), *δ* (ppm): 29.01 (3,5,7-C), 36.20 (1-C), 36.84 (4,6,10-C), 39.12 (α′-C), 43.20 (2,8,9-C), 43.26 (α-C), 49.99 (β′-C), 125.97 (3,5-C_ar_), 129.07 (2,6-C_ar_), 131.06 (1-C_ar_), 145.70 (5-C_t_), 151.30 (4-C_ar_), 163.15 (3-C_t_), 172.63 (C=O). Anal. calcd for C_22_H_27_N_5_O_3_: C, 64.53; H, 6.65; N, 17.10; found: C, 64.72; H, 6.34; N, 17.04.

#### 3-[4-(1-Adamantyl)phenyl]-*N*-[2-(3-nitro-1*H*-1,2,4-triazol-1-yl) ethyl]propanamide (6c)

Yield: 76% (from 29 and 3-[4-(1-adamantyl)phenyl]propanoic acid^14^); white crystalline solid; M.p.: 89–90 °C (AcOEt/*n*-hexane).

The product is a mixture of ***E*** and ***Z*** conformers. *E*/*Z*: 6/1.

^1^H-NMR (400 MHz, CDCl_3_), *δ* (ppm): (conformer *E*) 1.71–1.80 (q, 6H, *J* ≈ 8.47 Hz, 4,6,10-H), 1.88 (br.s, 6H, 2,8,9-H), 2.08 (br.s, 3H, 3,5,7-H), 2.46–2.49 (t, 2H, *J* ≈ 7.4 Hz, α-CH_2_), 2.86–2.95 (quintet, 2H, *J* ≈ 7.2 Hz, β-CH_2_), 3.64–3.69 (q, 2H, *J* ≈ 5.3 Hz, α′-CH_2_), 4.31–4.34 (t, 2H, *J* ≈ 5.5 Hz, β′-CH_2_), 5.85–5.88 (∼t, 1H, NH), 7.12–7.14 (d, 2H, *J* ≈ 7.12 Hz, 3,5-H_ar_), 7.28–7.30 (d, 2H, *J* ≈ 7.12 Hz, 2,6-H_ar_), 7.65 (s, 1H, 5-H_t_); ^13^C-NMR (100 MHz, CDCl_3_), *δ* (ppm): 29.05 (3,5,7-C), 30.98 (β-C), 36.09 (1-C), 36.87 (4,6,10-C), 37.96 (α-C), 39.16 (α′-C), 43.32 (2,8,9-C), 43.36 (β′-C), 125.28 (3,5-C_ar_), 128.31 (2,6-C_ar_), 137.39 (1-C_ar_), 145.74 (5-C_t_), 149.96 (4-C_ar_), 163.15 (3-C_t_), 173.33 (C=O).

^1^H-NMR (400 MHz, CDCl_3_), *δ* (ppm): (conformer *Z*) 1.71–1.80 (q, 6H, *J* ≈ 8.47 Hz, 4,6,10-H), 1.88 (br.s, 6H, 2,8,9-H), 2.08 (br.s, 3H, 3,5,7-H), 2.46–2.49 (t, 2H, *J* ≈ 7.4 Hz, α-CH_2_), 2.86–2.95 (quintet, 2H, *J* ≈ 7.2 Hz, β-CH_2_), 3.53–3.61 (m, 2H, α′-CH_2_), 3.97–4.01 (m, 2H, β′-CH_2_), 5.85–5.88 (∼t, 1H, NH), 77.12–7.14 (d, 2H, *J* ≈ 7.12 Hz, 3,5-H_ar_), 7.28–7.30 (d, 2H, *J* ≈ 7.12 Hz, 2,6-H_ar_), 7.65 (s, 1H, 5-H_t_); ^13^C-NMR (100 MHz, CDCl_3_), *δ* (ppm): 29.05 (3,5,7-C), 30.98 (β-C), 36.09 (1-C), 36.87 (4,6,10-C), 37.96 (α-C) 39.16 (α′-C), 43.32 (2,8,9-C), 43.36 (β′-C), 125.17 (3,5-C_ar_), 128.20 (2,6-C_ar_), 137.39 (1-C_ar_), 145.74 (5-C_t_), 149.96 (4-C_ar_), 163.15 (3-C_t_), 173.60 (C=O). Anal. calcd for C_23_H_29_N_5_O_3_: C, 65.23; H, 6.90; N, 16.54; found: C, 65.51; H, 7.02; N, 16.75.

#### 4-(1-Adamantyl)-*N*-[3-(3-nitro-1*H*-1,2,4-triazol-1-yl)propyl] benzamide (6d)

Yield: 68% (from 30 and 4-(1-adamantyl)benzoic acid^19^); white crystalline solid; M.p.: 178–180 °C (AcOEt/*n*-hexane).

The product is a mixture of ***E*** and ***Z*** conformers. *E* > *Z.*

^1^H-NMR (CDCl_3_), *δ* (ppm): (conformer *E*) (400 MHz, CDCl_3_), *δ* (ppm): 1.73–1.82 (q, 6H, *J* ≈ 10.98 Hz, 4,6,10-H), 1.91 (br.s, 6H, 2,8,9-H), 2.11 (br.s, 3H, 3,5,7-H), 2.22–2.29 (quintet, 2H, *J* ≈ 6.1 Hz, β′-CH_2_), 3.50–3.54 (q, 2H, *J* ≈ 6 Hz, α′-CH_2_), 4.37–4.40 (t, 2H, *J* ≈ 6.3 Hz, γ′-CH_2_), 6.51 (br.s, 1H, NH), 7.42–7.44 (d, 2H, *J* ≈ 8.3 Hz, 3,5-H_ar_), 7.70–7.72 (d, 2H, *J* ≈ 8.3 Hz, 2,6-H_ar_), 8.47 (s, 1H, 5-H_t_); ^13^C-NMR (100 MHz, CDCl_3_), *δ* (ppm): 28.94 (3,5,7-C), 30.62 (β′-C), 31.72 (1-C), 36.69 (α′-C), 36.79 (4,6,10-C), 43.09 (2,8,9-C), 49.15 (γ′-C), 125.42 (3,5-C_ar_), 126.97 (2,6-C_ar_), 130.93 (1-C_ar_), 145.98 (5-C_t_), 155.88 (4-C_ar_), 163.04 (3-C_t_), 168.40 (CO).

^1^H-NMR (CDCl_3_), *δ* (ppm): (conformer *Z*) (400 MHz, CDCl_3_), *δ* (ppm): 1.73–1.82 (q, 6H, *J* ≈ 10.98 Hz, 4,6,10-H), 1.91 (br.s, 6H, 2,8,9-H), 2.11 (br.s, 3H, 3,5,7-H), 2.22–2.29 (quintet, 2H, *J* ≈ 6.1 Hz, β′-CH_2_), 3.62–3.68 (m, 2H, α′-CH_2_), 4.0.12–4.16 (m, 2H, γ′-CH_2_), 6.51 (br.s, 1H, NH), 7.52–7.54 (d, 2H, *J* ≈ 8.1 Hz, 3,5-H_ar_), 7.85–7.88 (d, 2H, *J* ≈ 8.1 Hz, 2,6-H_ar_), 8.50 (s, 1H, 5-H_t_); ^13^C-NMR (100 MHz, CDCl_3_), *δ* (ppm): 28.94 (3,5,7-C), 30.62 (β′-C), 31.72 (1-C), 36.69 (α′-C), 36.79 (4,6,10-C), 43.09 (2,8,9-C), 49.15 (γ′-C), 125.42 (3,5-C_ar_), 126.97 (2,6-C_ar_), 130.93 (1-C_ar_), 145.98 (5-C_t_), 155.88 (4-C_ar_), 163.04 (3-C_t_), 168.40 (C=O). Anal. calcd for C_22_H_27_N_5_O_3_: C, 64.53; H, 6.65; N, 17.10; found: C, 64.81; H, 6.31; N, 17.13.

#### 2-[4-(1-Adamantyl)phenyl]-*N*-[3-(3-nitro-1*H*-1,2,4-triazol-1-yl) propyl]acetamide (6e)

Yield: 72% (from 30 and 2-[4-(1-adamantyl)phenyl]acetic acid^14^); white crystalline solid; M.p.: 181–182 °C (AcOEt/*n*-hexane).

The product is a mixture of ***E*** and ***Z*** conformers. *E*/*Z*: 4/1.

^1^H-NMR (CDCl_3_), *δ* (ppm): (conformer *E*) (600 MHz, CDCl_3_), *δ* (ppm): 1.73–1.80 (q, 6H, *J* ≈ 20.9 Hz, 4,6,10-H), 1.88 (br.s, 6H, 2,8,9-H), 2.08 (br.m, 5H, 3,5,7-H, β′-CH_2_), 3.23–3.26 (q, 2H, *J* ≈ 6.4 Hz, α′-CH_2_), 3.53 (s, 2H, *J* ≈ 21 Hz, α-CH_2_), 4.24–4.26 (t, 2H, *J* ≈ 6.9 Hz, γ′-CH_2_), 5.79 (br.s, 1H, NH), 7.16–7.18 (d, 2H, *J* ≈ 8.1 Hz, 3,5-H_ar_), 7.33–7.34 (d, 2H, *J* ≈ 8.1 Hz, 2,6-H_ar_), 8.35 (s, 1H, 5-H_t_); ^13^C-NMR (100 MHz, CDCl_3_), *δ* (ppm): 28.99 (3,5,7-C), 30.23 (β′-C), 31.68 (1-C), 36.18 (α′-C), 36.82 (4,6,10-C), 43.26 (2,8,9-C), 43.36 (α-C), 48.94 (γ′-C), 125.85 (3,5-C_ar_), 129.20 (2,6-C_ar_), 131.50 (1-C_ar_), 145.89 (5-C_t_), 150.99 (4-C_ar_), 162.94 (3-C_t_), 172.51 (C=O).

^1^H-NMR (CDCl_3_), *δ* (ppm): (conformer *Z*) (600 MHz, CDCl_3_), *δ* (ppm): 1.73–1.80 (q, 6H, *J* ≈ 20.9 Hz, 4,6,10-H), 1.88 (br.s, 6H, 2,8,9-H), 2.08 (br.m, 5H, 3,5,7-H, β′-CH_2_), 3.23–3.26 (q, 2H, *J* ≈ 6.4 Hz, α′-CH_2_), 3.57 (s, 2H, *J* ≈ 21 Hz, α-CH_2_), 4.24–4.26 (t, 2H, *J* ≈ 6.9 Hz, γ′-CH_2_), 5.79 (br.s, 1H, NH), 7.23 (br.s, 2H, 3,5-H_ar_), 7.29–7.30 (m, 2H, 2,6-H_ar_), 8.33 (s, 1H, 5-H_t_); ^13^C-NMR (100 MHz, CDCl_3_), *δ* (ppm): 28.99 (3,5,7-C), 30.23 (β′-C), 31.68 (1-C), 36.18 (α′-C), 36.82 (4,6,10-C), 43.26 (2,8,9-C), 44.19 (α-C), 48.94 (γ′-C), 124.34 (3,5-C_ar_), 126.52 (2,6-C_ar_), 134.31 (1-C_ar_), 145.89 (5-C_t_), 152.66 (4-C_ar_), 162.94 (3-C_t_), 172.51 (C=O). Anal. calcd for C_23_H_29_N_5_O_3_: C, 65.23; H, 6.90; N, 16.54; found: C, 65.47; H, 7.09; N, 16.71.

#### 3-[4-(1-Adamantyl)phenyl]-*N*-[3-(3-nitro-1*H*-1,2,4-triazol-1-yl) propyl]propanamide (6f)

Yield: 76% (from 30 and 3-[4-(1-adamantyl)phenyl]propanoic acid^14^); white crystalline solid; M.p.: 65–67 °C (AcOEt/*n*-hexane).

The product is a mixture of ***E*** and ***Z*** conformers. *E*/*Z*: 13/1.

^1^H-NMR (400 MHz, CDCl_3_), *δ* (ppm): (conformer *E*) 1.69–1.76 (q, 6H, *J* ≈ 13.9 Hz 4,6,10-H), 1.84 (br.s, 6H, 2,8,9-H), 1.97–2.00 (quintet, 2H, *J* ≈ 6.1 Hz, β′-CH_2_), 2.06 (br.s, 3H, 3,5,7-H), 2.50–2.54 (t, 2H, *J* ≈ 7.6 Hz, α-CH_2_), 2.93–2.97 (t, 2H, *J* ≈ 7.6 Hz, β-CH_2_), 3.18–3.23 (q, 2H, *J* ≈ 6.1 Hz, α′-CH_2_), 4.02–4.05 (t, 2H, *J* ≈ 6.3 Hz, γ′-CH_2_), 5.76–5.79 (∼t, 1H, NH), 7.15–7.17 (d, 2H, *J* ≈ 7.12 Hz, 3,5-H_ar_), 7.26–7.28 (d, 2H, *J* ≈ 7.12 Hz, 2,6-H_ar_), 8.33 (s, 1H, 5-H_t_); ^13^C-NMR (100 MHz, CDCl_3_), *δ* (ppm): 29.00 (3,5,7-C), 30.31 (β′-C), 31.10 (β-C), 35.68 (α′-C), 36.04 (1-C), 36.83 (4,6,10-C), 38.23 (α-C), 43.35 (2,8,9-C), 48.51 (γ′-C), 125.19 (3,5-C_ar_), 128.24 (2,6-C_ar_), 137.50 (1-C_ar_), 146.03 (5-C_t_), 149.85 (4-C_ar_), 162.96 (3-C_t_), 173.22 (C=O).

^1^H-NMR (400 MHz, CDCl_3_), *δ* (ppm): (conformer *Z*) 1.69–1.76 (q, 6H, *J* ≈ 13.9 Hz 4,6,10-H), 1.86 (br.s, 6H, 2,8,9-H), 2.03 (br.s, 2H, β′-CH_2_), 2.06 (br.s, 3H, 3,5,7-H), 2.43–2.47 (m, 2H, *J* ≈ 7.6 Hz, α-CH_2_), 2.93–2.97 (t, 2H, *J* ≈ 7.6 Hz, β-CH_2_), 3.79–3.84 (m, 2H, α′-CH_2_), 4.10–4.12 (m, 2H, γ′-CH_2_), 5.76–5.79 (∼t, 1H, NH), 7.15–7.17 (d, 2H, *J* ≈ 7.12 Hz, 3,5-H_ar_), 7.26–7.28 (d, 2H, *J* ≈ 7.12 Hz, 2,6-H_ar_), 8.45 (s, 1H, 5-H_t_); ^13^C-NMR (100 MHz, CDCl_3_), *δ* (ppm): 29.04 (3,5,7-C), 30.31 (β′-C), 31.10 (β-C), 35.68 (α′-C), 36.04 (1-C), 36.88 (4,6,10-C), 38.23 (α-C), 43.35 (2,8,9-C), 48.51 (γ′-C), 125.05 (3,5-C_ar_), 128.19 (2,6-C_ar_), 137.50 (1-C_ar_), 146.03 (5-C_t_), 149.85 (4-C_ar_), 162.96 (3-C_t_), 172.25 (C=O). Anal. calcd for C_24_H_31_N_5_O_3_: C, 65.88; H, 7.14; N, 16.01; found: C, 65.69; H, 7.18; N, 16.17.

The ^1^H-NMR and ^13^C-NMR spectra of 6a–f in CDCl_3_ revealed the presence of mixtures of *E*- and *Z*-isomers due to the restricted rotation around the amide bond. The conformational studies are provided in the supporting information.

### General procedure for the preparation of carboxamides 7b,7c as exemplified for compound b

To a stirred solution of 3-nitro-1*H*-1,2,4-triazole (157 mg, 1.38 mmol) in anhydrous dimethylformamide (15 mL), sodium hydride (NaH) (37 mg, 1.52 mmol, 62 mg of a 60% dispersion in mineral oil, pre-washed with *n*-pentane) was added in small portions. The reaction mixture was heated at room temperature under an argon atmosphere for 0.5 hour. Subsequently, a solution of bromoacetamide 18 in anhydrous DMF (5 mL) was added, and the reaction mixture was stirred at 80 °C for 72 hours under an argon atmosphere. After completion, the reaction mixture was cooled, water was added, and extraction was performed with ethyl acetate. The combined organic extracts were washed with brine and water, dried over Na_2_SO_4_, and evaporated *in vacuo*. The residue was purified by column chromatography, using a gradient elution of with ethyl acetate in hexane (50–70%) to afford 7b as an off-yellow solid (122 mg, 91%).

#### *N*-[4-(1-Adamantyl)phenyl]-2-(3-nitro-1*H*-1,2,4-triazol-1-yl)acetamide (7b)

M.p.: >270 °C (AcOEt/*n*-hexane); ^1^H-NMR (400 MHz, DMSO-*d*_6_) *δ* (ppm): 1.75–1.82 (∼q, 6H, 4,6,10-H), 1.90 (br.s, 6H, 2,8,9-H), 2.10 (s, 3H, 3,5,7-H), 5.38 (s, 2H, α-CH_2_), 7.36–7.39 (d, 2H J ≈ 8.6 Hz, 3,5-H_ar_), 7.56–7.54 (d, 2H, *J* ≈ 8.6 Hz, 2,6-H_ar_), 8.96 (s, 1H, 5-H_t_), 10.49 (s, 1H, NH); ^13^C-NMR (100 MHz, DMSO-*d*_6_) *δ* (ppm): 28.24 (3,5,7-C), 35.23 (1-C), 37.00 (4,6,10-C), 42.82 (2,8,9-C), 53.23 (α-C), 119.14 (3,5-C_ar_), 125.09 (2,6-C_ar_), 136.22 (4-C_ar_), 146.82 (1-C_ar_), 148.31 (5-Ct), 161.97 (3-C_t_), 163.13 (C=O); anal. calcd for C_20_H_23_N_5_O_3_: C, 62.98; H, 6.08; N, 18.36; found: C, 62.76; H, 6.22; N, 18.31.

#### *N*-[2-(1-Adamantyl)phenyl]-2-(3-nitro-1*H*-1,2,4-triazol-1-yl)acetamide (7c)

The residue was purified by column chromatography, using a gradient elution of with methanol in ethyl acetate (5–20%) to afford 7c as a peachy solid (218 mg, 52% from bromoacetamide 32). M.p.: 201–203 °C (AcOEt/*n*-hexane); ^1^H-NMR (600 MHz, CDCl_3_) *δ* (ppm): 1.57 (s, 6H), 1.81 (s, 1H), 1.99 (s, 9H), 4.48 (s, 2H), 7.17 (t, *J* = 6 Hz, 2H), 7.42 (d, *J* = 6 Hz, 2H), 8.20 (s, 1H); ^13^C-NMR (150 MHz, CDCl_3_) *δ* (ppm): 30.22, 36.26, 40.92, 54.52, 129.58, 130.01, 130.26, 138.62, 146.88, 163.22; anal. calcd for C_20_H_23_N_5_O_3_: C, 62.98; H, 6.08; N, 18.36; found: C, 63.09; H, 6.11; N, 18.14.

## Conflicts of interest

There are no conflicts to declare.

## Supplementary Material

MD-017-D5MD00527B-s001

## Data Availability

The data supporting this article have been included into the SI and the experimental part of the manuscript. Supplementary information (SI) is available. See DOI: https://doi.org/10.1039/d5md00527b.

## References

[cit1] Parthasarathy A., Kalesh K. (2020). RSC Med. Chem..

[cit2] Abbasi Shiran J., Kaboudin B., Panahi N., Razzaghi-Asl N. (2024). Eur. J. Med. Chem..

[cit3] Field M. C., Horn D., Fairlamb A. H., Ferguson M. A. J., Gray D. W., Read K. D., De Rycker M., Torrie L. S., Wyatt P. G., Wyllie S., Gilbert I. H. (2017). Nat. Rev. Microbiol..

[cit4] Lindner A. K., Lejon V., Barrett M. P., Blumberg L., Bukachi S. A., Chancey R. J., Edielu A., Matemba L., Mesha T., Mwanakasale V., Pasi C., Phiri T., Seixas J., Akl E. A., Probyn K., Villanueva G., Simarro P. P., Kadima Ebeja A., Franco J. R., Priotto G. (2025). Lancet Infect. Dis..

[cit5] Wilkinson S. R., Taylor M. C., Horn D., Kelly J. M., Cheeseman I. (2008). Proc. Natl. Acad. Sci. U. S. A..

[cit6] Wyllie S., Patterson S., Stojanovski L., Simeons F. R. C., Norval S., Kime R., Read K. D., Fairlamb A. H. (2012). Sci. Transl. Med..

[cit7] Patterson S., Wyllie S. (2014). Trends Parasitol..

[cit8] Foscolos A.-S., Papanastasiou I., Tsotinis A., Taylor M. C., Kelly J. M. (2019). ChemMedChem.

[cit9] Ang C. W., Jarrad A. M., Cooper M. A., Blaskovich M. A. T. (2017). J. Med. Chem..

[cit10] Papadopoulou M. V., Bloomer W. D., Rosenzweig H. S., O'Shea I. P., Wilkinson S. R., Kaiser M., Chatelain E., Ioset J.-R. (2015). Bioorg. Med. Chem..

[cit11] Papadopoulou M. V., Bloomer W. D., Rosenzweig H. S., Chatelain E., Kaiser M., Wilkinson S. R., McKenzie C., Ioset J.-R. (2012). J. Med. Chem..

[cit12] Hwang H., Joo S., Kim S. (2015). Bull. Korean Chem. Soc..

[cit13] Papanastasiou I., Riganas S., Foscolos G. B., Tsotinis A., Akhtar S., Khan M., Rahman K., Thursto D. (2015). Lett. Org. Chem..

[cit14] Koperniku A., Foscolos A.-S., Papanastasiou I., Foscolos G. B., Tsotinis A., Schols D. (2016). Lett. Org. Chem..

[cit15] Chyi Tseng C., Handa I., Abdel-Sayed A. N., Bauer L. (1988). Tetrahedron.

[cit16] Hay M. P., Wilson W. R., Moselen J. W., Palmer B. D., Denny W. A. (1994). J. Med. Chem..

[cit17] Papadopoulou M. V., Bloomer W. D. (1993). Drugs Future.

[cit18] Hay M. P., Lee H. H., Wilson W. R., Roberts P. B., Denny W. A. (1995). J. Med. Chem..

[cit19] Georgiadis M.-O., Kourbeli V., Papanastasiou I. P., Tsotinis A., Taylor M. C., Kelly J. M. (2020). RSC Med. Chem..

[cit20] Dawson M. I., Xia Z., Jiang T., Ye M., Fontana J. A., Farhana L., Patel B., Xue L. P., Bhuiyan M., Pellicciari R., Macchiarulo A., Nuti R., Zhang X.-K., Han Y.-H., Tautz L., Hobbs P. D., Jong L., Waleh N., Chao W., Feng G.-S., Pang Y., Su Y. (2008). J. Med. Chem..

[cit21] Di Pietro O., Vicente-García E., Taylor M. C., Berenguer D., Viayna E., Lanzoni A., Sola I., Sayago H., Riera C., Fisa R., Clos M. V., Pérez B., Kelly J. M., Lavilla R., Muñoz-Torrero D. (2015). Eur. J. Med. Chem..

[cit22] Fersing C., Boudot C., Pedron J., Hutter S., Primas N., Castera-Ducros C., Bourgeade-Delmas S., Sournia-Saquet A., Moreau A., Cohen A., Stigliani J.-L., Pratviel G., Crozet M. D., Wyllie S., Fairlamb A., Valentin A., Rathelot P., Azas N., Courtioux B., Verhaeghe P., Vanelle P. (2018). Eur. J. Med. Chem..

[cit23] Campos M. C. O., Leon L. L., Taylor M. C., Kelly J. M. (2014). Mol. Biochem. Parasitol..

[cit24] Foscolos A.-S., Papanastasiou I., Foscolos G. B., Tsotinis A., Kellici T. F., Mavromoustakos T., Taylor M. C., Kelly J. M. (2016). MedChemComm.

[cit25] Olmo F., Jayawardhana S., Khan A. A., Langston H. C., Francisco A. F., Atherton R. L., Ward A. I., Taylor M. C., Kelly J. M., Lewis M. D. (2024). PLoS Neglected Trop. Dis..

[cit26] Sereno D., Cavaleyra M., Zemzoumi K., Maquaire S., Ouaissi A., Lemesre J. L. (1998). Antimicrob. Agents Chemother..

[cit27] Santarém N. , Tavares J. and Cordeiro-da-Silva A., in Leishmania, ed. J. Clos, Humana Press, New York, NY, 2019, vol. 1971, pp. 265–277 10.1007/978-1-4939-9210-2_14 30980309

[cit28] Magoulas G. E., Afroudakis P., Georgikopoulou K., Roussaki M., Borsari C., Fotopoulou T., Santarem N., Barrias E., Tejera Nevado P., Hachenberg J., Bifeld E., Ellinger B., Kuzikov M., Fragiadaki I., Scoulica E., Clos J., Gul S., Costi M. P., de Souza W., Prousis K. C., Cordeiro da Silva A., Calogeropoulou T. (2021). Molecules.

