# Supplementary material for: Synthesis and evaluation of nitroheterocyclic aromatic adamantane amides with trypanocidal activity. Part II
Source: RSC Med Chem. 2025 Nov 6;17(1):590–605. doi: 10.1039/d5md00527b (PMC12679539; doi:10.1039/d5md00527b)
Supplement: MD-017-D5MD00527B-s001 [file MD-017-D5MD00527B-s001.pdf]

Supplementary Information (SI) for RSC Medicinal Chemistry.
This journal is © The Royal Society of Chemistry 2025

Supporting Information

Synthesis and Evaluation of Nitroheterocyclic Aromatic Adamantane Amides with Trypanocidal Activity. Part II

Angeliki-Sofia Foscolos,[a,b] Richard L. Atherton,[c] Maria Billia,[d] Markos-Orestis Georgiadis,[a,e] Nuno Santarém,[f] Anabela Cordeiro da Silva,[f,g] Martin C. Taylor,[c] John M. Kelly,[c] Theodora Calogeropoulou,[h] Andrew Tsotinis,[a] Thomas Mavromoustakos[d] and Ioannis P. Papanastasiou[a]*

[a]School of Health Sciences, Department of Pharmacy, Division of Pharmaceutical Chemistry, National and Kapodistrian University of Athens, Panepistimioupoli-
Zografou, 157 71 Athens (Greece)
[b]Institute of Nanoscience & Nanotechnology, NCSR "Demokritos", 15341 Athens (Greece)
[c]Department of Infection Biology, London School of Hygiene and Tropical Medicine, London WC1E7HT (United Kingdom)
[d] Faculty of Chemistry, Department of Organic Chemistry, NKUA, 15771 Athens (Greece)
[e]Center for Drug Discovery and Department of Pharmaceutical Sciences, Northeastern University, Boston, MA 02115 (United States)
[f]Host-Parasite Interaction Group, i3S, Institute for Research and Innovation in Health, University of Porto, Porto 4200-135, (Portugal)
[g]Laboratory of Microbiology, Department of Biological Sciences, Faculty of Pharmacy, University of Porto, 4050-313, Porto, (Portugal)
[h]Institute of Chemical Biology, National Hellenic Research Foundation, 11635 Athens (Greece)

* Corresponding author: *papanastasiou@pharm.uoa.gr*

Table of Contents

Chemistry.....2

Reduction protocols evaluated for the synthesis of amine 14.....2

Conformational Studies.....3

In silico assessment of the compounds' drug-likeness.....6

In silico toxicity assessment of the compounds.....8

Heat map of antiparasitic activity.....16

^{1}H and ^{13}C NMR spectra.....17

References.....36

Chemistry

All chemicals and solvents were obtained from commercial suppliers and used without further purification. Concentrated refers to the removal of solvent with a rotary evaporator at normal water aspirator pressure, followed by further evacuation on a high-vacuum line. Reactions were monitored by thin layer chromatography. Thin-layer chromatography was performed using E. Merck precoated silica gel 60 F_{254} plates. Developed TLC plates were visualized with UV light (254 nm) and iodine. The chromatographic purification of the products was carried out using Silica gel 60 (40–63 µm, 230–400 mesh ASTM, Silica flash). Melting points were determined on a Büchi 530 apparatus and are uncorrected. ^{1}H-NMR and ^{13}C-NMR spectra were taken in DMSO-d_6 at 293 K (20 °C) and recorded on a Bruker Ultrashiel™ Plus Avance III 600 spectrometer (150.9 MHz, ^{13}C-NMR) and a Bruker DRX400 spectrometer (100.62 MHz, ^{13}C-NMR). The measured chemical shifts are reported in δ (ppm), and the residual solvent signal was used as the internal calibration standard (DMSO-d$_6$: 1H = 2.50 ppm, ^{13}C = 39.52 ppm). Splitting patterns are designated as follows: s, singlet; br s, broad singlet; d, doublet; t, triplet; q, quartet; multiplet; complex m, complex multiplet. Coupling constants (*J*) are expressed in units of Hertz (Hz). ^{1}H- and ^{13}C-NMR peaks were assigned based on the combined analysis of a series of ^{1}H-^{1}H (COSY) and ^{1}H-^{13}C (HSQC, HMBC) correlation spectra.

Reduction protocols evaluated for the synthesis of amine 14

2-[4-(2-Adamantyl)phenyl]ethan-1-amine (14)

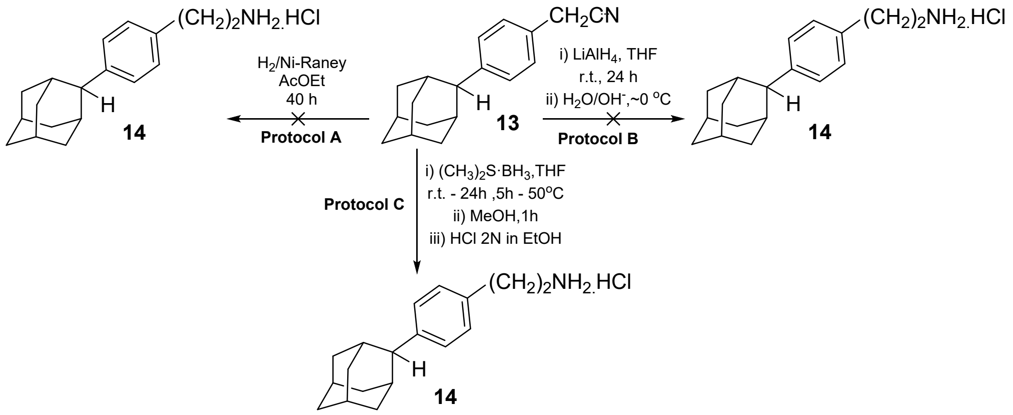

Scheme S1.Reduction protocols evaluated for the synthesis of amine **14**.

Protocol A: Raney-Nickel (0.3 ml, 50% slurry in water) was added to a solution of acetonitrile **13** (400 mg, 1.59 mmol) in ethyl acetate and the mixture was hydrogenated at room temperature and under a pressure of 55 psi for 40 h. Then the catalyst was filtered and the precipitate was washed with hot ethyl acetate. The combined filtrates were evaporated *in vacuo* but amine **14** was not isolated.

Protocol B: Phenylacetonitrile **13** (600 mg, 2.39 mmol) in anhydrous THF (10 ml) was added dropwise to a stirred suspension of LiAlH4 (1.0 g, 26 mmol) in anhydrous THF (40 ml), The reaction mixture was gently refluxed for 24 h and then was hydrolyzed at 0 ˚C by dropwise addition of ethanol, water and NaOH 10% solution. The inorganic hydroxides were removed by filtration and washed with hot THF. The combined filtrates were evaporated under reduced pressure and water was added into the residue. The resulting mixture was extracted with ethyl acetate and the combined organic layers were acidified by the dropwise addition of a HCl 10% solution, under stirring and cooling. No product was isolated.

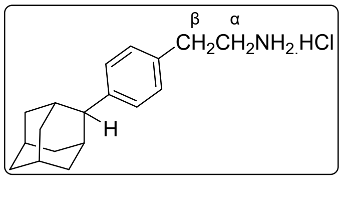

Protocol C: To a stirred solution of nitrile **13**[1] (200 mg, 0.8 mmol) in anhydrous tetrahydrofuran (15 mL), a 2 M borane dimethyl sulfide complex (BMS) in toluene (1.5 mL) was added dropwise under stirring and cooling. The solution was stirred at room temperature for 24 hours under an argon atmosphere. The next day, the reaction mixture was gently refluxed for 3 hours. The reaction mixture was stirred until it reached room temperature, methanol (~20 mL) was added, and stirring continued for another 0.5 hours. The solvents were then removed under vacuum, and 2.5 M ethanolic HCl solution was added to the residue (until pH ~1). After one day in the refrigerator, the precipitated hydrochloride salt was collected by filtration and dried

over P_2O_5. Yield: 190 mg (81%) of a white crystalline product. M.p.: 216 °C (MeOH/Et_2O) (dec); ^{1}H-NMR (400 MHz, DMSO-d_6), δ(ppm): 1.50-1.53 (d, 2H, J ≈ 13, 4,9-H_{eq}), 1.70-1.73 (~d, 5H, 4,9-H_{ax}, 5,6-H), 1.89-1.97 (m, 5H, 7,8,10-H), 2.43 (~s, 2H, 1,3-H), 2.84-2.88 (~t, 2H, β-CH_2), 2.92 (s, 1H, 2-H), 3.00-3.04 (~t, 2H, α-H), 7.20-7.22 (d, 2H, J ≈ 8.2 Hz, 3,5- H_{ar}), 7.29-7.31 (d, 2H, J ≈ 8.2 Hz, 2,6- H_{ar}), 8.03 (s, 3H, NH_2.HCl). The free base was obtained from its hydrochloride salt by addition of a saturated Na_2CO_3 solution, followed by extraction with ethyl acetate using standard procedures.

Conformational Studies

The ^{1}H-NMR and ^{13}C-NMR spectra of **6a-f** in $CDCl_3$ revealed the presence of mixtures of *E*- and *Z*-isomers due to the restricted rotation around the amide bond. Conformational studies were carried out on a series of amide derivatives to investigate their geometric isomerism and conformational behavior. The dihedral angles between consecutive bonds were evaluated, with the *E*-isomers exhibiting a dihedral angle of approximately 180° and the *Z*-isomers around 0°. The energy differences between the two conformations were significant, and the predominant isomer of each derivative was determined *via* the integration values of the characteristic proton signals in the ^{1}H-NMR spectra. In general, the *E*-isomer had higher peaks integration values, corresponding to its more stable conformation.

Indicatively, the ^{1}H-NMR data of the compound **6b** are shown in **Table S1**, along with its graphical representation of the relationship between the energy and the dihedral angle of the amide bond (**FigureS1**). From the grid scan of energy *versus* the dihedral angle of the amide group, it becomes apparent that the (*E*) conformation is more stable (dihedral angle = 180°), with a difference in energy ΔE=33 kJ/mol compared to the (*Z*) conformation (dihedral angle = 0°). Moreover, the relative integrations of the peaks of the triazole hydrogen 5-H in the NMR spectrum shows that the E/Z ratio is 4:1.

All structures were designed using the Maestro software (Schrödinger Suite 2018-2). The analysis of dihedral angles was done using the MacroModel program with OPLS3 force field and TNCG (Truncated Newton Conjugate Gradient) as the minimization method. The maximum number of iterations was set to 10,000, with a convergence threshold of 0.01. A dielectric constant of 4.81 was used to simulate $CHCl_3$ as a solvent.

Table S1. ^{1}H-NMR data of derivative **6b** in $CDCl_3$.

**Cmpd**	**Conf**	**^{1}H-NMR ($CDCl_3$) δ(ppm)**				
		**α-CH_2**	**α'-CH_2**	**β'-CH_2**	**NH**	**5-H_t**
**6b**	*E*	3.50	3.67-3.71	4.41-4.44	5.67	7.98
**6b**	*Z*	3.53	3.67-3.71	4.41-4.44	5.67	7.92

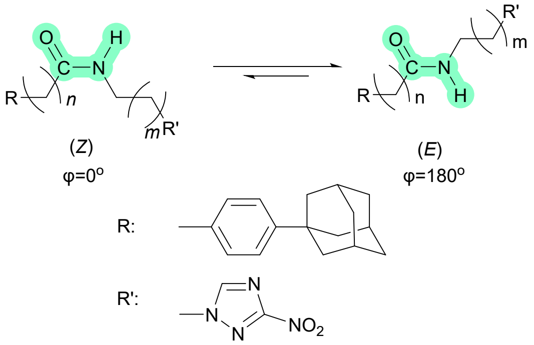

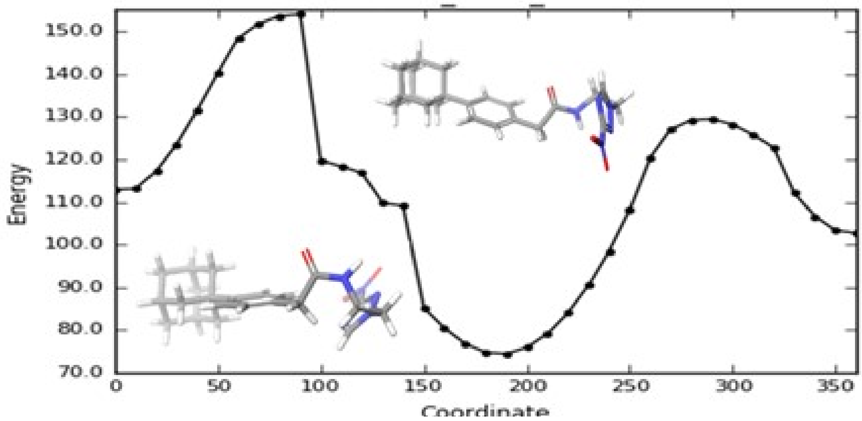

Figure S1. Graphical representation of the relationship between energy and dihedral angle of the amide bond of derivative **6b** in $CHCl_3$.

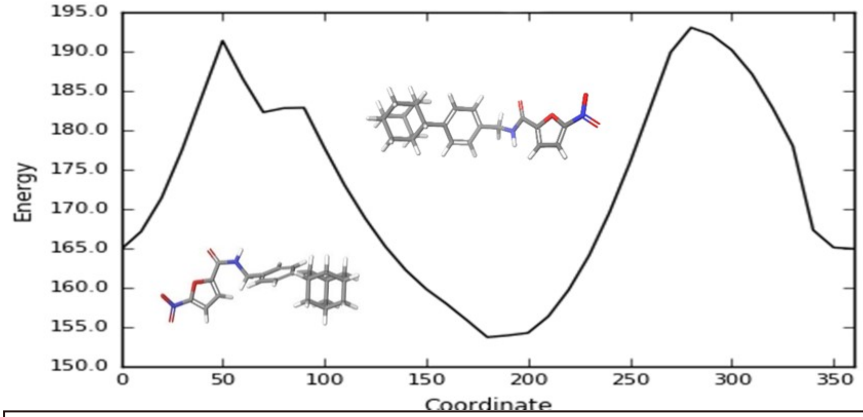

From the grid scan of energy versus the dihedral angle of the of amide group, it is clear that (*E*) conformation is more stable (the dihedral angle is 180°), with a difference in energy ΔE=11.35 kJ/mol compared to the (*Z*) conformation (the dihedral angle is 0°).

Figure S2. Graphical representation of the relationship between energy and dihedral angle of the amide bond of derivative **2b** in $CHCl_3$.

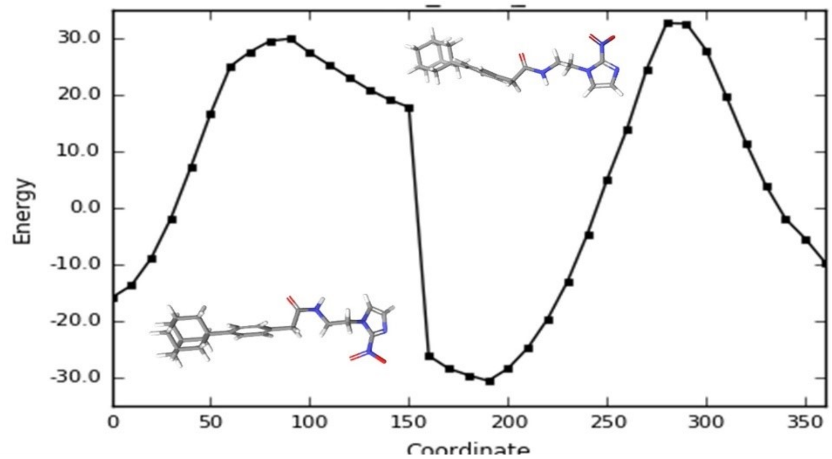

From the grid scan of energy versus the dihedral angle of theamide group, it is clear that (*E*) conformation is more stable (the dihedral angle is 180°), with a difference in energy ΔE=13 kJ/mol compared to the (*Z*) conformation (the dihedral angle is 0°)

Figure S3. Graphical representation of the relationship between energy and dihedral angle of the amide bond of derivative **5a** in $CHCl_3$.

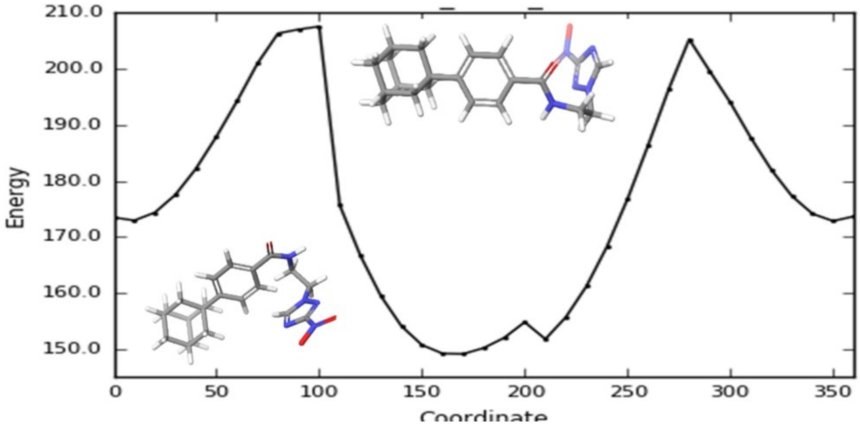

From the grid scan of energy versus the dihedral angle of the amide group, it is clear that (*E*) conformation is more stable (the dihedral angle is 180°), with a difference in energy ΔE=22 kJ/mol compared to the (*Z*) conformation (the dihedral angle is 0°)

Figure S4. Graphical representation of the relationship between energy and dihedral angle of the amide bond of derivative **6a** in $CHCl_3$.

In silico assessment of the compounds' drug-likeness

Table S2: Physicochemical properties and drug-likeness of the synthetic nitroheterocyclic aromatic adamantane amides compounds using the SwissADME platform.

Molecules	MW (g/mol)	MLogP	Rotatable Bonds	HBA	HBD	TPSA (Å²)	Water Solubility (ESOL)	Druglikeness Lipinski	Druglikeness Ghose	Druglikeness Veber	Druglikeness Egan	Druglikeness Muegge
**1a**	380.44	3.46	6	4	1	88.06	-5.66	Yes	Yes	Yes	Yes	No
**1b**	394.47	3.67	7	4	1	88.06	-5.96	Yes	Yes	Yes	Yes	No
**2a**	366.42	3.51	5	4	1	88.06	-5.50	Yes	Yes	Yes	Yes	No
**2b**	380.44	3.46	6	4	1	88.06	-5.48	Yes	Yes	Yes	Yes	No
**2c**	394.47	3.67	7	4	1	88.06	-5.78	Yes	Yes	Yes	Yes	No
**3a**	380.45	2.84	6	4	1	92.74	-4.70	Yes	Yes	Yes	Yes	Yes
**3b**	394.48	2.80	7	4	1	92.74	-4.82	Yes	Yes	Yes	Yes	Yes
**3c**	408.49	3.01	8	4	1	92.74	-5.08	Yes	Yes	Yes	Yes	Yes
**4a**	380.45	2.84	6	4	1	92.74	-4.52	Yes	Yes	Yes	Yes	Yes
**4b**	394.48	2.80	7	4	1	92.74	-4.64	Yes	Yes	Yes	Yes	Yes
**4c**	408.50	3.01	8	4	1	92.74	-4.89	Yes	Yes	Yes	Yes	Yes
**5a**	408.50	3.01	8	4	1	92.74	-4.97	Yes	Yes	Yes	Yes	Yes

**5b**	422.53	3.22	9	4	1	92.74	-5.14	Yes	Yes	Yes	Yes	Yes
**6a**	395.46	3.27	7	5	1	105.63	-4.93	Yes	Yes	Yes	Yes	Yes
**6b**	409.49	3.23	8	5	1	105.63	-4.90	Yes	Yes	Yes	Yes	Yes
**6c**	423.52	3.44	9	5	1	105.63	-5.10	Yes	Yes	Yes	Yes	Yes
**6d**	409.49	3.49	8	5	1	105.63	-4.73	Yes	Yes	Yes	Yes	Yes
**6e**	423.52	3.44	9	5	1	105.63	-4.70	Yes	Yes	Yes	Yes	Yes
**6f**	437.54	3.65	10	5	1	105.63	-5.34	Yes	Yes	Yes	Yes	No
**7a**	366.42	3.51	5	4	1	88.06	-5.69	Yes	Yes	Yes	Yes	No
**7b**	381.43	3.05	6	5	1	105.63	-4.66	Yes	Yes	Yes	Yes	Yes
**7c**	381.43	3.05	6	5	1	105.63	-4.98	Yes	Yes	Yes	Yes	Yes

In silico toxicity assessment of the compounds

Table S3: Toxicity assessment of the synthetic aromatic adamantane 5-nitrofuran-2-carboxamides **1a**,**b** and **2a–c** using the ProTox-3.0 platform*.

Prediction & Probability					
	**1a**	**1b**	**2a**	**2b**	**2c**
**Organ toxicity**					
**Hepatotoxicity**	Inactive 0.54	Inactive 0.57	Active 0.50	Inactive 0.54	Inactive 0.56
**Neurotoxicity**	Inactive 0.72	Inactive 0.70	Inactive 0.76	Inactive 0.72	Inactive 0.69
**Nephrotoxicity**	Inactive 0.57	Inactive 0.55	Inactive 0.57	Inactive 0.55	Inactive 0.54
**Respiratory toxicity**	Active 0.64	Active 0.73	Inactive 0.55	Active 0.64	Active 0.73
**Cardiotoxicity**	Inactive 0.63	Inactive 0.66	Inactive 0.64	Inactive 0.61	Inactive 0.64
**Toxicity end points**					
**Carcinogenicity**	Active 0.77	Active 0.76	Active 0.82	Active 0.80	Active 0.79
**Immunotoxicity**	Inactive 0.97	Inactive 0.89	Inactive 0.99	Inactive 0.99	Inactive 0.97
**Mutagenicity**	Active 0.84	Active 0.84	Active 0.96	Active 0.91	Active 0.92
**Cytotoxicity**	Inactive 0.67	Inactive 0.67	Inactive 0.79	Inactive 0.69	Inactive 0.68
**BBB-barrier**	Active 0.79	Active 0.77	Active 0.81	Inactive 0.79	Active 0.77
**Tox21 pathways**					
**Aryl hydrocarbon Receptor (AhR)**	Inactive 0.76	Inactive 0.77	Inactive 0.59	Inactive 0.65	Inactive 0.71
**Androgen Receptor (AR)**	Inactive 0.96	Inactive 0.96	Inactive 0.98	Inactive 0.98	Inactive 0.97
**Androgen Receptor Ligand Binding Domain (AR-LBD)**	Inactive 0.94	Inactive 0.95	Inactive 0.97	Inactive 0.95	Inactive 0.96
**Aromatase**	Inactive 0.88	Inactive 0.91	Inactive 0.92	Inactive 0.92	Inactive 0.93
**Estrogen Receptor Alpha (ER)**	Inactive 0.80	Inactive 0.80	Inactive 0.76	Inactive 0.80	Inactive 0.79
**Estrogen Receptor Ligand Binding Domain (ER-LBD)**	Inactive 0.93	Inactive 0.94	Inactive 0.91	Inactive 0.94	Inactive 0.95
**Peroxisome Proliferator Activated Receptor Gamma (PPAR-Gamma)**	Inactive 0.96	Inactive 0.95	Inactive 0.93	Inactive 0.95	Inactive 0.95
**Nuclear factor (erythroid-derived 2)-like 2/antioxidant responsive element (nrf2/ARE)**	Inactive 0.90	Inactive 0.91	Inactive 0.89	Inactive 0.89	Inactive 0.91

**Heat shock factor response element (HSE)**	Inactive 0.90	Inactive 0.91	Inactive 0.89	Inactive 0.89	Inactive 0.91
**Mitochondrial Membrane Potential (MMP)**	Active 0.57	Inactive 0.57	Active 0.60	Active 0.56	Inactive 0.57
**Phosphoprotein (Tumor Supressor) p53**	Inactive 0.81	Inactive 0.85	Inactive 0.86	Inactive 0.81	Inactive 0.86
**ATPase family AAA domain-containing protein 5 (ATAD5)**	Inactive 0.89	Inactive 0.88	Inactive 0.84	Inactive 0.87	Inactive 0.85
**Molecular Initiating Events**					
**Thyroid hormone receptor alpha (THRα)**	Inactive 0.80	Inactive 0.82	Inactive 0.84	Inactive 0.82	Inactive 0.83
**Thyroid hormone receptor beta (THRβ)**	Inactive 0.72	Inactive 0.80	Inactive 0.77	Inactive 0.69	Inactive 0.76
**Transtyretrin (TTR)**	Inactive 0.78	Inactive 0.87	Inactive 0.63	Inactive 0.75	Inactive 0.84
**Ryanodine receptor (RYR)**	Inactive 0.92	Inactive 0.88	Inactive 0.95	Inactive 0.92	Inactive 0.87
**GABA receptor (GABAR)**	Inactive 0.69	Inactive 0.64	Inactive 0.71	Inactive 0.68	Inactive 0.62
**Achetylcholinesterase (AChE)**	Inactive 0.65	Inactive 0.58	Inactive 0.78	Inactive 0.66	Inactive 0.58
**Metabolism**					
**Cytochrome CYP1A2**	Inactive 0.70	Inactive 0.77	Active 0.54	Inactive 0.61	Inactive 0.69
**Cytochrome CYP2C19**	Inactive 0.62	Inactive 0.64	Inactive 0.70	Inactive 0.66	Inactive 0.67
**Cytochrome CYP2C9**	Active 0.52	Inactive 0.52	Active 0.51	Active 0.50	Inactive 0.54
**Cytochrome CYP2D6**	Inactive 0.65	Inactive 0.61	Inactive 0.77	Inactive 0.66	Inactive 0.61
**Cytochrome CYP3A4**	Inactive 0.59	Inactive 0.62	Inactive 0.60	Inactive 0.61	Inactive 0.64
**Cytochrome CYP2E1**	Inactive 0.98	Inactive 0.99	Inactive 0.99	Inactive 0.98	Inactive 0.99

*Probability values range from 0 to 1, where 0 indicates no likelihood of occurrence and 1 indicates absolute certainty

Table S4: Toxicity assessment of the synthetic aromatic adamantane 2-nitroimidazole amides **3a-c**, **4a-c** and **5a,b** using the ProTox-3.0 platform*.

Prediction & Probability								
	**3a**	**3b**	**3c**	**4a**	**4b**	**4c**	**5a**	**5b**
**Organ toxicity**								
**Hepatotoxicity**	Inactive 0.52	Inactive 0.60	Inactive 0.65	Inactive0.52	Inactive 0.60	Inactive 0.65	Inactive 0.65	Inactive 0.66
**Neurotoxicity**	Inactive 0.50	Inactive 0.51	Inactive 0.54	Active 0.51	Inactive 0.50	Inactive 0.52	Inactive 0.54	Inactive 0.52
**Nephrotoxicity**	Inactive 0.62	Inactive 0.62	Inactive 0.61	Inactive 0.64	Inactive 0.64	Inactive 0.63	Inactive 0.61	Inactive 0.59
**Respiratory toxicity**	Active 0.72	Active 0.73	Active 0.73	Active 0.71	Active 0.72	Active 0.72	Active 0.73	Active 0.75
**Cardiotoxicity**	Inactive 0.80	Inactive 0.81	Inactive 0.82	Inactive 0.79	Inactive 0.80	Inactive 0.82	Inactive 0.82	Inactive 0.84
**Toxicity end points**								
**Carcinogenicity**	Active 0.74	Active 0.73	Active 0.72	Active 0.75	Active 0.74	Active 0.72	Active 0.72	Active 0.72
**Immunotoxicity**	Inactive 0.99	Inactive 0.94	Inactive 0.94	Inactive 0.99	Inactive 0.98	Inactive 0.98	Inactive 0.98	Inactive 0.90
**Mutagenicity**	Active 0.73	Active 0.74	Active 0.77	Active 0.84	Active 0.84	Active 0.89	Active 0.77	Active 0.76
**Cytotoxicity**	Inactive 0.61	Inactive 0.63	Inactive 0.63	Inactive 0.60	Inactive 0.63	Inactive 0.63	Inactive 0.63	Inactive 0.63
**BBB-barrier**	Active 0.84	Active 0.83	Active 0.84	Active 0.85	Active 0.83	Active 0.84	Active 0.84	Active 0.83
**Tox21 pathways**								
**Aryl hydrocarbon Receptor (AhR)**	Inactive 0.83	Inactive 0.86	Inactive 0.86	Inactive 0.80	Inactive 0.87	Inactive 0.85	Inactive 0.86	Inactive 0.86
**Androgen Receptor (AR)**	Inactive 0.95	Inactive 0.95	Inactive 0.95	Inactive 0.94	Inactive 0.95	Inactive 0.96	Inactive 0.95	Inactive 0.94
**Androgen Receptor Ligand Binding Domain (AR-LBD)**	Inactive 0.95	Inactive 0.95	Inactive 0.96	Inactive 0.95	Inactive 0.95	Inactive 0.95	Inactive 0.96	Inactive 0.96
**Aromatase**	Inactive 0.91	Inactive 0.92	Inactive 0.93	Inactive 0.94	Inactive 0.94	Inactive 0.95	Inactive 0.93	Inactive 0.93
**Estrogen Receptor Alpha (ER)**	Inactive 0.88	Inactive 0.86	Inactive 0.84	Inactive 0.90	Inactive 0.88	Inactive 0.86	Inactive 0.84	Inactive 0.84
**Estrogen Receptor Ligand Binding Domain (ER-LBD)**	Inactive 0.95	Inactive 0.95	Inactive 0.95	Inactive 0.96	Inactive 0.97	Inactive 0.97	Inactive 0.95	Inactive 0.94
**Peroxisome Proliferator Activated Receptor Gamma (PPAR-Gamma)**	Inactive 0.92	Inactive 0.95	Inactive 0.94	Inactive 0.92	Inactive 0.95	Inactive 0.94	Inactive 0.94	Inactive 0.94
**Nuclear factor (erythroid-derived 2)-like 2/antioxidant responsive element (nrf2/ARE)**	Inactive 0.91	Inactive 0.93	Inactive 0.93	Inactive 0.91	Inactive 0.93	Inactive 0.93	Inactive 0.93	Inactive 0.95
**Heat shock factor response element (HSE)**	Inactive 0.91	Inactive 0.93	Inactive 0.93	Inactive 0.91	Inactive 0.93	Inactive 0.94	Inactive 0.93	Inactive 0.95

**Mitochondrial Membrane Potential (MMP)**	Inactive 0.61	Inactive 0.69	Inactive 0.67	Inactive 0.61	Inactive 0.70	Inactive 0.66	Inactive 0.67	Inactive 0.65
**Phosphoprotein (Tumor Supressor) p53**	Inactive 0.86	Inactive 0.87	Inactive 0.89	Inactive 0.86	Inactive 0.88	Inactive 0.90	Inactive 0.89	Inactive 0.88
**ATPase family AAA domain-containing protein 5 (ATAD5)**	Inactive 0.90	Inactive 0.90	Inactive 0.89	Inactive 0.86	Inactive 0.87	Inactive 0.86	Inactive 0.89	Inactive 0.89
**Molecular Initiating Events**								
**Thyroid hormone receptor alpha (THRα)**	Inactive 0.89	Inactive 0.89	Inactive 0.90	Inactive 0.90	Inactive 0.91	Inactive 0.92	Inactive 0.90	Inactive 0.92
**Thyroid hormone receptor beta (THRβ)**	Inactive 0.72	Inactive 0.72	Inactive 0.72	Inactive 0.64	Inactive 0.63	Inactive 0.61	Inactive 0.72	Inactive 0.79
**Transtyretrin (TTR)**	Inactive 0.90	Inactive 0.93	Inactive 0.95	Inactive 0.88	Inactive 0.92	Inactive 0.94	Inactive 0.95	Inactive 0.95
**Ryanodine receptor (RYR)**	Inactive 0.94	Inactive 0.92	Inactive 0.89	Inactive 0.93	Inactive 0.91	Inactive 0.88	Inactive 0.89	Inactive 0.89
**GABA receptor (GABAR)**	Inactive 0.60	Inactive 0.58	Inactive 0.55	Inactive 0.59	Inactive 0.56	Inactive 0.53	Inactive 0.55	Inactive 0.53
**Achetylcholinesterase (AChE)**	Inactive 0.79	Inactive 0.73	Inactive 0.65	Inactive 0.81	Inactive 0.74	Inactive 0.68	Inactive 0.65	Inactive 0.65
**Metabolism**								
**Cytochrome CYP1A2**	Inactive 0.75	Inactive 0.82	Inactive 0.84	Inactive 0.65	Inactive 0.71	Inactive 0.71	Inactive 0.84	Inactive 0.87
**Cytochrome CYP2C19**	Inactive 0.64	Inactive 0.68	Inactive 0.69	Inactive 0.69	Inactive 0.70	Inactive 0.70	Inactive 0.69	Inactive 0.75
**Cytochrome CYP2C9**	Inactive 0.51	Inactive 0.51	Inactive 0.51	Inactive 0.53	Inactive 0.53	Inactive 0.51	Inactive 0.51	Inactive 0.54
**Cytochrome CYP2D6**	Inactive 0.65	Inactive 0.60	Inactive 0.59	Inactive 0.65	Inactive 0.59	Inactive 0.56	Inactive 0.59	Inactive 0.60
**Cytochrome CYP3A4**	Inactive 0.66	Inactive 0.64	Inactive 0.65	Inactive 0.66	Inactive 0.65	Inactive 0.66	Inactive 0.65	Inactive 0.66
**Cytochrome CYP2E1**	Inactive 0.99	Inactive 0.99	Inactive 0.99	Inactive 0.99	Inactive 0.99	Inactive 0.99	Inactive 0.99	Inactive 0.99

*Probability values range from 0 to 1, where 0 indicates no likelihood of occurrence and 1 indicates absolute certainty

Table S5: Toxicity assessment of the synthetic aromatic adamantane 3-nitrotriazole amides **6a-f** using the ProTox-3.0 platform*.

**Prediction & Probability**						
	**6a**	**6b**	**6c**	**6d**	**6e**	**6f**
**Organ toxicity**						
**Hepatotoxicity**	Inactive 0.58	Inactive 0.59	Inactive 0.58	Inactive 0.58	Inactive 0.58	Inactive 0.58
**Neurotoxicity**	Inactive 0.53	Inactive 0.54	Inactive 0.53	Inactive 0.52	Inactive 0.53	Inactive 0.53
**Nephrotoxicity**	Inactive 0.53	Inactive 0.54	Inactive 0.53	Inactive 0.51	Inactive 0.53	Inactive 0.53
**Respiratory toxicity**	Active 0.68	Active 0.72	Active 0.75	Active 0.72	Active 0.75	Active 0.75
**Cardiotoxicity**	Inactive 0.84	Inactive 0.84	Inactive 0.82	Inactive 0.83	Inactive 0.82	Inactive 0.82
**Toxicity end points**						
**Carcinogenicity**	Active 0.79	Active 0.79	Active 0.79	Active 0.79	Active 0.79	Active 0.79
**Immunotoxicity**	Inactive 0.90	Inactive 0.98	Inactive 0.91	Inactive 0.58	Inactive 0.91	Inactive 0.62
**Mutagenicity**	Active 0.79	Active 0.78	Active 0.78	Active 0.79	Active 0.78	Active 0.78
**Cytotoxicity**	Inactive 0.68	Inactive 0.69	Inactive 0.68	Inactive 0.67	Inactive 0.68	Inactive 0.68
**BBB-barrier**	Active 0.83	Active 0.84	Active 0.83	Active 0.83	Active 0.83	Active 0.83
**Tox21 pathways**						
**Aryl hydrocarbon Receptor (AhR)**	Inactive 0.78	Inactive 0.81	Inactive 0.82	Inactive 0.78	Inactive 0.82	Inactive 0.82
**Androgen Receptor (AR)**	Inactive 0.96	Inactive 0.96	Inactive 0.96	Inactive 0.97	Inactive 0.96	Inactive 0.96
**Androgen Receptor Ligand Binding Domain (AR-LBD)**	Inactive 0.97	Inactive 0.97	Inactive 0.97	Inactive 0.97	Inactive 0.97	Inactive 0.97
**Aromatase**	Inactive 0.91	Inactive 0.93	Inactive 0.93	Inactive 0.91	Inactive 0.93	Inactive 0.93
**Estrogen Receptor Alpha (ER)**	Inactive 0.80	Inactive 0.80	Inactive 0.81	Inactive 0.81	Inactive 0.81	Inactive 0.81
**Estrogen Receptor Ligand Binding Domain (ER-LBD)**	Inactive 0.95	Inactive 0.95	Inactive 0.95	Inactive 0.95	Inactive 0.95	Inactive 0.95
**Peroxisome Proliferator Activated Receptor Gamma (PPAR-Gamma)**	Inactive 0.94	Inactive 0.95	Inactive 0.95	Inactive 0.94	Inactive 0.95	Inactive 0.95
**Nuclear factor (erythroid-derived 2)-like 2/antioxidant responsive element (nrf2/ARE)**	Inactive 0.92	Inactive 0.93	Inactive 0.94	Inactive 0.93	Inactive 0.94	Inactive 0.94
**Heat shock factor response element**	Inactive 0.92	Inactive 0.93	Inactive 0.94	Inactive 0.93	Inactive 0.94	Inactive 0.94

**(HSE)**						
**Mitochondrial Membrane Potential (MMP)**	Inactive 0.65	Inactive 0.67	Inactive 0.66	Inactive 0.63	Inactive 0.66	Inactive 0.66
**Phosphoprotein (Tumor Supressor) p53**	Inactive 0.87	Inactive 0.88	Inactive 0.87	Inactive 0.87	Inactive 0.87	Inactive 0.87
**ATPase family AAA domain-containing protein 5 (ATAD5)**	Inactive 0.89	Inactive 0.87	Inactive 0.88	Inactive 0.89	Inactive 0.88	Inactive 0.88
**Molecular Initiating Events**						
**Thyroid hormone receptor alpha (THRα)**	Inactive 0.89	Inactive 0.90	Inactive 0.91	Inactive 0.90	Inactive 0.91	Inactive 0.91
**Thyroid hormone receptor beta (THRβ)**	Inactive 0.81	Inactive 0.82	Inactive 0.84	Inactive 0.82	Inactive 0.84	Inactive 0.84
**Transtyretrin (TTR)**	Inactive 0.92	Inactive 0.94	Inactive 0.93	Inactive 0.92	Inactive 0.93	Inactive 0.93
**Ryanodine receptor (RYR)**	Inactive 0.88	Inactive 0.88	Inactive 0.88	Inactive 0.88	Inactive 0.88	Inactive 0.88
**GABA receptor (GABAR)**	Inactive 0.53	Inactive 0.50	Active 0.50	Inactive 0.52	Active 0.50	Active 0.50
**Achetylcholinesterase (AChE)**	Inactive 0.67	Inactive 0.65	Inactive 0.64	Inactive 0.66	Inactive 0.64	Inactive 0.64
**Metabolism**						
**Cytochrome CYP1A2**	Inactive 0.85	Inactive 0.88	Inactive 0.88	Inactive 0.85	Inactive 0.88	Inactive 0.88
**Cytochrome CYP2C19**	Inactive 0.71	Inactive 0.72	Inactive 0.73	Inactive 0.71	Inactive 0.73	Inactive 0.73
**Cytochrome CYP2C9**	Inactive 0.55	Inactive 0.53	Inactive 0.56	Inactive 0.58	Inactive 0.56	Inactive 0.56
**Cytochrome CYP2D6**	Inactive 0.62	Inactive 0.61	Inactive 0.60	Inactive 0.61	Inactive 0.60	Inactive 0.60
**Cytochrome CYP3A4**	Inactive 0.73	Inactive 0.74	Inactive 0.74	Inactive 0.73	Inactive 0.74	Inactive 0.74
**Cytochrome CYP2E1**	Inactive 0.99	Inactive 0.99	Inactive 0.99	Inactive0.99	Inactive 0.99	Inactive 0.99

*Probability values range from 0 to 1, where 0 indicates no likelihood of occurrence and 1 indicates absolute certainty.

Table S6: Toxicity assessment of the synthetic (1-adamantyl)anilinamides **7a-c** using the ProTox-3.0 platform*.

**Prediction & Probability**			
	**7a**	**7b**	**7c**
**Organ toxicity**			
**Hepatotoxicity**	Active 0.51	Active 0.52	Inactive 0.51
**Neurotoxicity**	Inactive 0.76	Inactive 0.50	Inactive 0.50
**Nephrotoxicity**	Inactive 0.60	Inactive 0.56	Inactive 0.55
**Respiratory toxicity**	Inactive 0.55	Active 0.64	Active 0.67
**Cardiotoxicity**	Inactive 0.66	Inactive 0.83	Inactive 0.82
**Toxicity end points**			
**Carcinogenicity**	Active 0.79	Active 0.80	Active 0.71
**Immunotoxicity**	Inactive 0.97	Inactive 0.98	Inactive 0.95
**Mutagenicity**	Active 0.85	Active 0.73	Active 0.71
**Cytotoxicity**	Inactive 0.75	Inactive 0.71	Inactive 0.69
**BBB-barrier**	Active 0.80	Active 0.85	Active 0.81
**Tox21 pathways**			
**Aryl hydrocarbon Receptor (AhR)**	Inactive 0.71	Inactive 0.78	Inactive 0.81
**Androgen Receptor (AR)**	Inactive 0.96	Inactive 0.97	Inactive 0.95
**Androgen Receptor Ligand Binding Domain (AR-LBD)**	Inactive 0.95	Inactive 0.97	Inactive 0.97
**Aromatase**	Inactive 0.88	Inactive 0.90	Inactive 0.90
**Estrogen Receptor Alpha (ER)**	Inactive 0.78	Inactive 0.86	Inactive 0.87
**Estrogen Receptor Ligand Binding Domain (ER-LBD)**	Inactive 0.89	Inactive 0.96	Inactive 0.96
**Peroxisome Proliferator Activated Receptor Gamma (PPAR-Gamma)**	Inactive 0.94	Inactive 0.94	Inactive 0.90
**Nuclear factor (erythroid-derived 2)-like 2/antioxidant responsive element (nrf2/ARE)**	Inactive 0.89	Inactive 0.92	Inactive 0.93
**Heat shock factor response element (HSE)**	Inactive 0.89	Inactive 0.92	Inactive 0.93
**Mitochondrial Membrane Potential (MMP)**	Active 0.62	Inactive 0.62	Inactive 0.59

**Phosphoprotein (Tumor Supressor) p53**	Inactive 0.84	Inactive 0.86	Inactive 0.88
**ATPase family AAA domain-containing protein 5 (ATAD5)**	Inactive 0.88	Inactive 0.91	Inactive 0.92
**Molecular Initiating Events**			
**Thyroid hormone receptor alpha (THRα)**	Inactive 0.82	Inactive 0.89	Inactive 0.89
**Thyroid hormone receptor beta (THRβ)**	Inactive 0.73	Inactive 0.81	Inactive 0.76
**Transtyretrin (TTR)**	Inactive 0.69	Inactive 0.90	Inactive 0.89
**Ryanodine receptor (RYR)**	Inactive 0.95	Inactive 0.93	Inactive 0.93
**GABA receptor (GABAR)**	Inactive 0.72	Inactive 0.57	Inactive 0.59
**Achetylcholinesterase (AChE)**	Inactive 0.77	Inactive 0.77	Inactive 0.76
**Metabolism**			
**Cytochrome CYP1A2**	Inactive 0.60	Inactive 0.78	Inactive 0.79
**Cytochrome CYP2C19**	Inactive 0.66	Inactive 0.68	Inactive 0.68
**Cytochrome CYP2C9**	Active 0.54	Inactive 0.52	Inactive 0.51
**Cytochrome CYP2D6**	Inactive 0.74	Inactive 0.65	Inactive 0.66
**Cytochrome CYP3A4**	Inactive 0.59	Inactive 0.75	Inactive 0.69
**Cytochrome CYP2E1**	Inactive 0.99	Inactive 0.99	Inactive 0.99

*Probability values range from 0 to 1, where 0 indicates no likelihood of occurrence and 1 indicates absolute certainty.

Heat map of antiparasitic activity

To provide a comparative visualization of compound activities across parasite cell lines, we generated a heat map of pIC_{50} values.

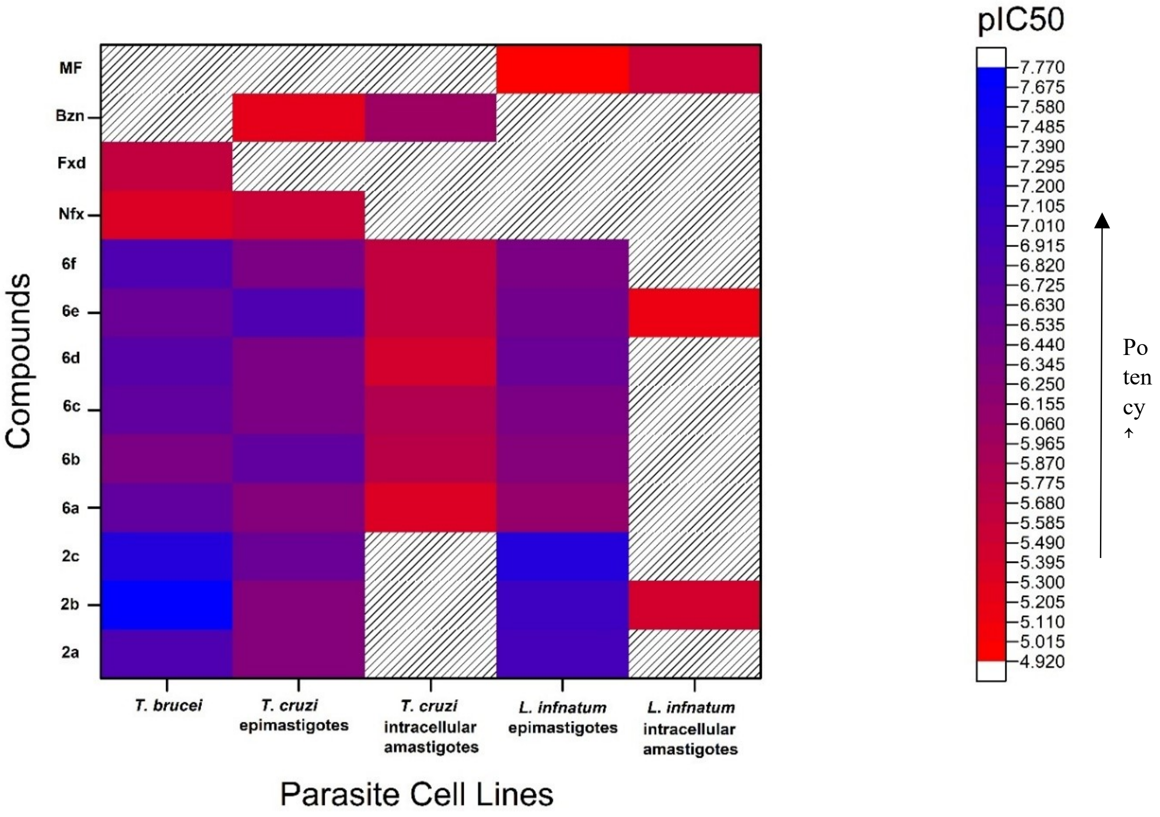

Figure S5. Heat map of pIC_{50} values (–$\log_{10}$(IC_{50} in µM)) for the tested compounds against the indicated parasite cell lines. Higher pIC_{50} values correspond to lower IC_{50} values (greater potency). Only compounds with sufficient data across multiple cell lines are included. IC_{50} values in µM were converted to pIC_{50} using the formula $pIC_{50} = 6 - \log_{10}(IC_{50}\ [\mu M])$.

^{1}H and ^{13}C NMR spectra

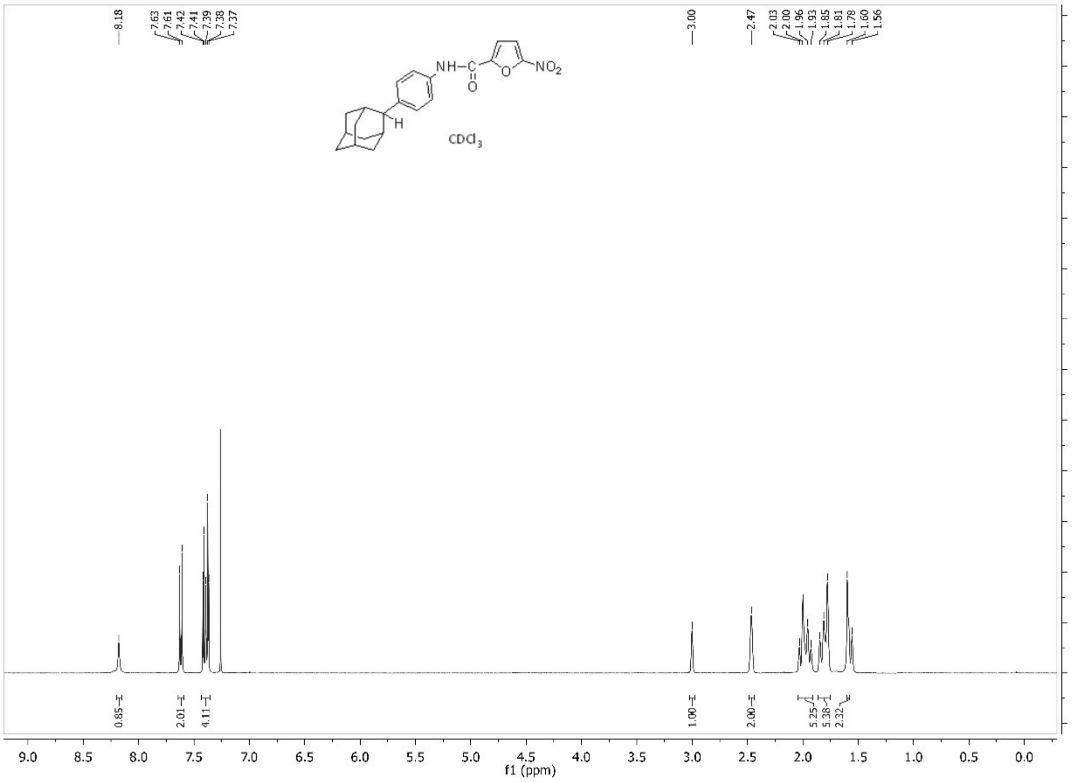

Figure S6. ^1H spectrum of **2a** in $CDCl_3$

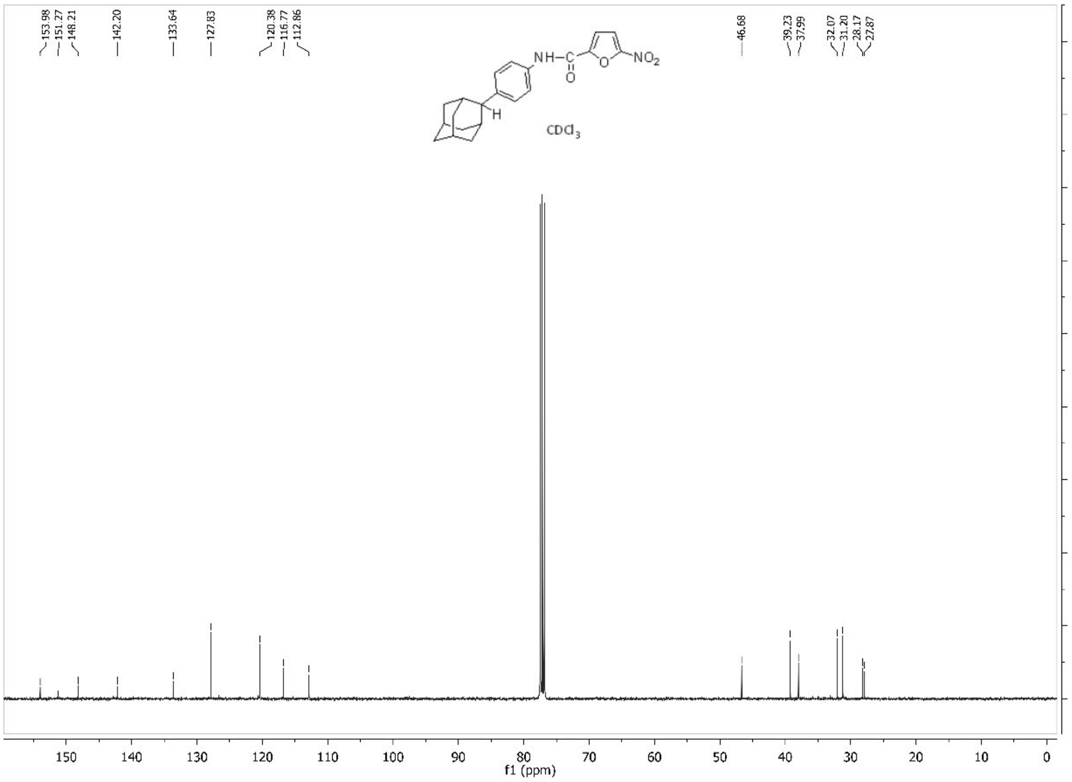

Figure S7. ^{13}C spectrum of **2a** in $CDCl_3$

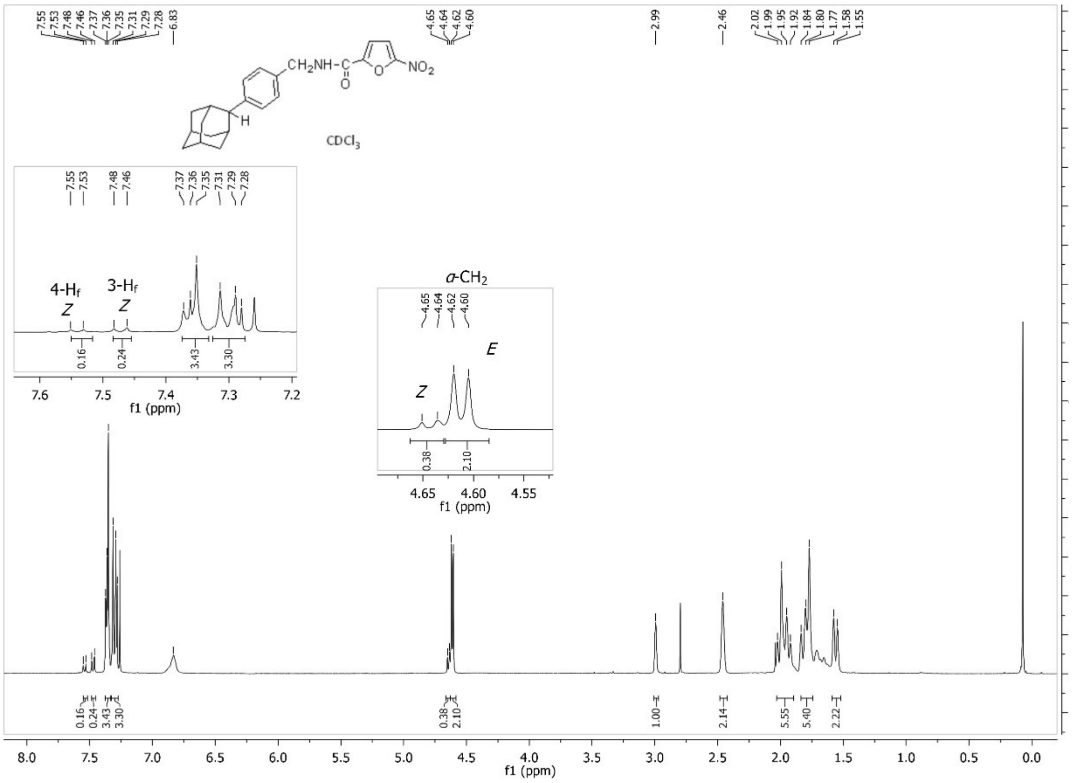

Figure S8. ^{1}H spectrum of **2b** in $CDCl_3$

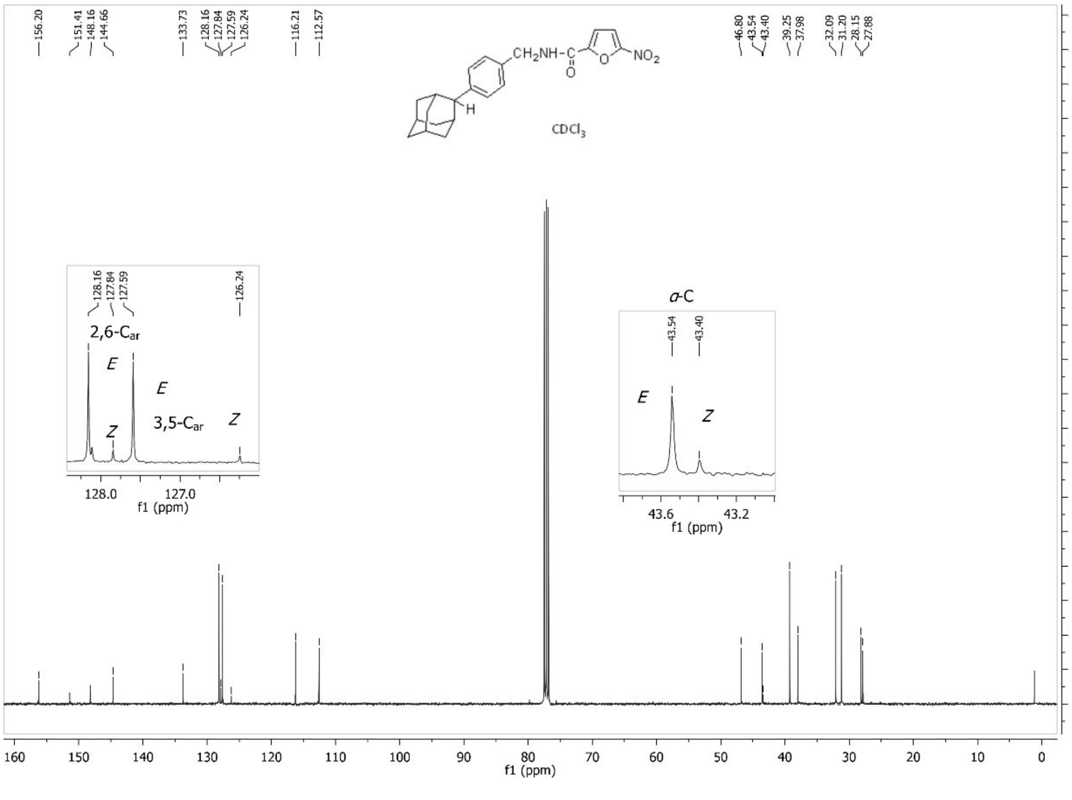

Figure S9. ^{13}C spectrum of **2b** in $CDCl_3$

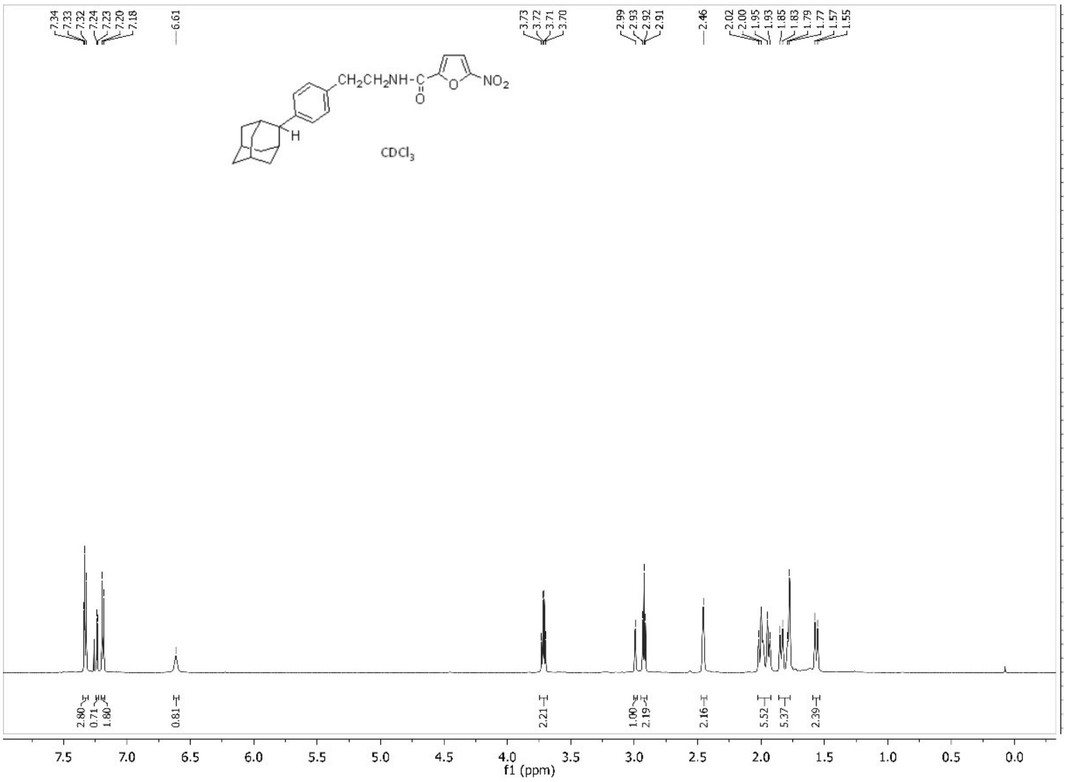

Figure S10. ^{1}H spectrum of **2c** in $CDCl_3$

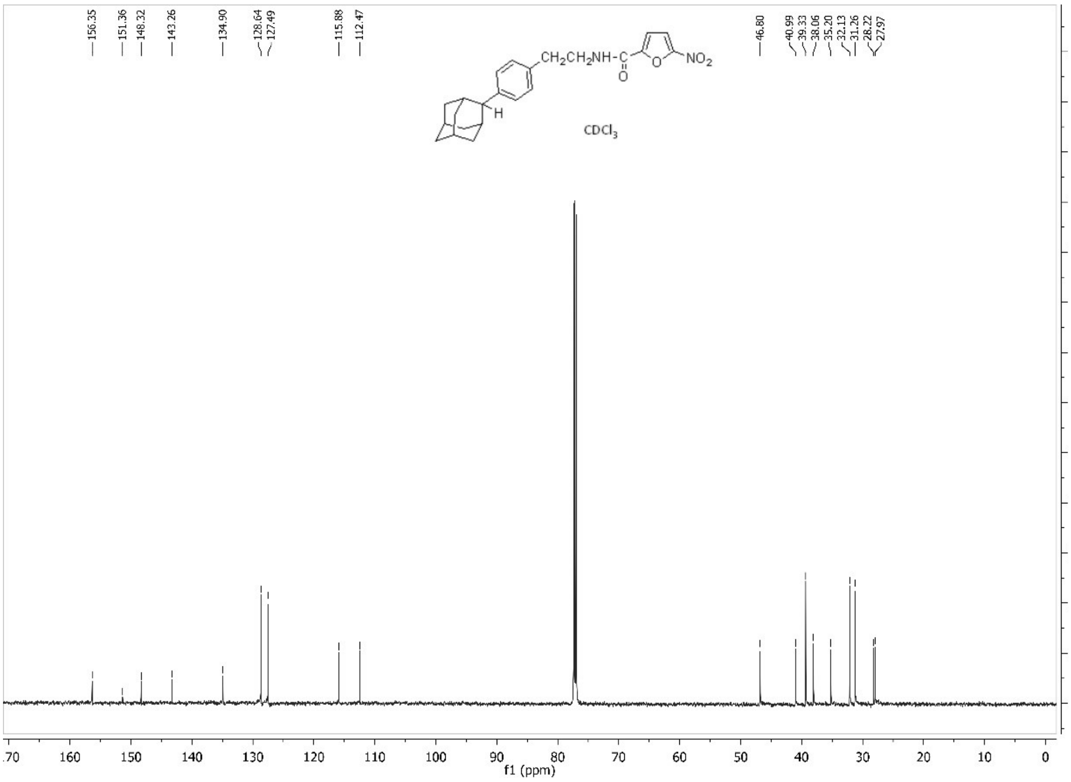

Figure S11. ^{13}C spectrum of **2a** in $CDCl_3$

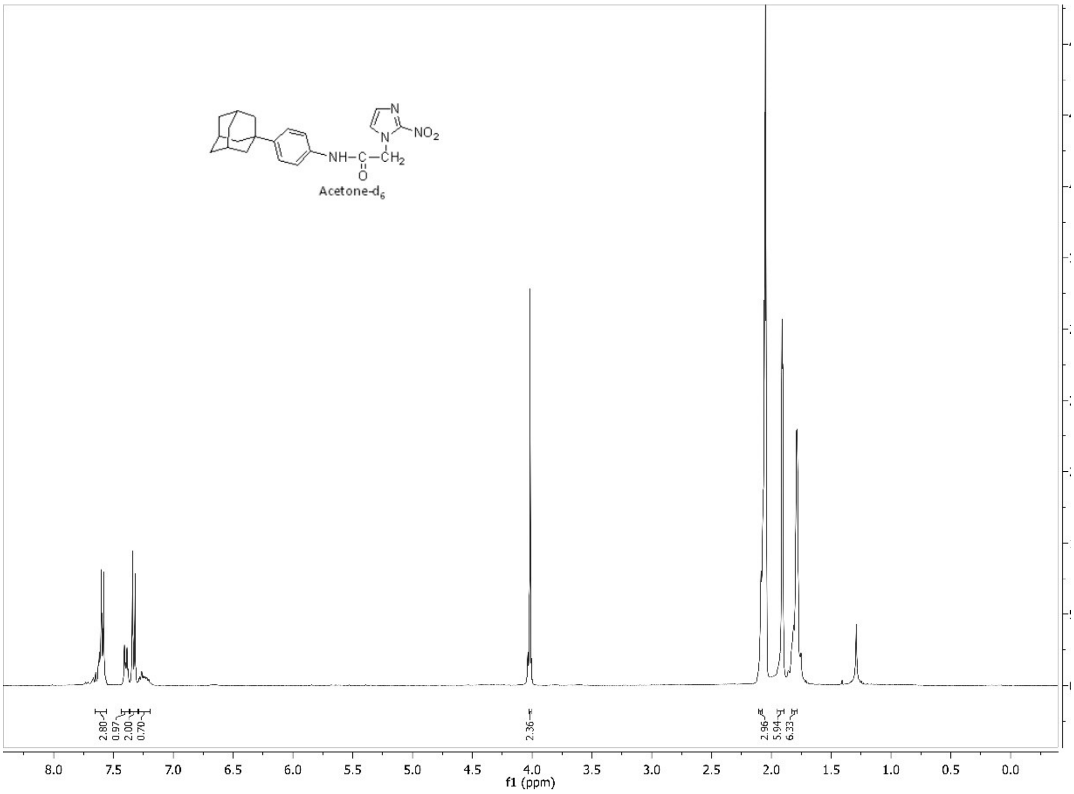

Figure S12. ^{1}H spectrum of **3a** in acetone-d$_6$

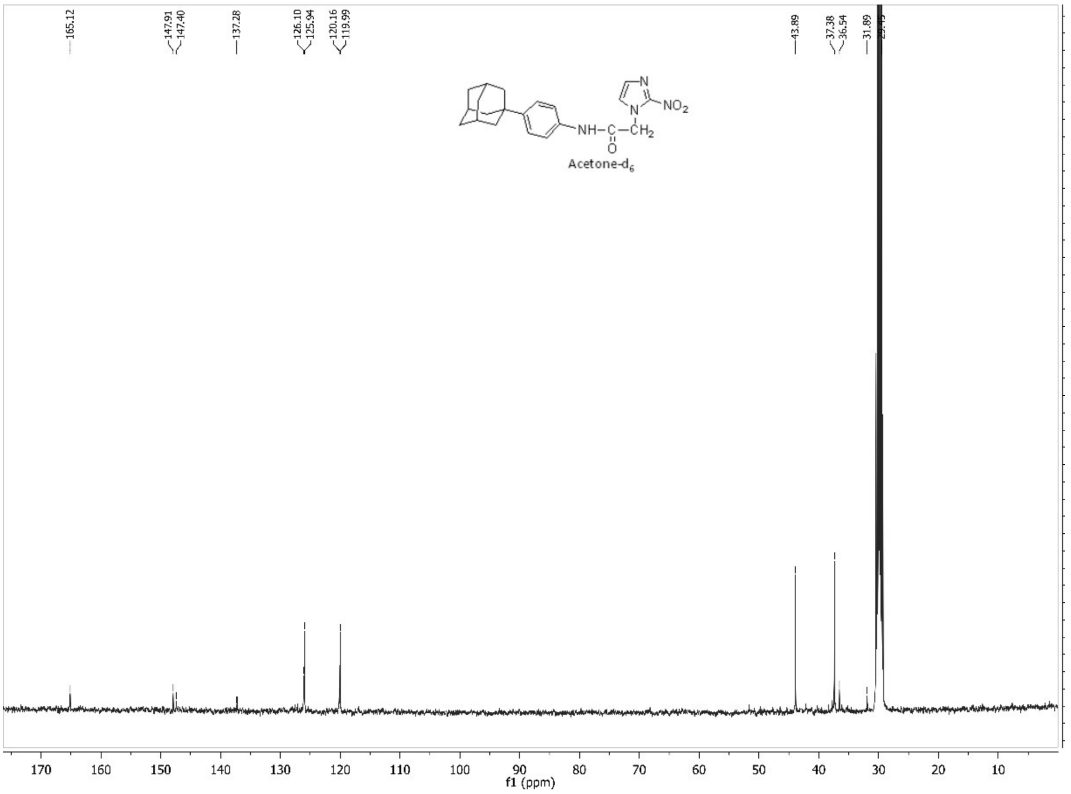

Figure S13. ^{13}C spectrum of **3a** in acetone-d$_6$

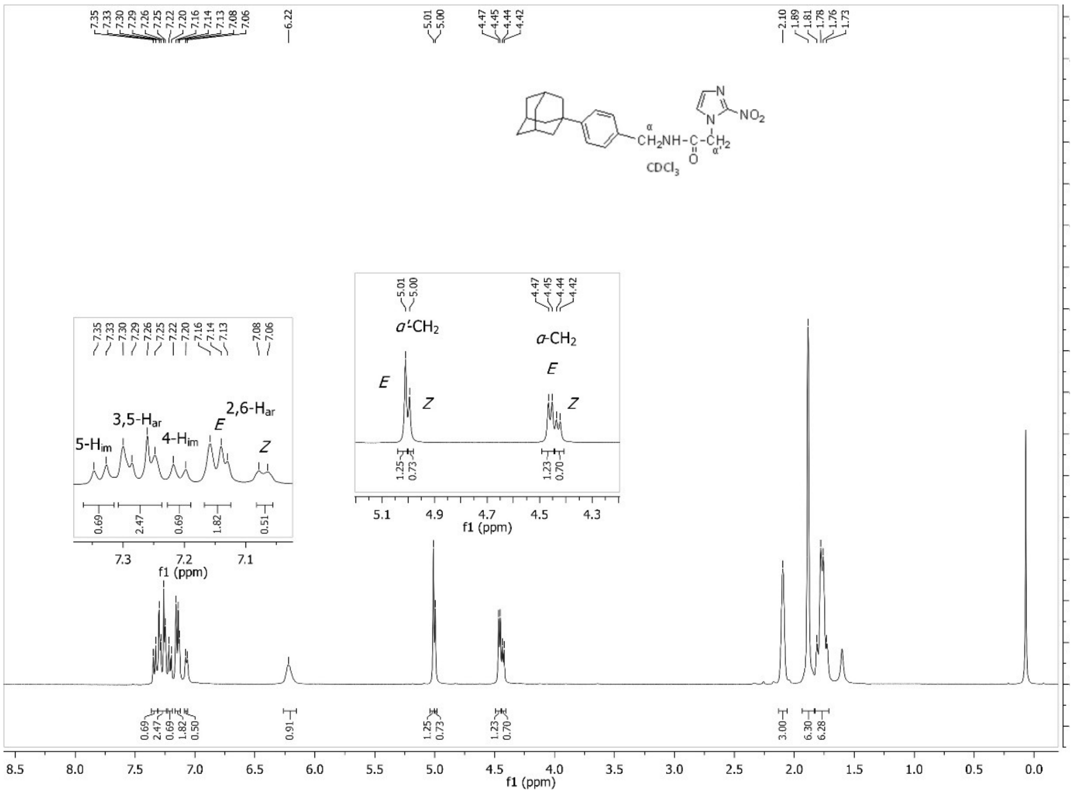

Figure S14. ^{1}H spectrum of **3b** in $CDCl_3$

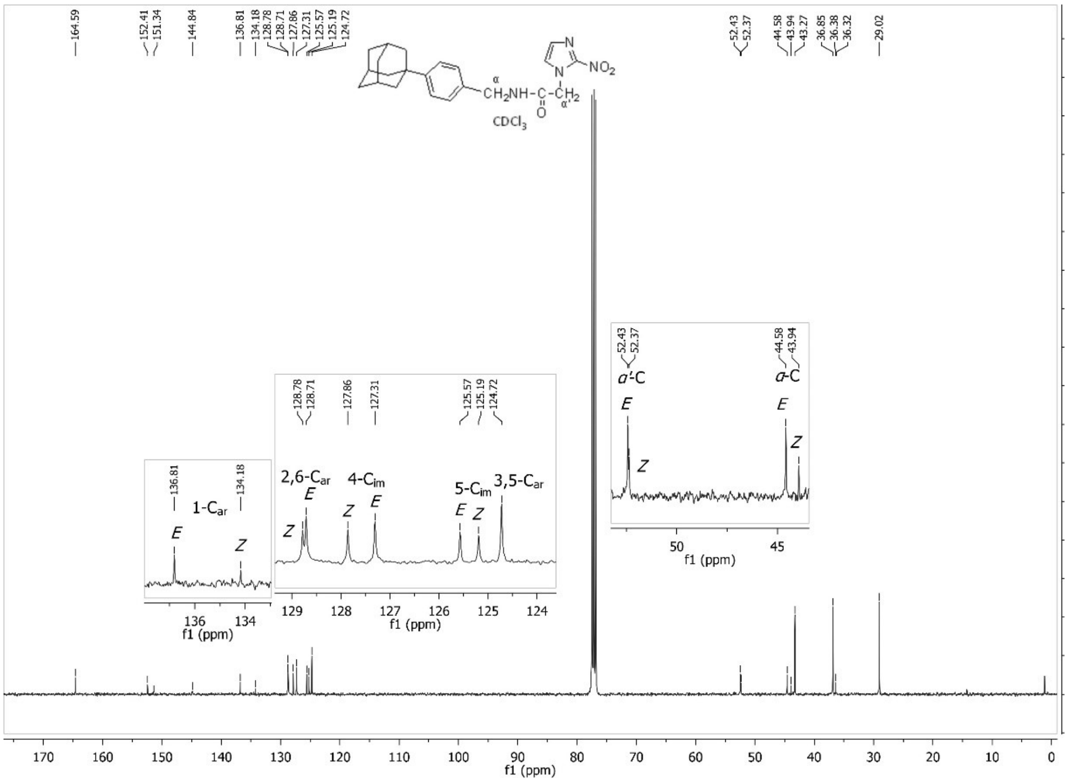

Figure S15. ^{13}C spectrum of **3b** in $CDCl_3$

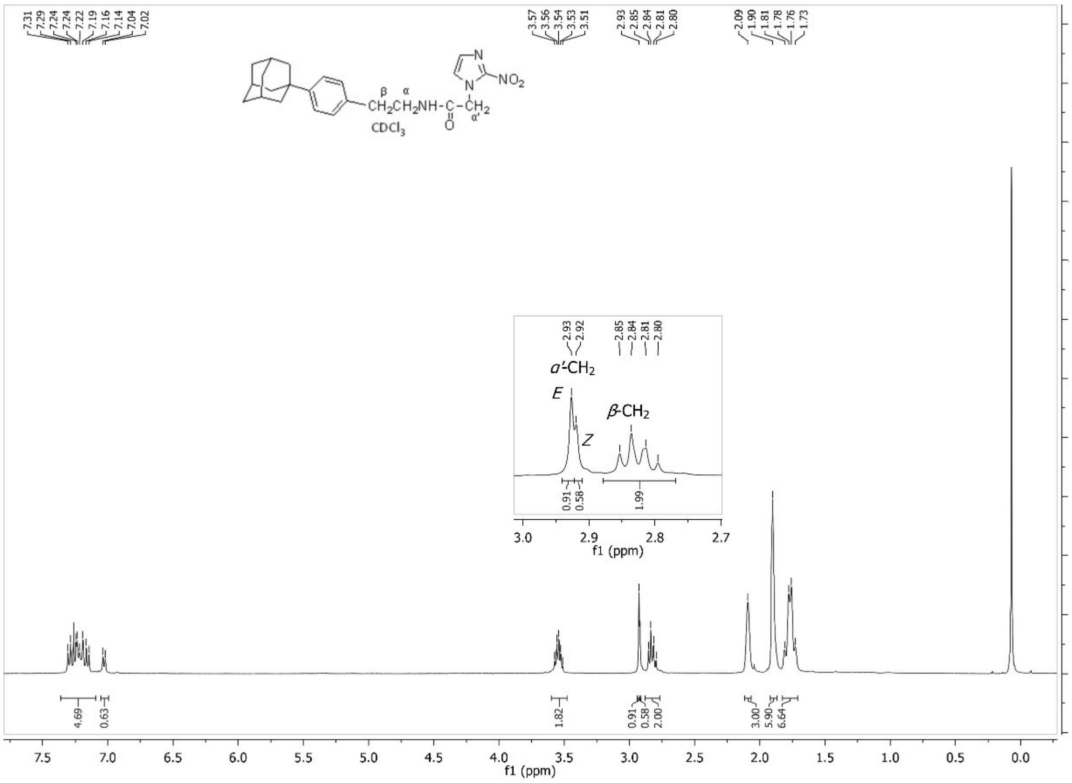

Figure S16. ^{1}H spectrum of **3c** in $CDCl_3$

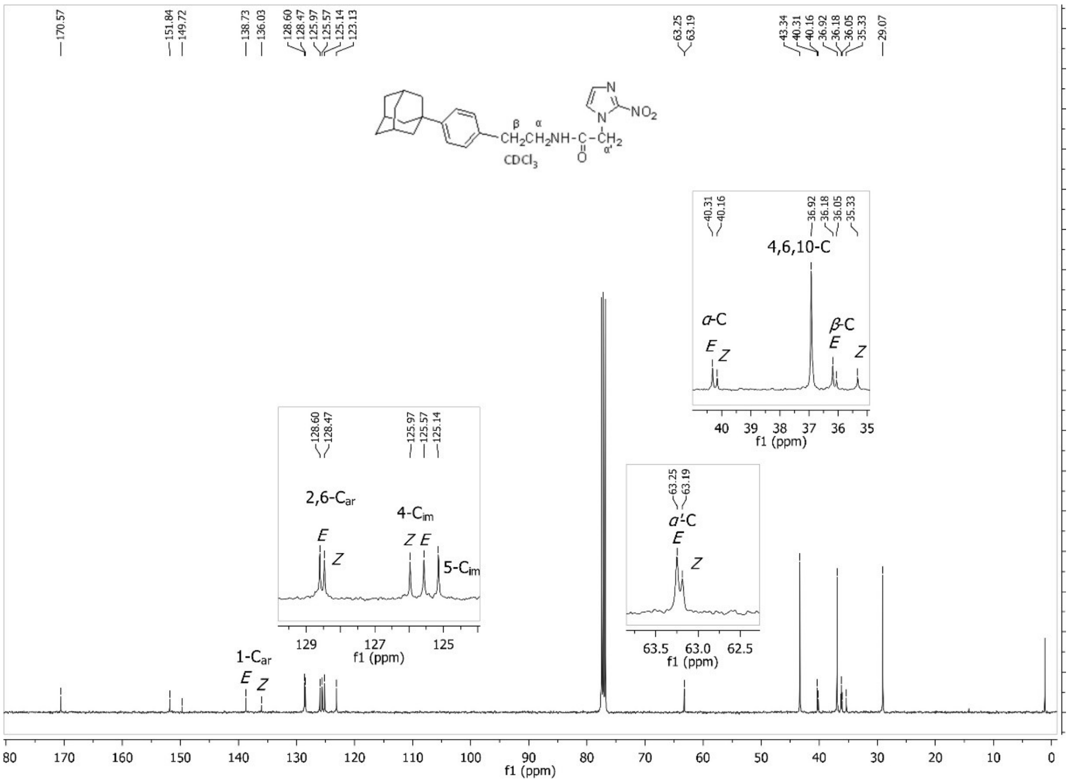

Figure S17. ^{13}C spectrum of **3c** in $CDCl_3$

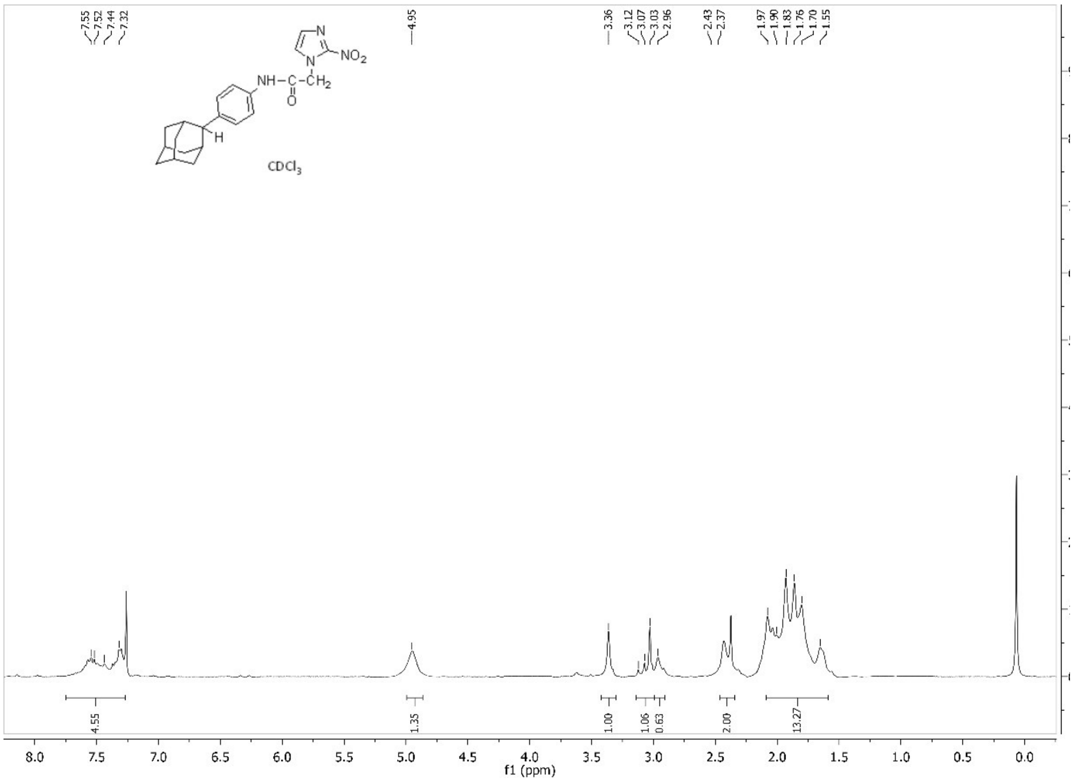

Figure S18. ^{1}H spectrum of **4a** in $CDCl_3$

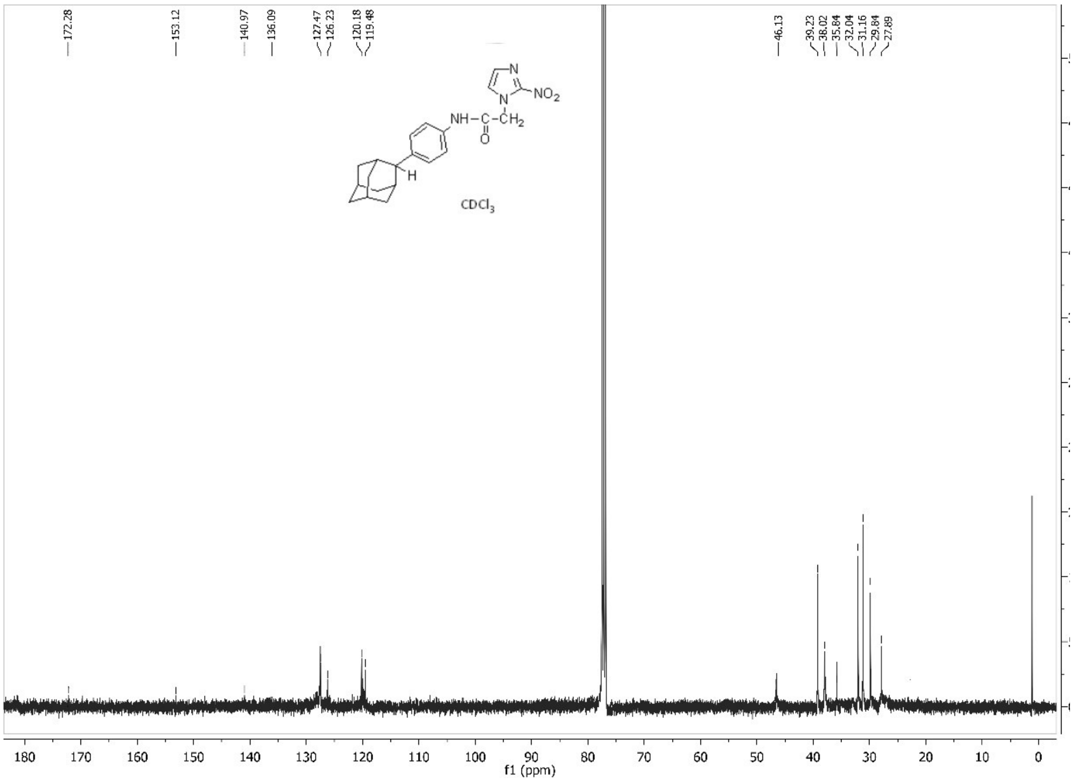

Figure S19. ^{13}C spectrum of **4a** in $CDCl_3$

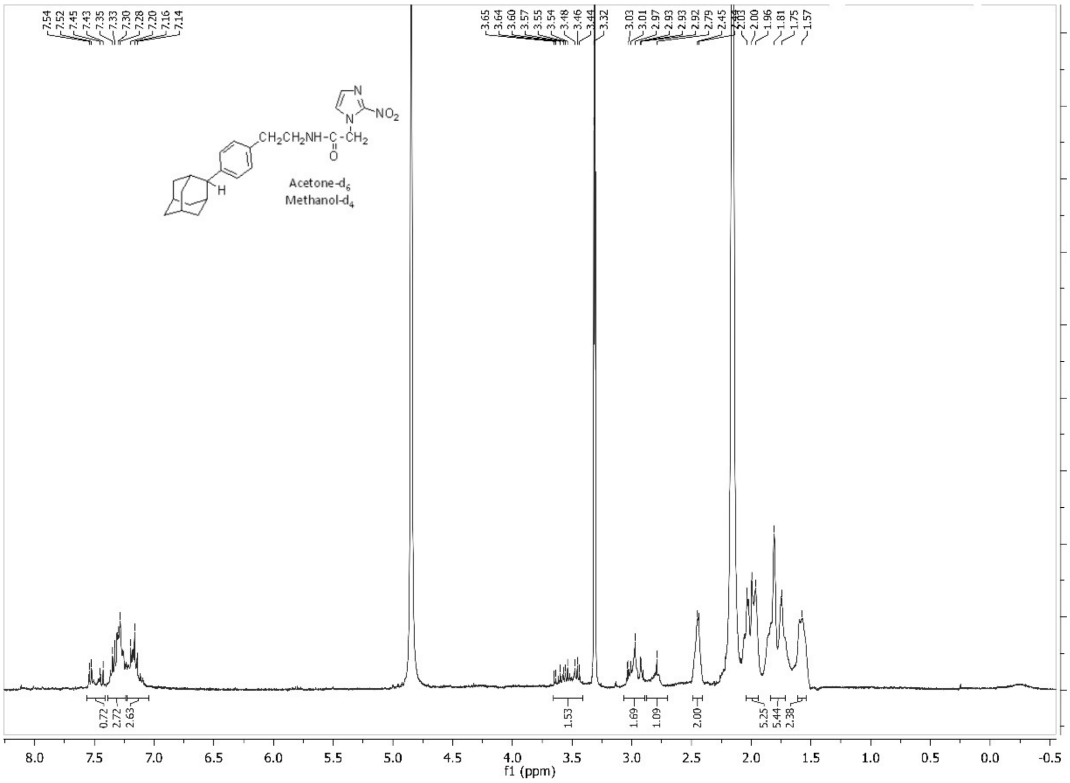

Figure S20. ^{1}H spectrum of **4c** in acetone-d_6/methanol-d_4

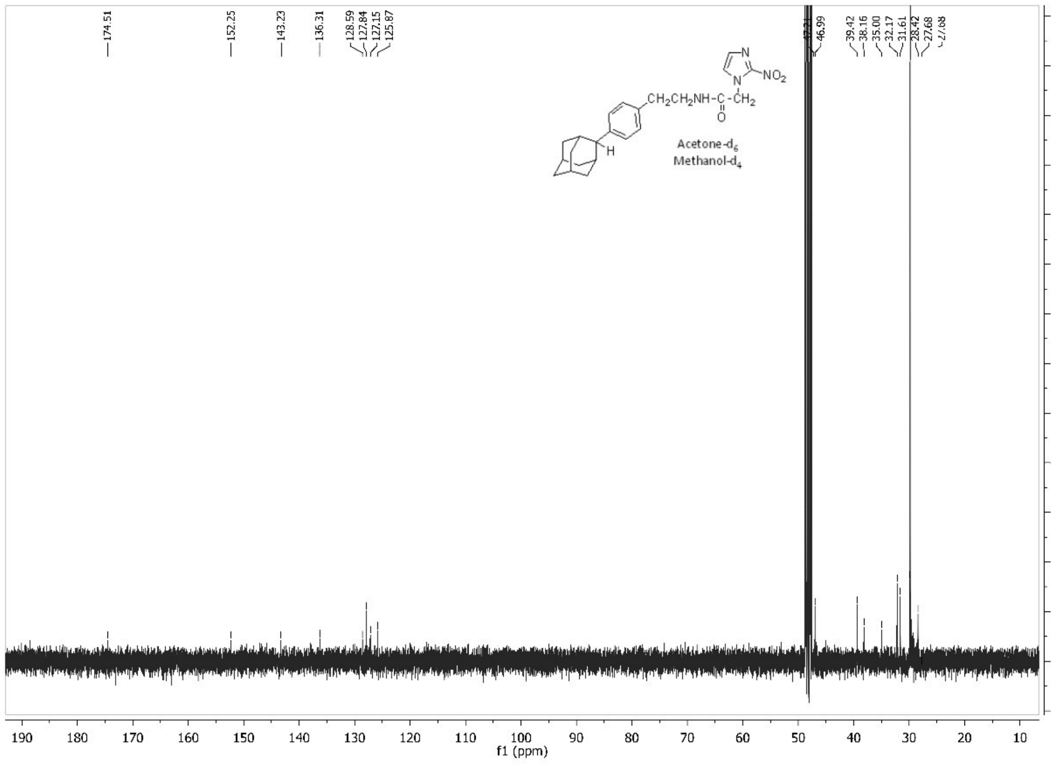

Figure S21. ^{13}C spectrum of **4c** in acetone-d_6/methanol-d_4

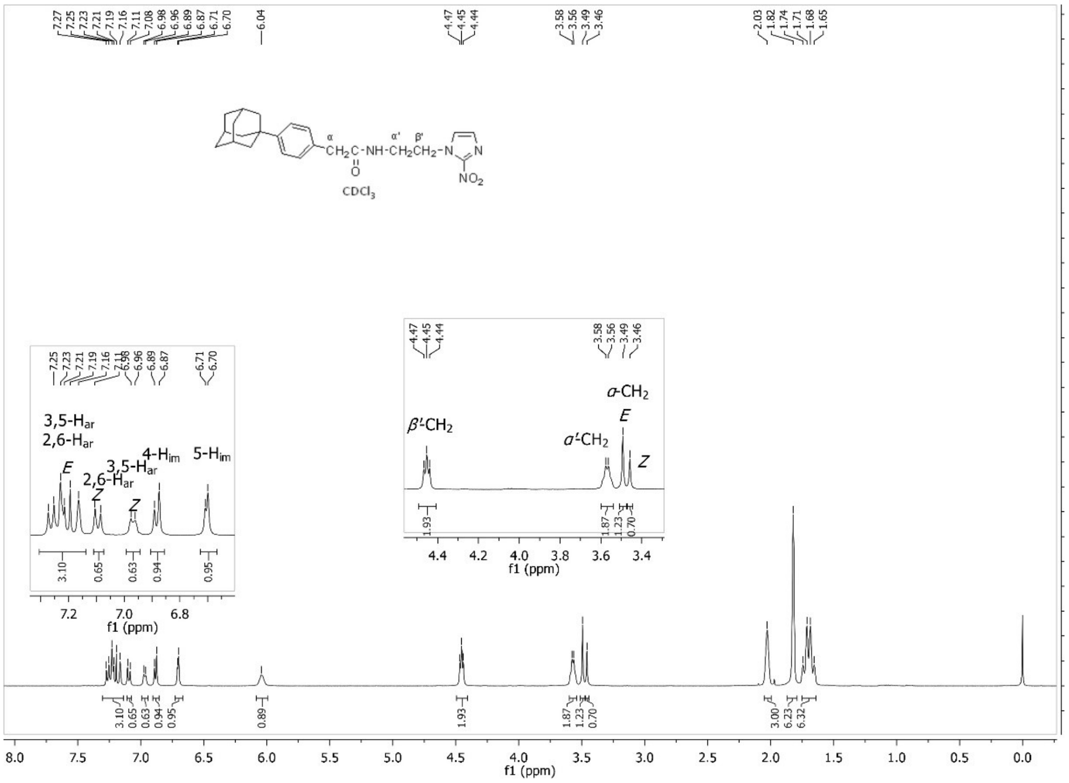

Figure S22. ^{1}H spectrum of **5a** in $CDCl_3$

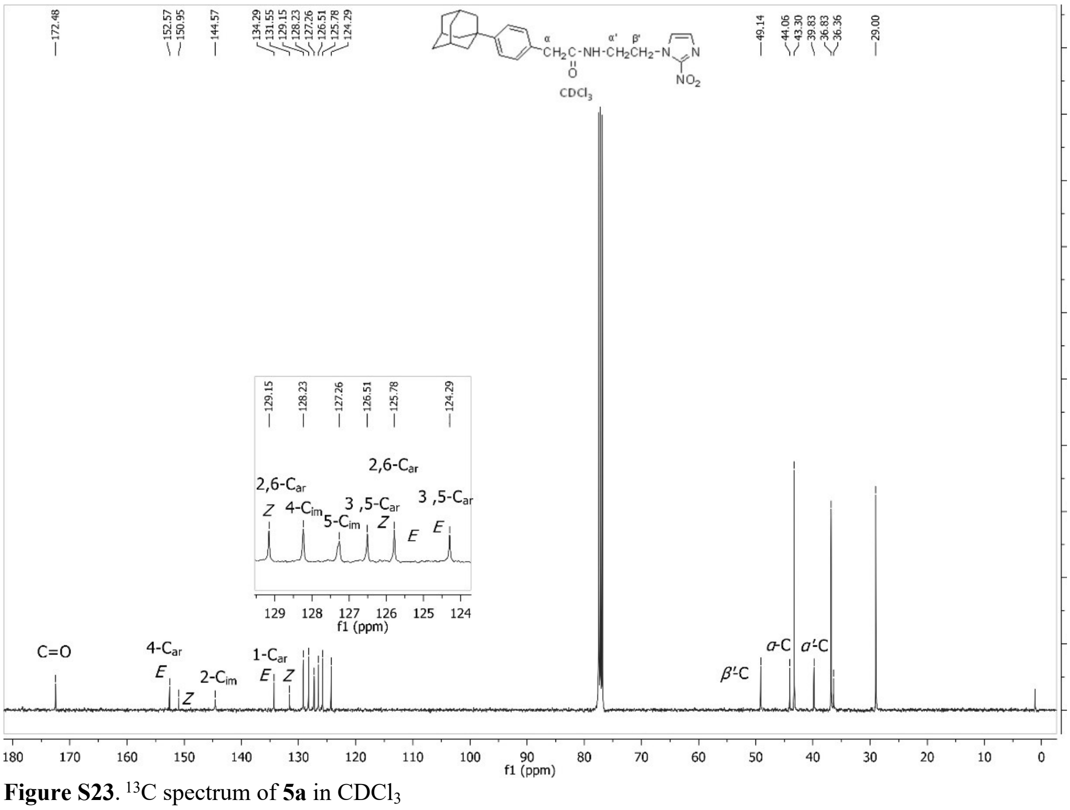

Figure S23. ^{13}C spectrum of **5a** in $CDCl_3$

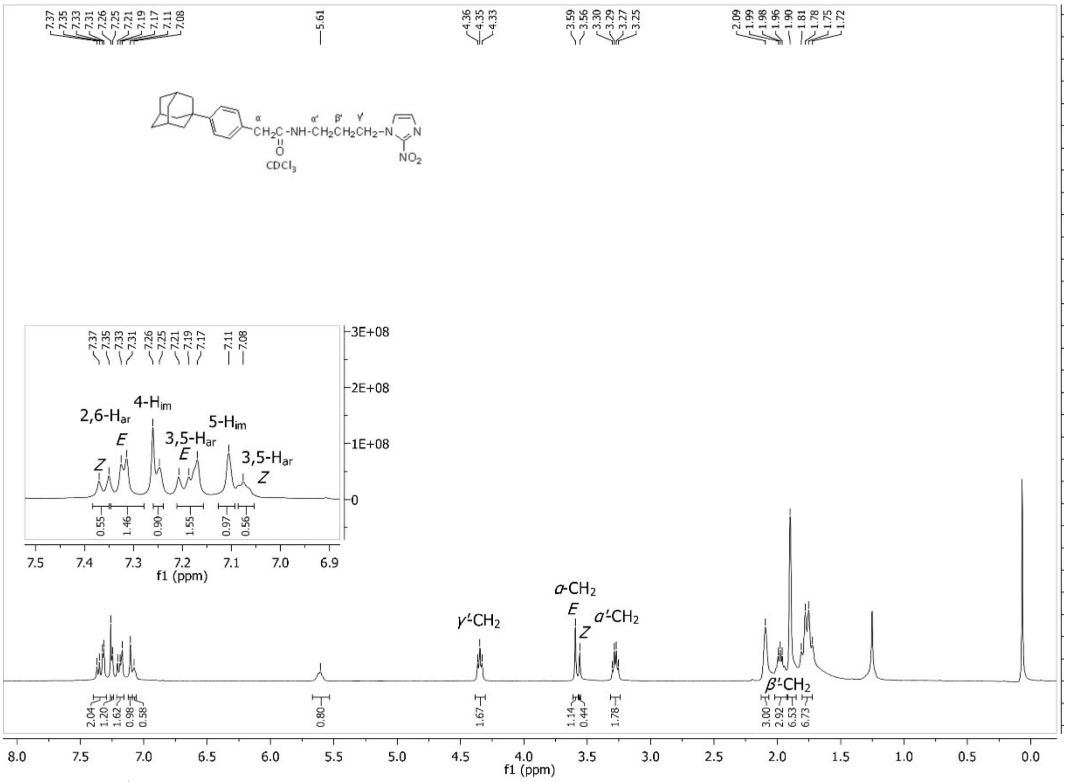

Figure S24. ^{1}H spectrum of **5b** in $CDCl_3$

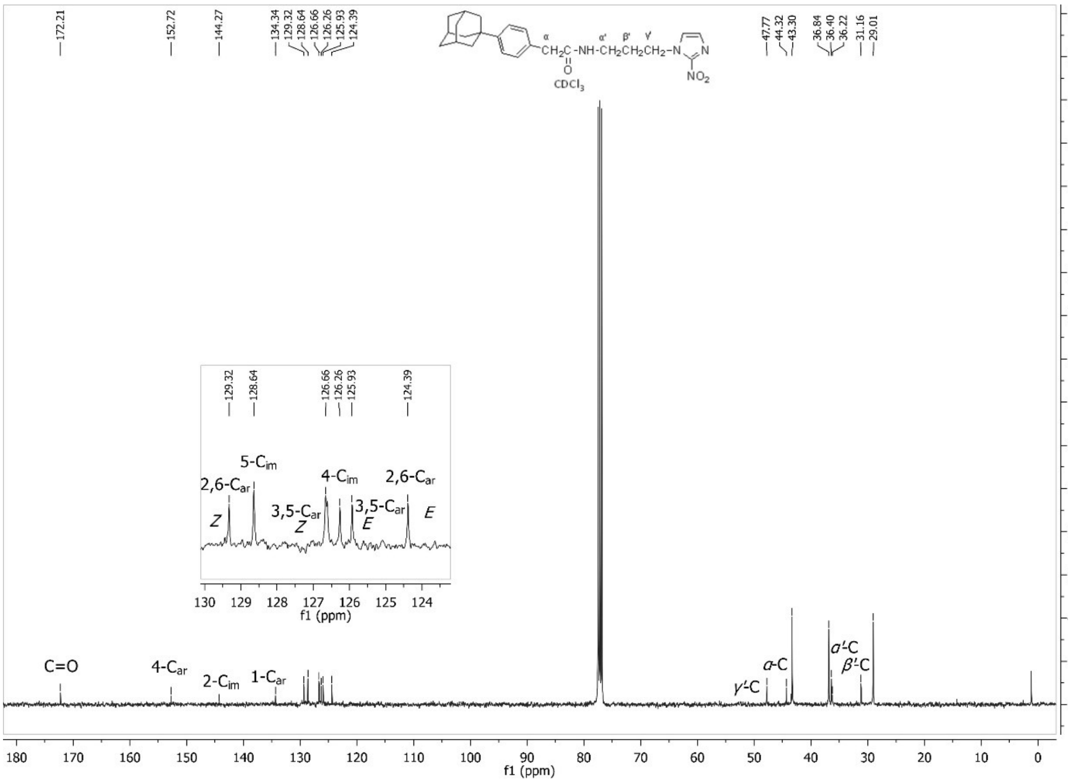

Figure S25. ^{13}C spectrum of **5b** in $CDCl_3$

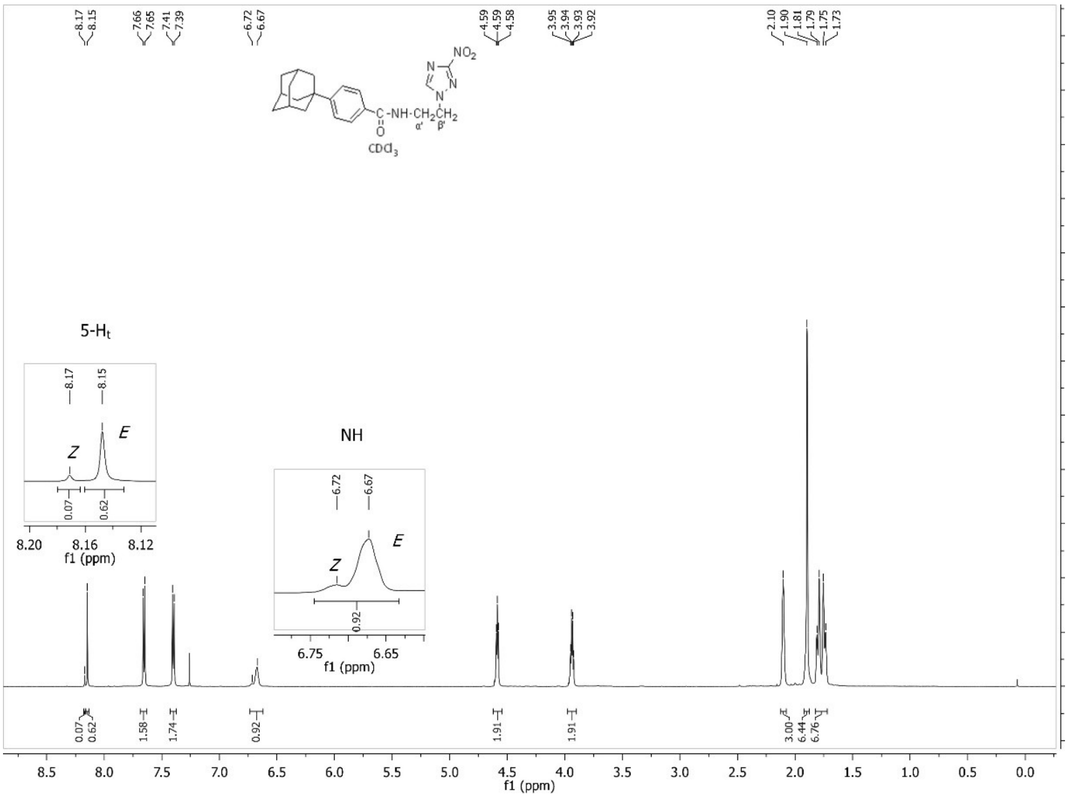

Figure S26. ^{1}H spectrum of **6a** in $CDCl_3$

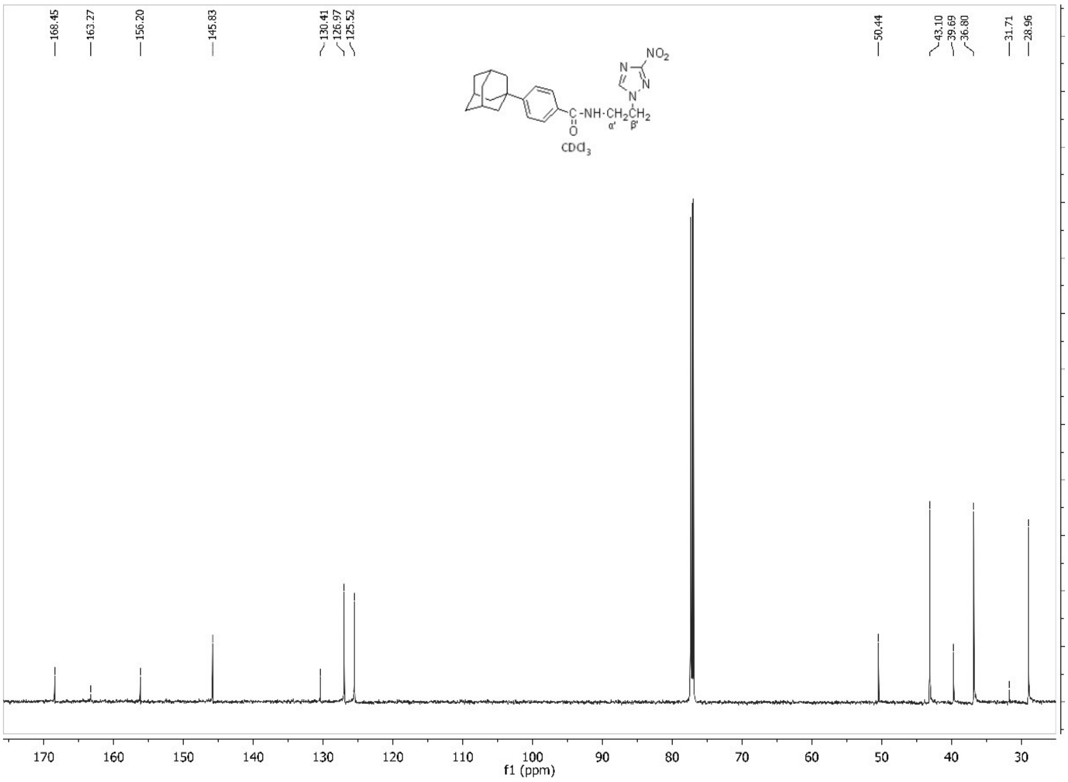

Figure S27. ^{13}C spectrum of **6a** in $CDCl_3$

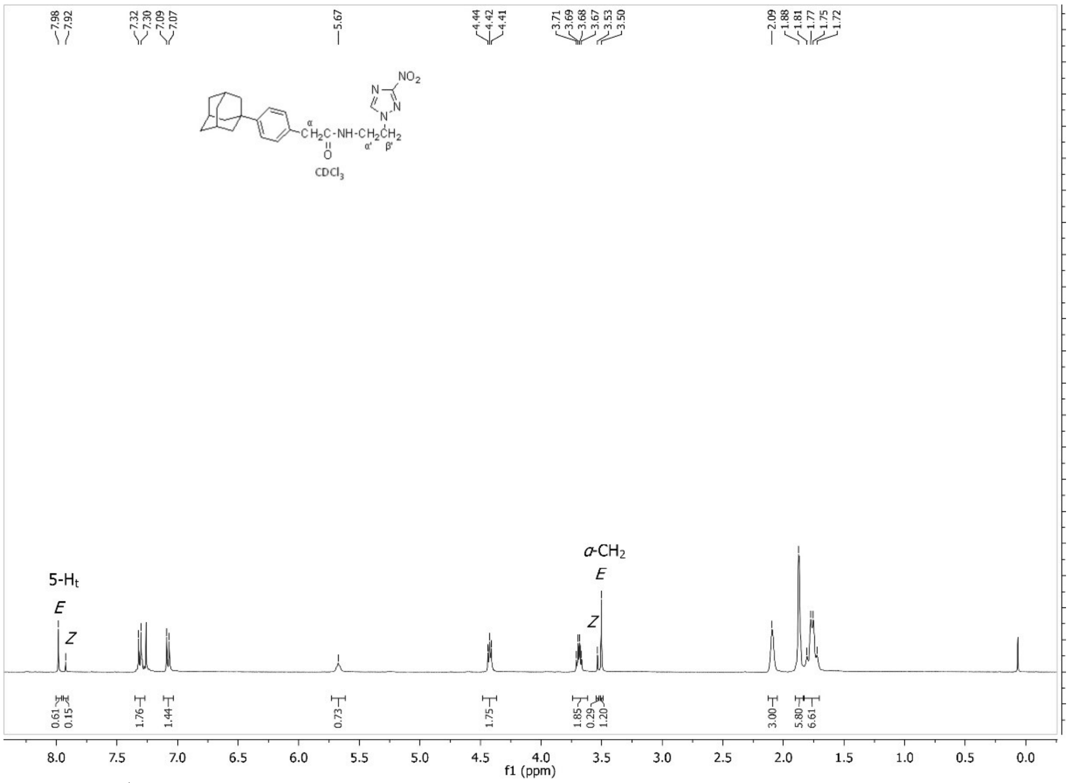

Figure S28. ^{1}H spectrum of **6b** in $CDCl_3$

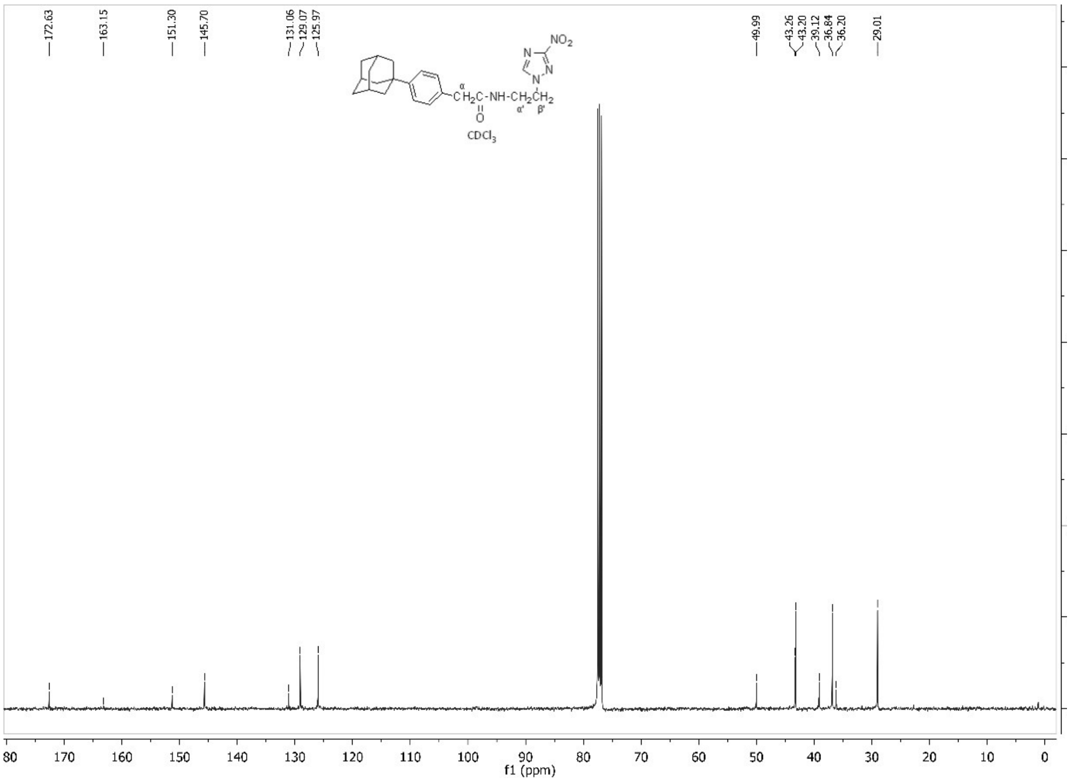

Figure S29. ^{13}C spectrum of **6b** in $CDCl_3$

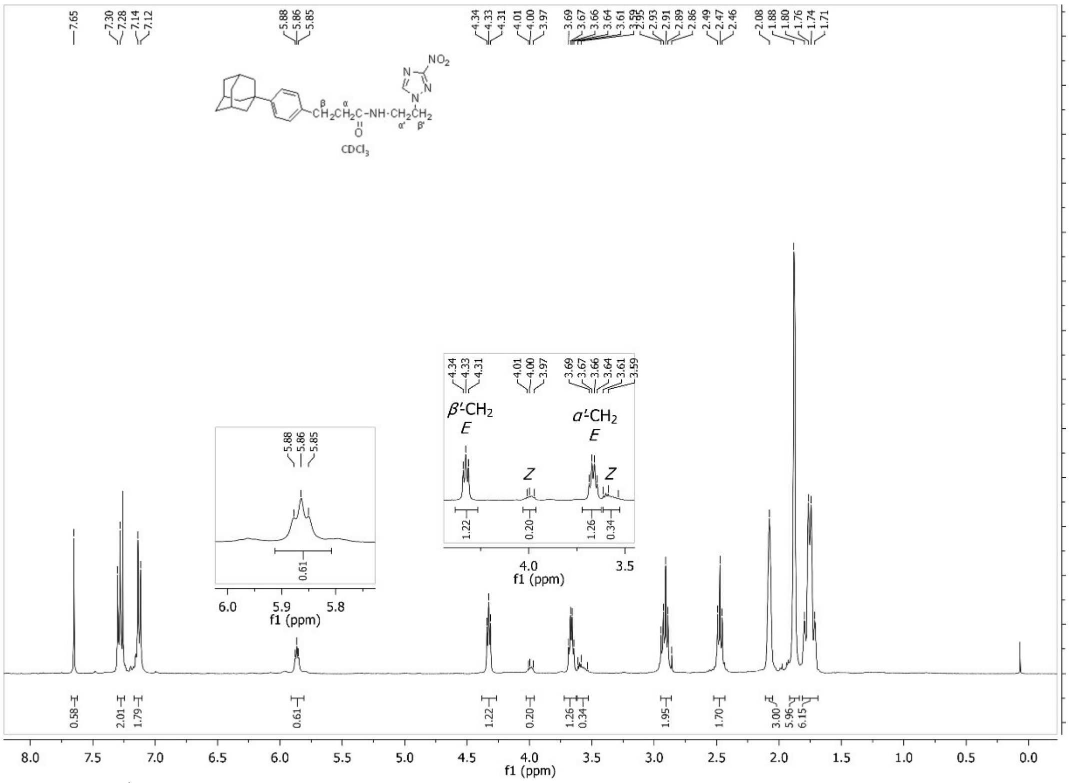

Figure S30. ^{1}H spectrum of **6c** in $CDCl_3$

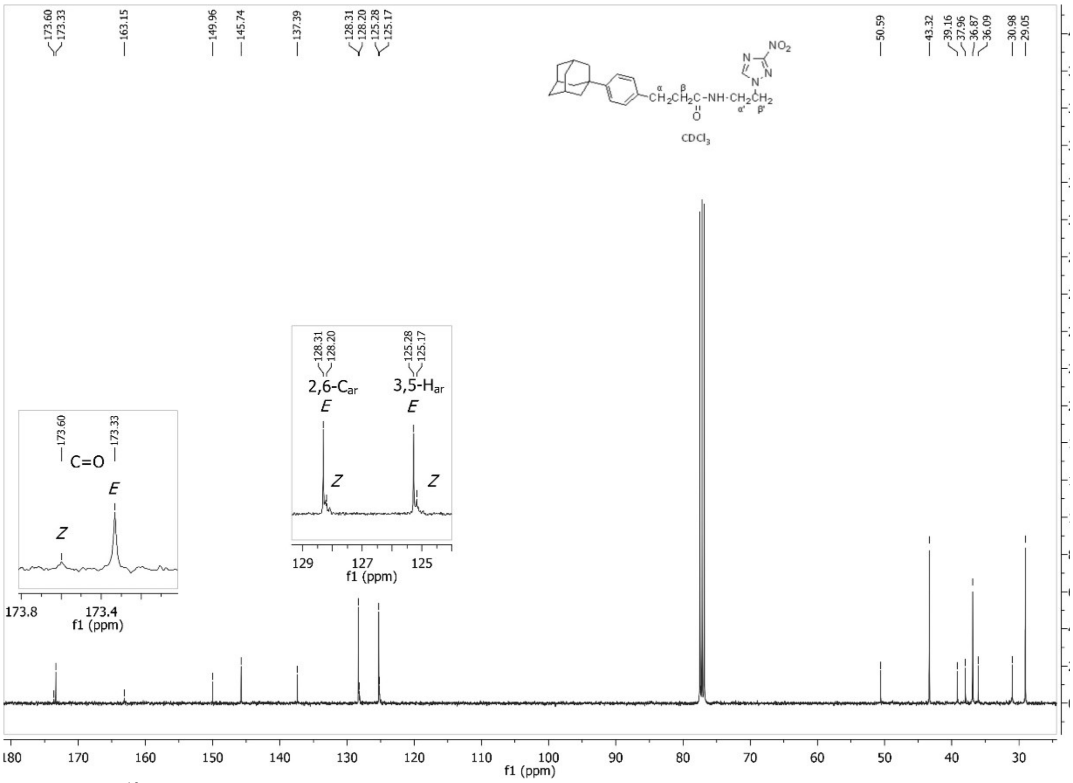

Figure S31. ^{13}C spectrum of **6c** in $CDCl_3$

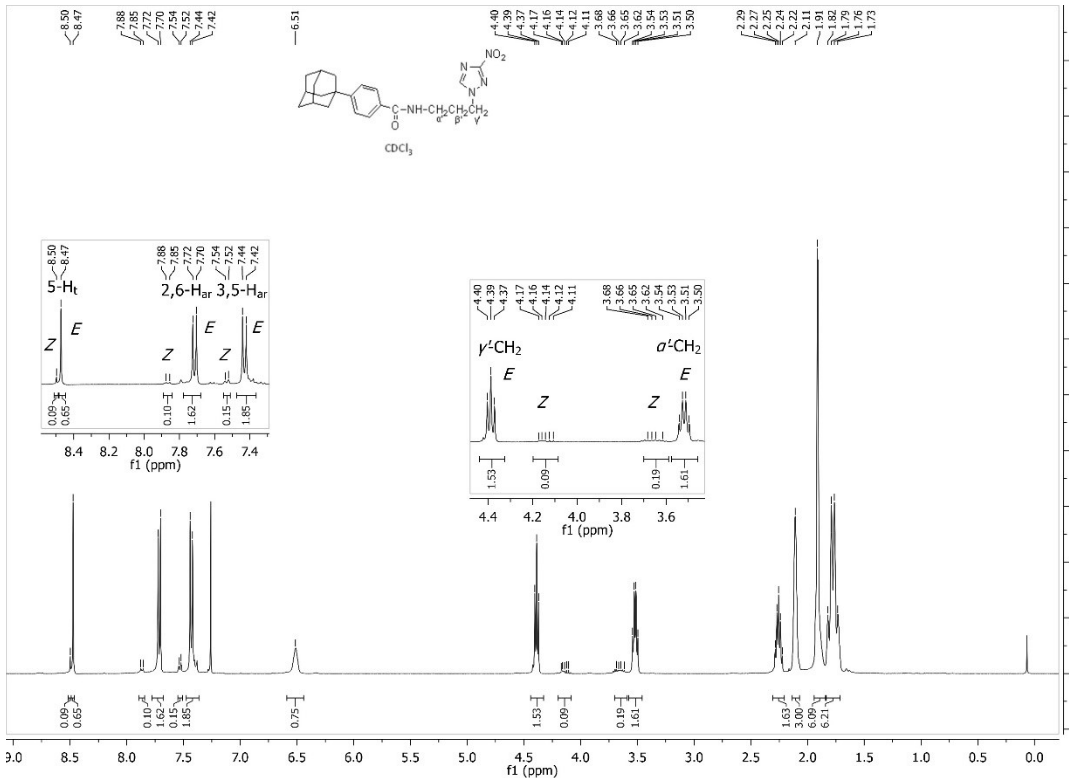

Figure S32. ^{1}H spectrum of **6d** in $CDCl_3$

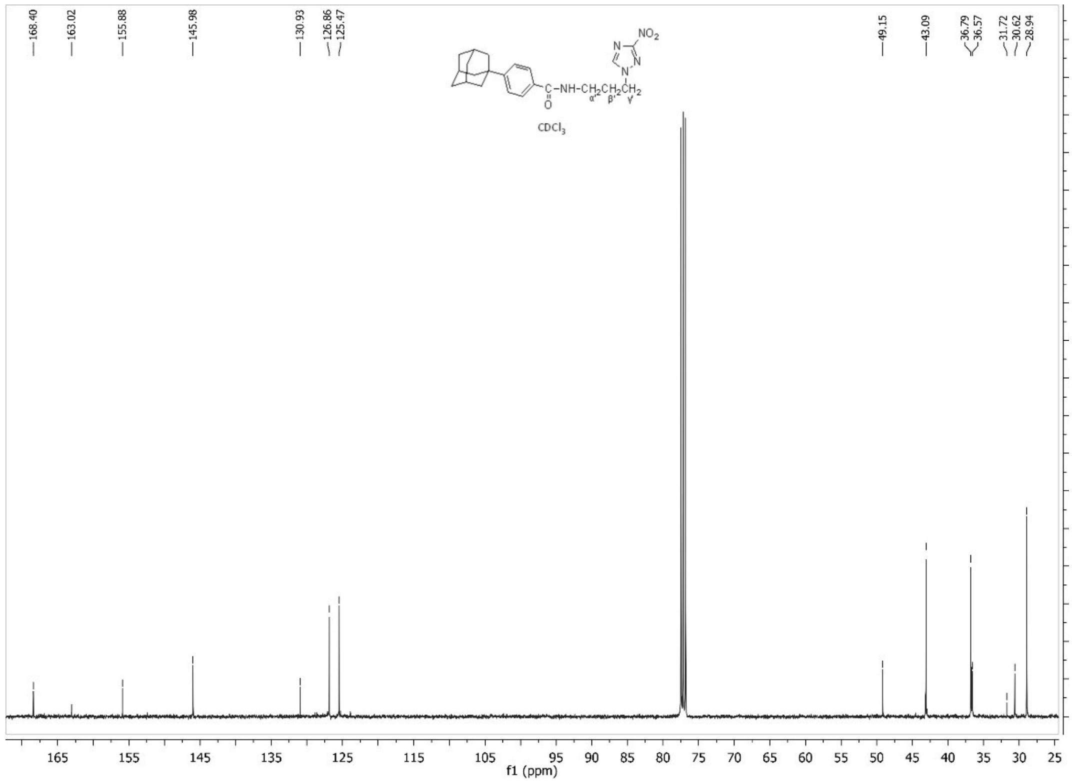

Figure S33. ^{13}C spectrum of **6d** in $CDCl_3$

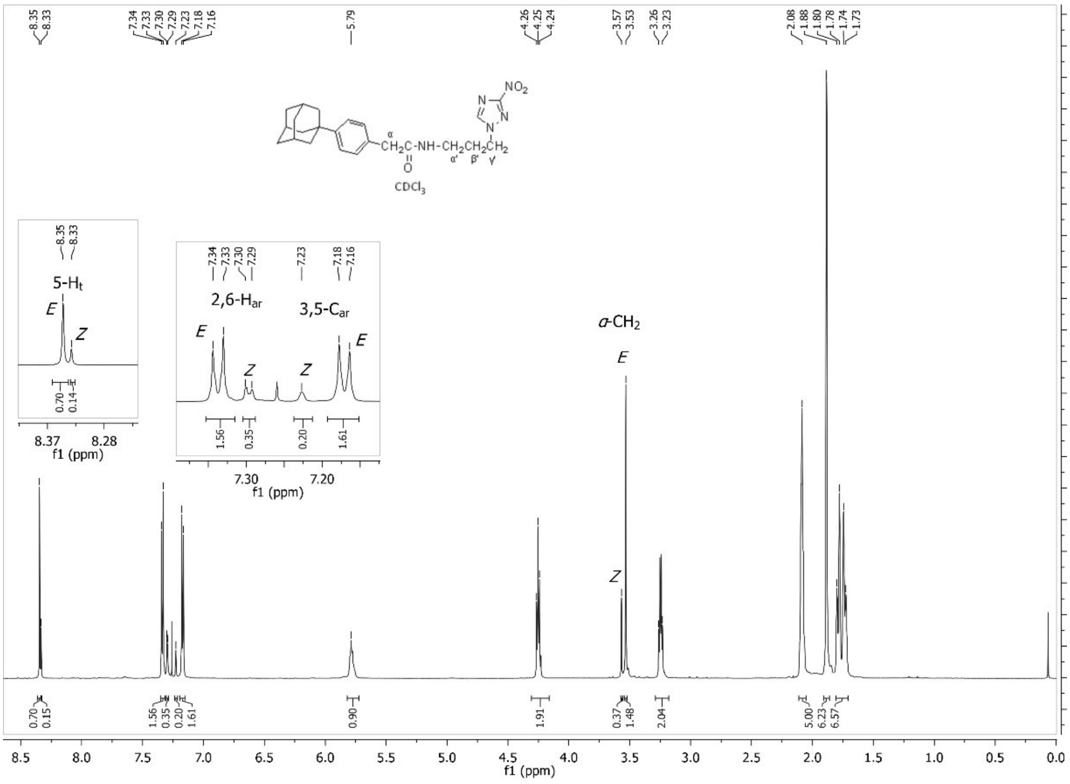

Figure S34. ^{1}H spectrum of **6e** in $CDCl_3$

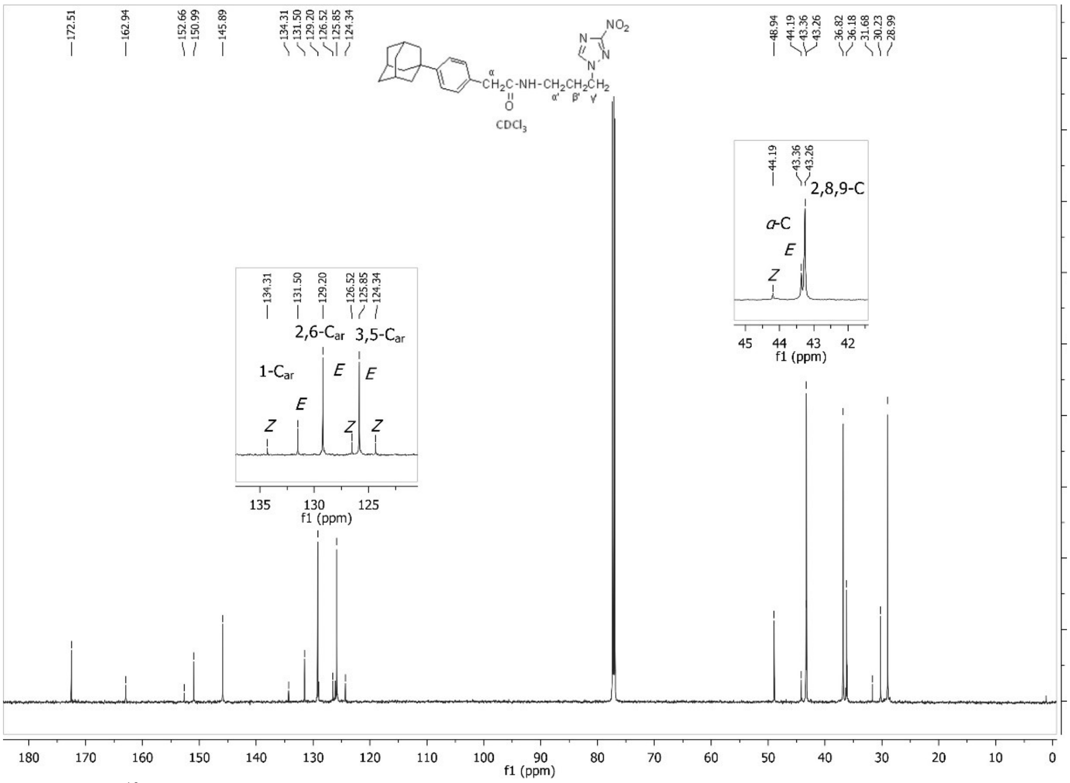

Figure S35. ^{13}C spectrum of **6e** in $CDCl_3$

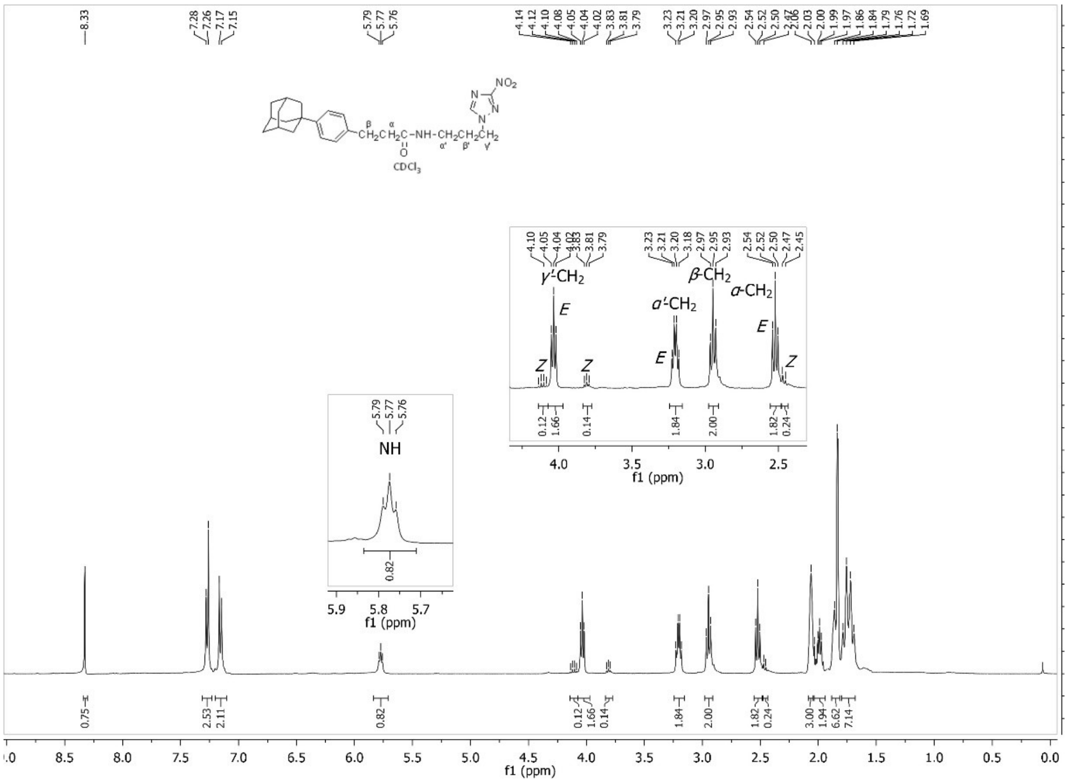

Figure S36. ^{1}H spectrum of **6f** in $CDCl_3$

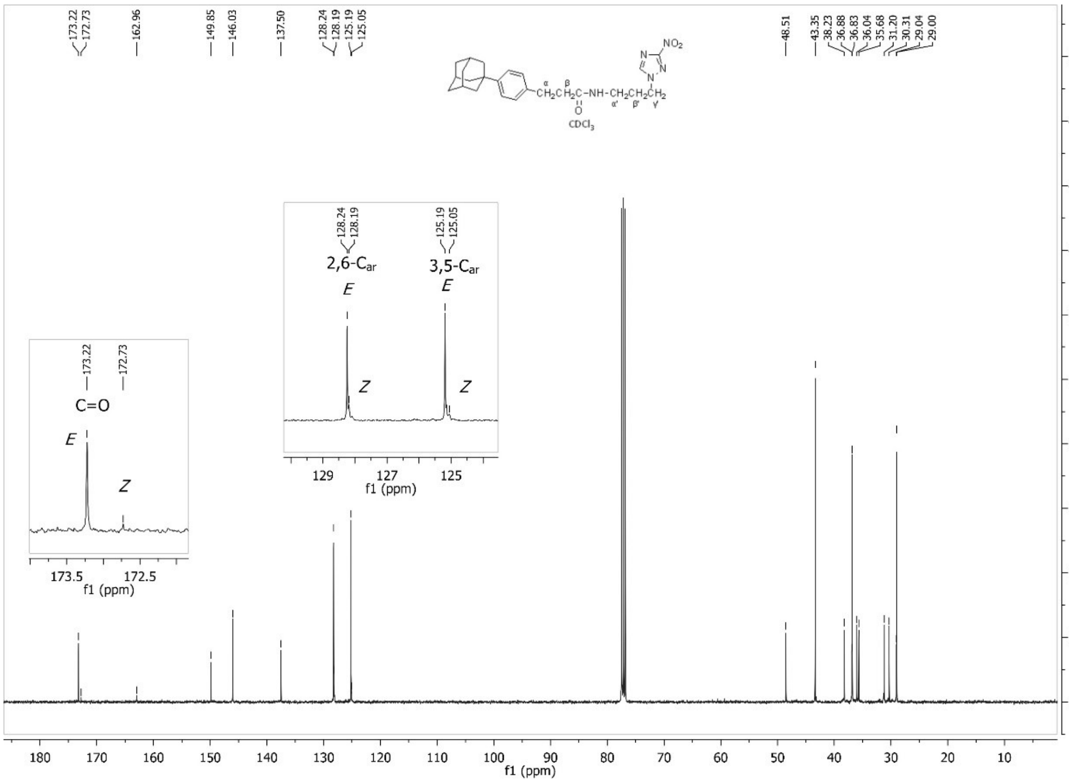

Figure S37. ^{13}C spectrum of **6f** in $CDCl_3$

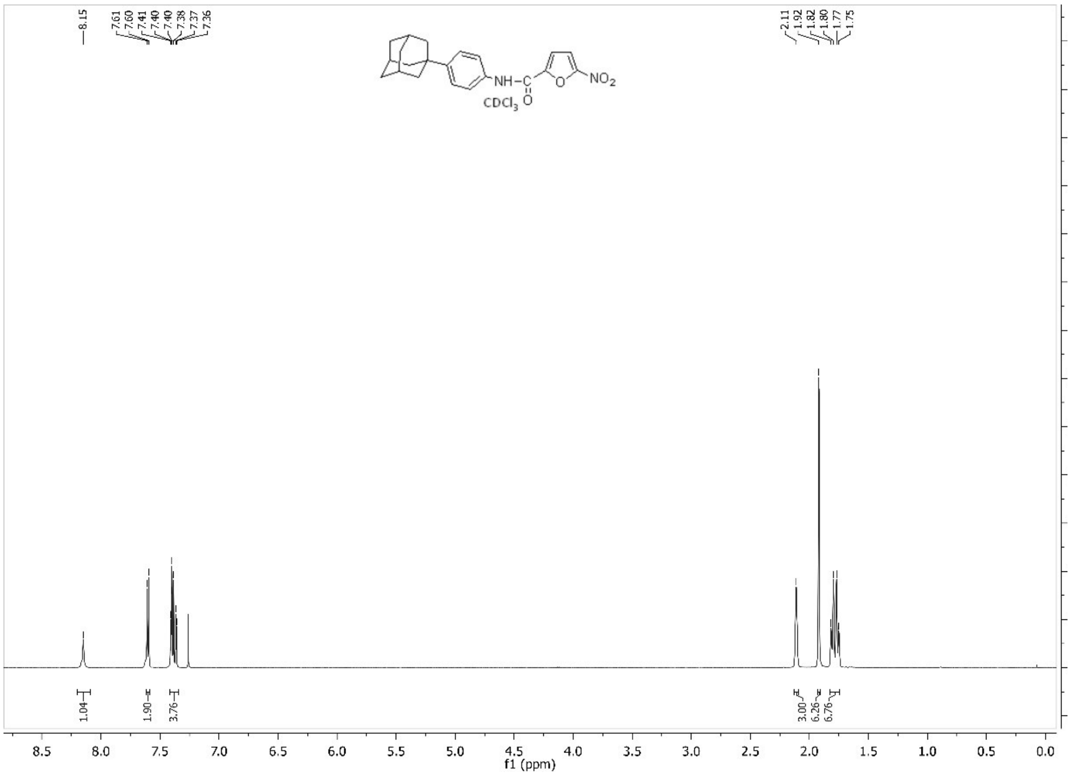

Figure S38. ^{1}H spectrum of **7a** in $CDCl_3$

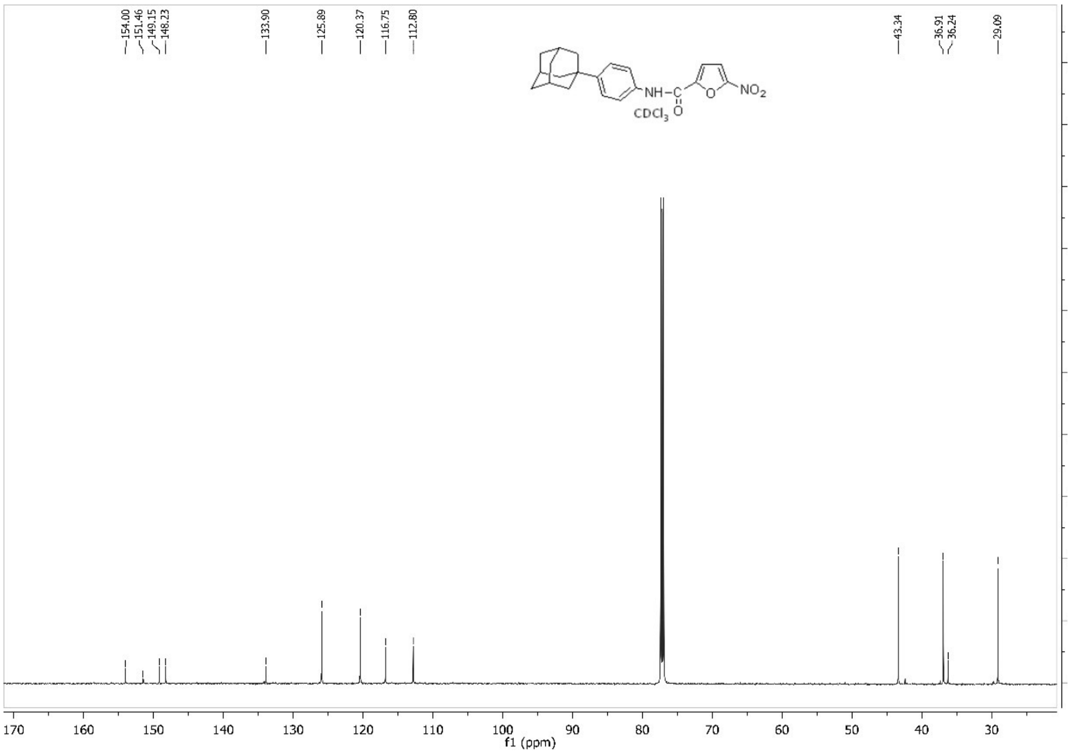

Figure S39. ^{13}C spectrum of **7a** in $CDCl_3$

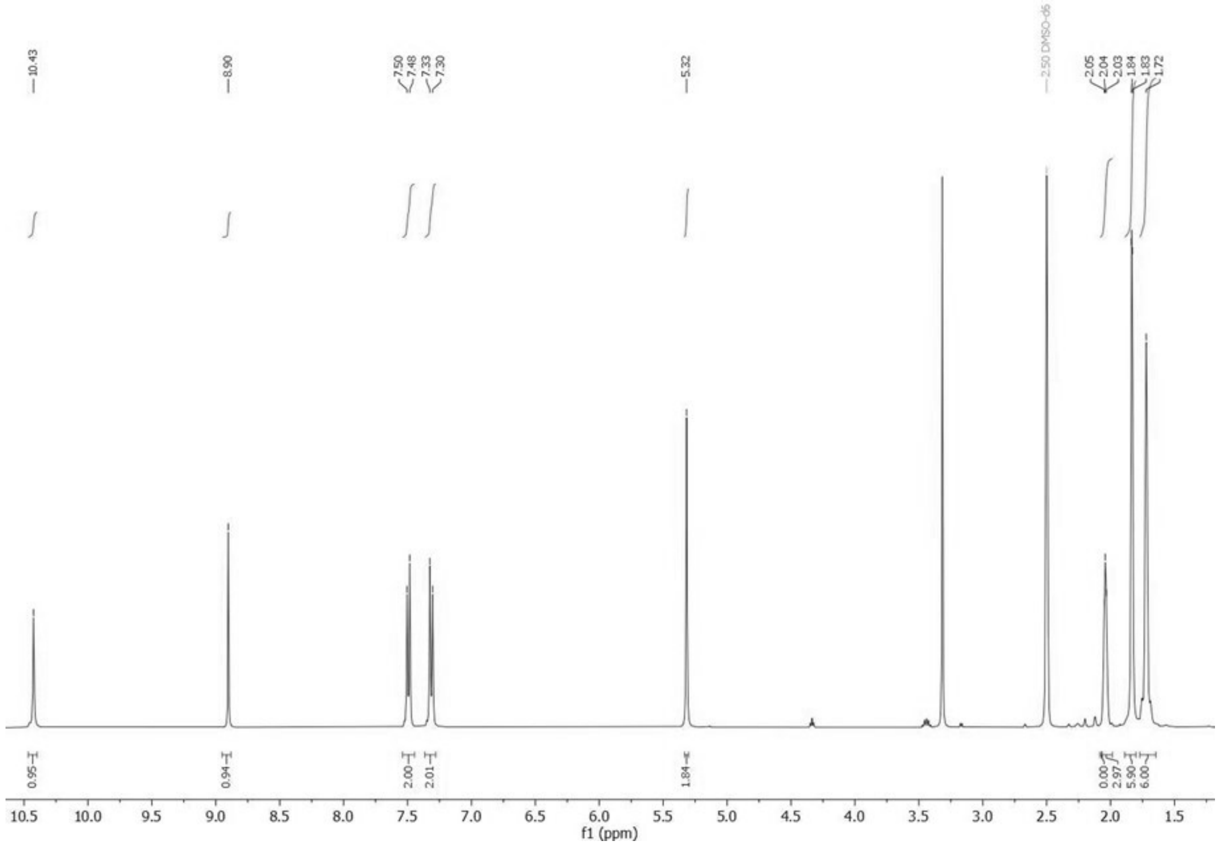

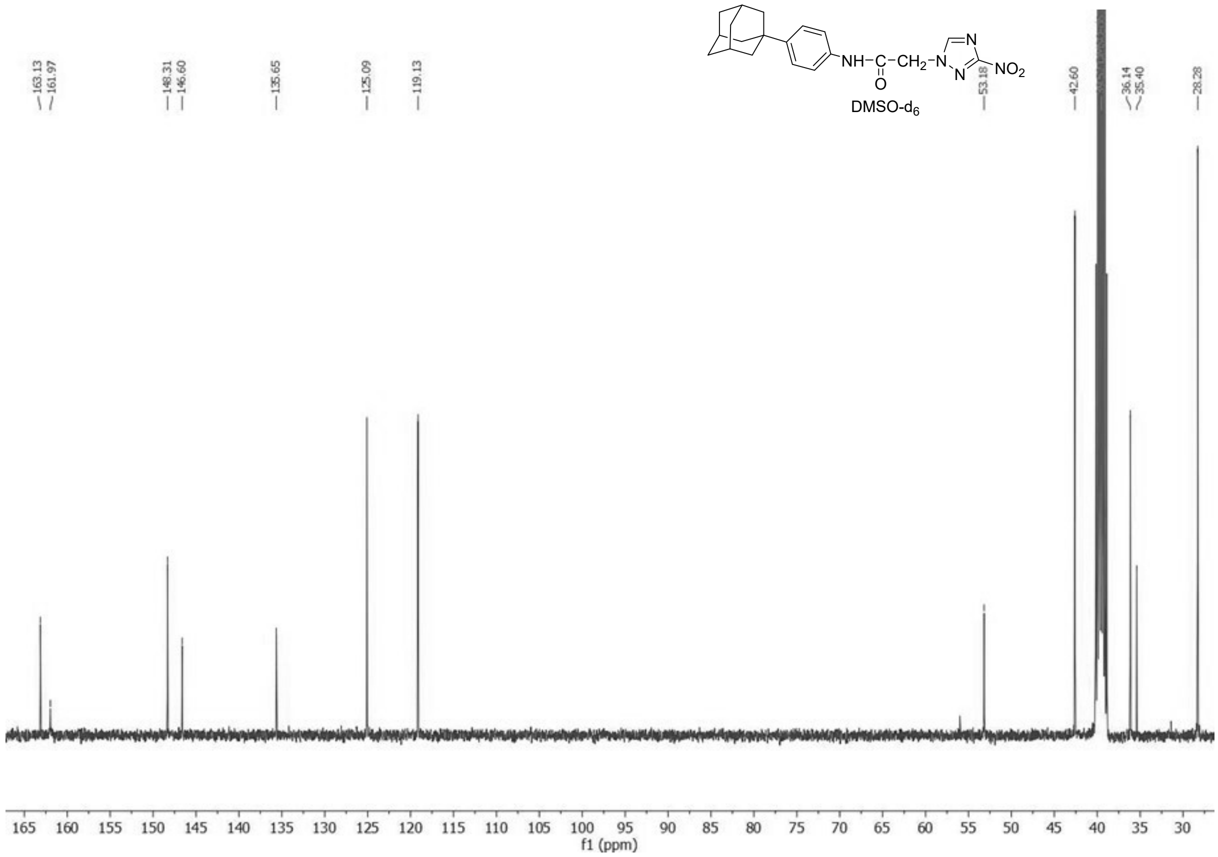

Figure S40. ^{1}H spectrum of **7b** in DMSO-d$_6$

Figure S41. ^{13}C spectrum of **7b** in DMSO-d$_6$

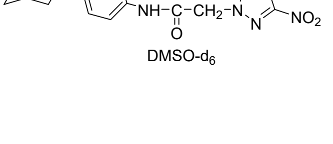

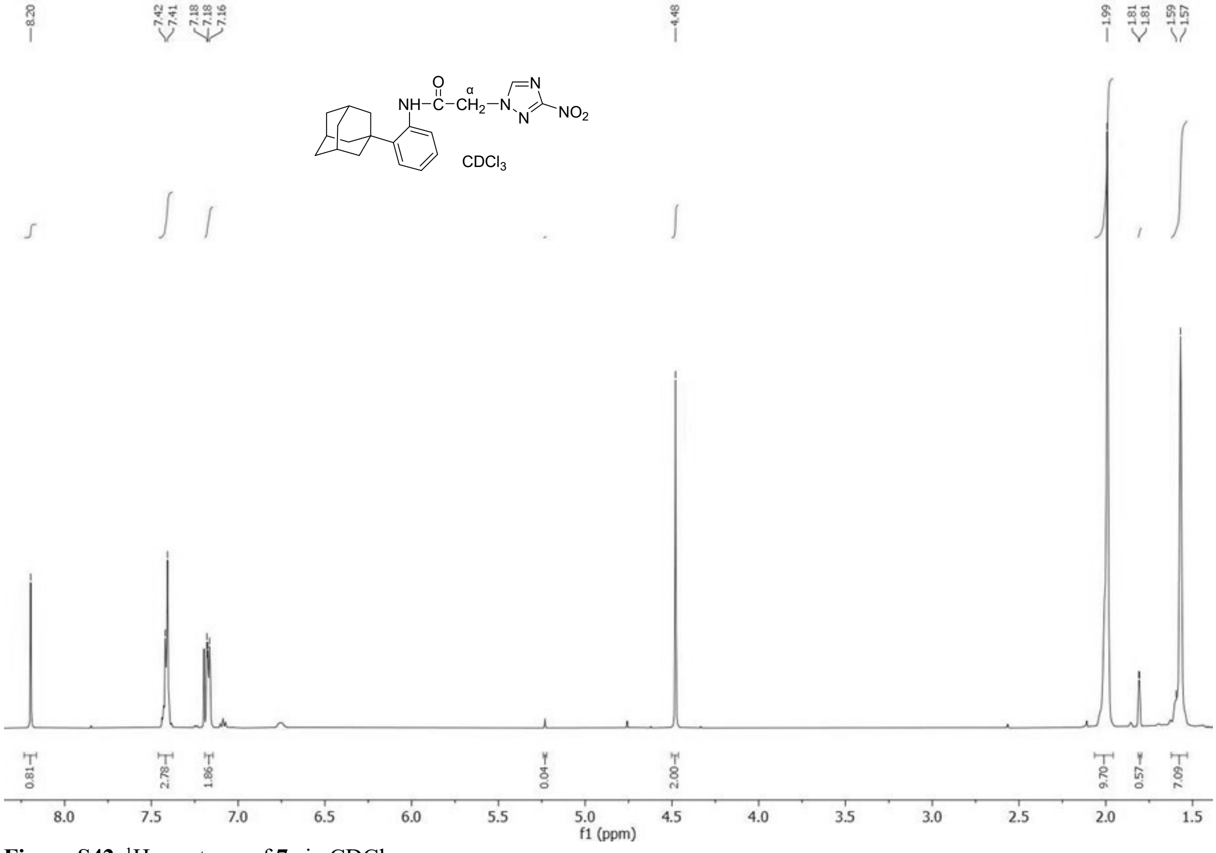

Figure S42. ^{1}H spectrum of **7c** in $CDCl_3$

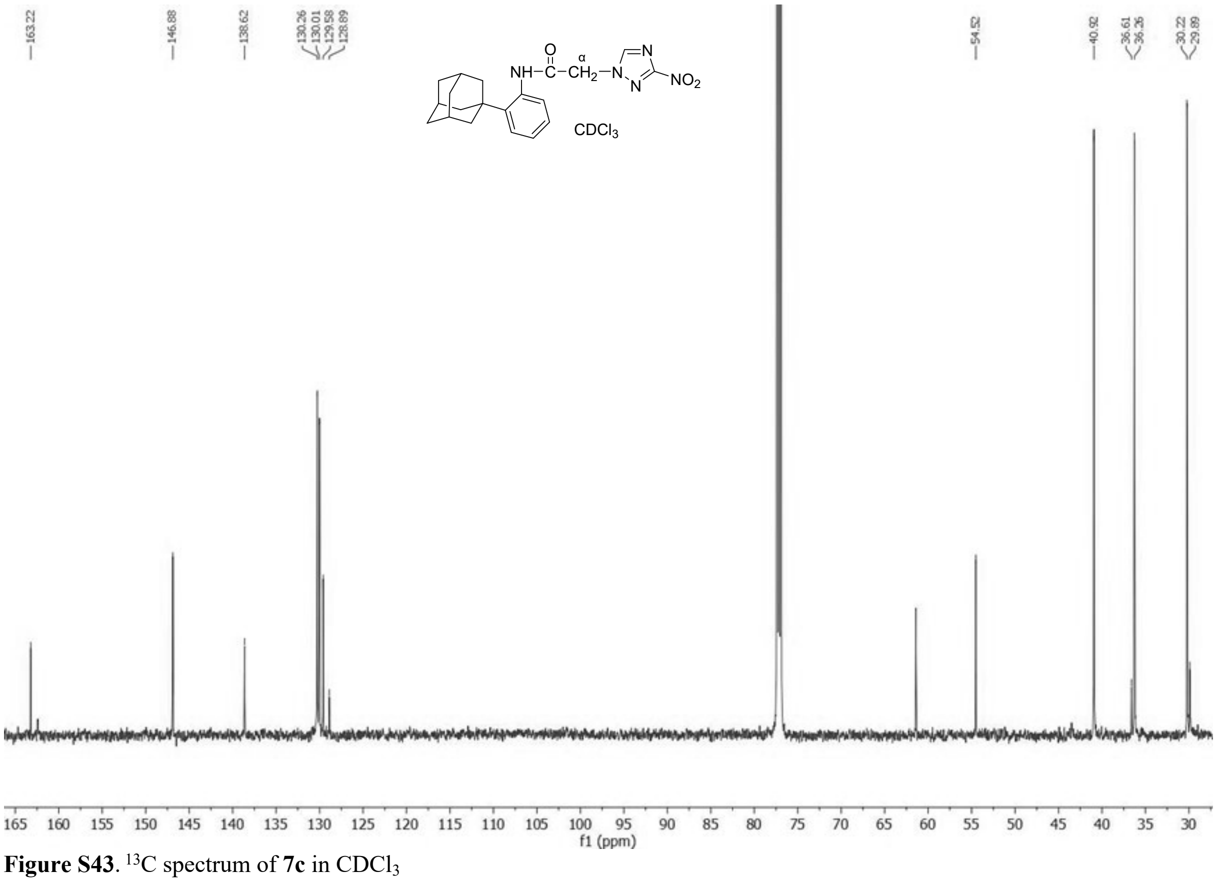

Figure S43. ^{13}C spectrum of **7c** in $CDCl_3$

References

1 I. Papanastasiou, S. Riganas, G. B. Foscolos, A. Tsotinis, S. Akhtar, M. Khan, K. Rahman and D. Thursto, *Lett Org Chem*, 2015, **12**, 319–323.
